# Anticoagulation Strategies in Adults Undergoing Extracorporeal Membrane Oxygenation: A Systematic Review and Meta‐Analysis

**DOI:** 10.1111/aor.70162

**Published:** 2026-05-20

**Authors:** Vincenz Scharner, Karoline Lenz, Harald Herkner, Nina Buchtele, Thomas Staudinger, Eva Schaden, Marion Wiegele, Johannes Gratz

**Affiliations:** ^1^ Department of Anesthesia, Intensive Care Medicine and Pain Medicine, Clinical Division of General Anesthesia and Intensive Care Medicine Medical University of Vienna Vienna Austria; ^2^ Department of Emergency Medicine Medical University of Vienna Vienna Austria; ^3^ Department of Medicine I, Intensive Care Unit 13i2 Medical University of Vienna Vienna Austria; ^4^ Ludwig Boltzmann Institute Digital Health and Patient Safety Medical University of Vienna Vienna Austria; ^5^ Department of Anesthesiology and Intensive Care Medicine, Campus Benjamin Franklin Charité – Universitätsmedizin Berlin Berlin Germany

**Keywords:** anticoagulation, bleeding, direct thrombin inhibitors, extracorporeal membrane oxygenation (ECMO), heparins, thrombosis

## Abstract

**Background:**

Extracorporeal membrane oxygenation (ECMO) is a life‐saving intervention for patients with severe cardiac or respiratory failure, but it is associated with a high risk of thrombotic and bleeding complications. Unfractionated heparin (UFH) remains the most frequently used anticoagulant, largely due to historical practice and longstanding clinical familiarity, despite the absence of robust evidence from randomized controlled trials. Alternative strategies—including direct thrombin inhibitors (DTIs), low‐molecular‐weight heparins (LMWHs), nafamostat mesylate (NM), and no anticoagulation—are increasingly being explored. However, a comprehensive and contemporary comparison of these approaches has been lacking.

**Methods:**

We conducted a comprehensive search of PubMed/MEDLINE, EMBASE, and CENTRAL through March 2025, supplemented by manual searches of reference lists. We included randomized controlled trials and observational studies including patients 16 years or older receiving ECMO for ≥ 24 h that compared any anticoagulation strategy with another or with no anticoagulation. The primary outcome was thromboembolic events; secondary outcomes included bleeding and mortality. Risk of bias was assessed using validated tools. Meta‐analyses were performed using a multivariable random‐effects model, with prespecified subgroup analyses by ECMO modality (venoarterial [VA], venovenous [VV], or mixed) and subsequent sensitivity analyses.

**Results:**

Twenty‐one observational studies involving 2 775 adult ECMO patients were included, with UFH serving as the comparator in all studies. DTIs showed reduced thromboembolism (OR 0.73; 95% CI: 0.53–0.99) and were associated with significantly lower bleeding (OR 0.51; 95% CI: 0.39–0.67) and mortality (OR 0.70; 95% CI: 0.52–0.94), confirmed in a sensitivity analysis. LMWH was associated with significantly reduced odds of thromboembolic events (OR 0.26; 95% CI: 0.13–0.55), as well as showing a favorable bleeding profile (OR 0.46; 95% CI: 0.25–0.87), yet no significant difference in mortality (OR 0.80; 95% CI: 0.30–2.14). While no anticoagulation reduced the odds for bleeding (OR 0.17; 95% CI: 0.07–0.38) and NM showed no significant differences, both had wide confidence intervals, limiting interpretation. Heterogeneity for LMWH was low for efficacy and bleeding, yet substantial for mortality; for DTI, low to moderate; for no anticoagulation, low; and for NM, substantial to high on all comparisons.

**Conclusions:**

In this systematic review and meta‐analysis, DTIs and LMWH appear to be effective and safe alternatives to UFH in adult ECMO, with consistent reductions in thromboembolic and bleeding events and a survival benefit observed for DTIs. These findings support reconsideration of UFH as the default anticoagulant and favor a more prominent role for alternative strategies in clinical practice.

**Trial Registration:**

PROSPERO CRD42022363588

## Graphical abstract

In this meta‐analysis of 2 775 adult ECMO patients from non‐randomized studies, direct thrombin inhibitors and low‐molecular‐weight heparins demonstrated a favorable efficacy and safety profile over unfractionated heparin. These findings suggest them to be effective alternatives to unfractionated heparin, underscoring the urgent need for randomized controlled trials in the field.

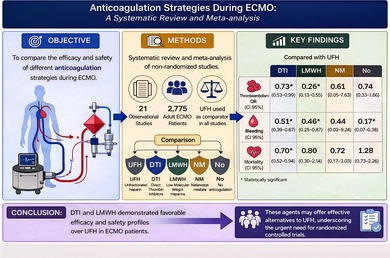

**Abbreviations**
CASP: Critical Appraisal Skills Programme
CI: Confidence intervals
DTI: Direct thrombin inhibitors
ECMO: Extracorporeal membrane oxygenation
ELSO: Extracorporeal Life Support Organization
HIT: Heparin‐induced thrombocytopenia and thrombosis
ICU: Intensive care unit
ISTH: International Society on Thrombosis and Hemostasis
LMWH: Low‐molecular‐weight heparins
NM: Nafamostat mesylate
OR: Odds ratio
PRISMA: Preferred Reporting Items for Systematic Reviews and Meta‐Analyses
PROSPERO: Prospective Register of Systematic Reviews
RCT: Randomized controlled trial
UFH: Unfractionated heparin
VA: Venoarterial
VV: Venovenous

## 1 Introduction

Extracorporeal membrane oxygenation (ECMO) provides life‐sustaining cardiopulmonary support for critically ill patients with severe cardiac and/or respiratory failure. However, ECMO is inherently associated with significant complications, most notably thrombosis and bleeding [1]. The risk of thrombotic events is heightened due to activation of the coagulation cascade upon blood contact with the artificial surfaces of the ECMO circuit [1]. Anticoagulant therapy mitigates this risk, as demonstrated by an inverse relationship between anticoagulation levels and thrombotic complications [2, 3]. However, anticoagulation simultaneously increases bleeding risk—a concern further amplified by ECMO‐induced depletion of coagulation factors and platelets [1]. Large registry data underscore the clinical relevance of this dilemma: In an analysis of patients supported with VV ECMO, approximately 40% experienced thromboembolic and/or bleeding events, both of which were independently associated with increased morbidity and mortality [4].

Unfractionated heparin (UFH) remains the most commonly used anticoagulant in ECMO and is currently recommended as the first‐line agent by both the *International Society on Thrombosis and Hemostasis* (ISTH) and *Extracorporeal Life Support Organization* (ELSO) [5, 6]. However, these recommendations have primarily been based on observational evidence, which has favored UFH due to historical practice, as well as longstanding clinical familiarity rather than high‐quality randomized controlled trial (RCT) data [7]. Despite its long‐standing use, UFH has several limitations, including the risk of heparin‐induced thrombocytopenia and thrombosis (HIT), variable pharmacokinetics and pharmacodynamics, antithrombin dependency, and a non‐linear dose–response relationship [8]. Consequently, direct thrombin inhibitors (DTIs) [7, 9], low‐molecular‐weight heparins (LMWHs) [10, 11, 12, 13], nafamostat mesylate (NM) [14, 15, 16], as well as no anticoagulation [3], have been explored as alternative anticoagulation strategies.

While prior systematic reviews and meta‐analyses have explored various aspects of anticoagulation in ECMO, no single systematic review and meta‐analysis has comprehensively compared all five strategies mentioned above. To address this gap, we conducted a systematic review and meta‐analysis to evaluate the comparative efficacy and safety of UFH, DTI, LMWH, NM, and no anticoagulation in adult ECMO patients. Specifically, we assessed (i) efficacy, defined as the prevention of thromboembolic events; and (ii) safety, defined as the risk of bleeding and all‐cause mortality associated with each anticoagulation strategy.

## 2 Methods

This systematic review was conducted in accordance with the Cochrane Handbook for Systematic Reviews of Interventions [17] and the Preferred Reporting Items for Systematic Reviews and Meta‐Analyses (PRISMA) guidelines [18]. The review protocol was registered with the International Prospective Register of Systematic Reviews (PROSPERO) under registration number CRD42022363588 on October 11, 2022.

### 2.1 Search Strategy, Study Selection, and Outcomes

A comprehensive literature search was developed and conducted by a trained medical librarian at the Medical University of Vienna. Searches were performed in PubMed/Medline, EMBASE, and CENTRAL databases using a predefined search strategy (Data S1). The search included all records up to March 17, 2025 (original search: October 2022; updates October 2023 and March 2025). Additionally, reference lists of all included articles, as well as systematic review citation lists, were screened manually. Records were managed using Zotero v6 (Corporation for Digital Scholarship, Vienna, USA).

Following the removal of duplicates, two reviewers (VS, KL) independently screened titles and abstracts for eligibility. Studies deemed potentially relevant, or in cases of reviewer disagreement, were retrieved for full‐text review and assessed independently by the same two reviewers. Discrepancies were resolved by consultation with a third senior reviewer (JG).

We included (i) randomized controlled trials, and (ii) non‐randomized (prospective or retrospective observational) studies that reported on patients 16 years or older receiving ECMO support for ≥ 24 h that fulfilled the eligibility criteria.

Eligible studies were required to compare any clearly defined anticoagulation strategy (i.e., single anticoagulant agent, including information on its dosing and/or monitoring target OR no systemic anticoagulation) with any comparator (e.g., another anticoagulant, placebo, or no anticoagulation) and report at least one of the following: **(*i) Primary outcome:*** Incidence proportion of thromboembolic events (i.e., the proportion of patients that developed at least one new‐onset thromboembolic event during ECMO). As the definition of thromboembolic events varied substantially across the included studies, we adopted a deliberately broad, inclusive definition aligned with the terminology and outcome reporting encountered in the literature. For the purposes of this review, thromboembolic events were defined as any newly developed thrombotic or embolic complication occurring during the ECMO run, as reported by the original study authors. This definition encompassed all clinically diagnosed, imaging‐confirmed, surgically identified, or autopsy‐detected thromboembolic events, regardless of specific anatomical site or diagnostic criteria used in individual studies. **(*ii) Secondary outcomes:*** Incidence proportion of bleeding events (i.e., the proportion of patients that developed at least one new‐onset bleeding event during ECMO), or mortality. Studies comparing one anticoagulation strategy (e.g., UFH) with another (e.g., DTI) following the occurrence of an outcome of interest (e.g., thromboembolic event due to HIT) were excluded. Studies were also excluded if patients switched between anticoagulation strategies, and outcomes could not be attributed to a specific treatment.

Notably, we deviated from the registered PROSPERO protocol by setting the lower age threshold for patients at 16 years instead of 18 years. This decision was made to avoid excluding relevant studies and is consistent with the ISTH recommendation [19] to apply adult coagulation reference ranges from age 16 due to physiological alignment at that stage.

In addition, because of the volume and heterogeneity of available literature, we limited inclusion to studies that reported comparative data (i.e., between different anticoagulation strategies or vs. no anticoagulation) to allow for meaningful meta‐analysis. Furthermore, we followed the Cochrane Handbook as outlined in the PROSPERO protocol, except for the risk of bias assessment, for which we used the CASP checklist.

### 2.2 Data Extraction and Risk of Bias Assessment

Two independent reviewers (VS, KL) extracted data using a standardized form. Extracted items included: Study characteristics (e.g., design, publication year, country), patient demographics (e.g., number of patients, age, sex), pertinent clinical information (e.g., ECMO indication and modality), and patient outcomes (e.g., thromboembolic events, bleeding, mortality, and incidence of HIT). Data accuracy and completeness were verified through comparison of the two independent extraction forms, with discrepancies resolved by a third reviewer (JG).

As no RCTs met the inclusion criteria, risk of bias for the included observational cohort and case–control studies was assessed using the appropriate Critical Appraisal Skills Programme (CASP) checklists [20] a validated tool for evaluating methodological quality.

### 2.3 Data Synthesis and Analysis

Pairwise meta‐analyses for each anticoagulation strategy compared with UFH were conducted using odds ratios (ORs) with 95% confidence intervals (CIs), estimated with the Mantel–Haenszel method under a random‐effects model to account for between‐study heterogeneity. A continuity correction was applied in studies with zero events.

Analyses were performed using the meta [21] package in R (R Core Team, R Foundation for Statistical Computing, Vienna, Austria). Effect estimates and corresponding 95% confidence intervals (CIs) were presented as forest plots for the following: (i) primary and efficacy outcome: Thromboembolic events, and (ii) secondary and safety outcomes: Bleeding events and mortality. Heterogeneity was assessed both clinically and statistically, with the I^2^ statistic used to quantify statistical heterogeneity. Where appropriate, subgroup‐specific meta‐analyses were performed. Given the clinical relevance of ECMO modality for both thromboembolic and bleeding events, we prespecified subgroup analyses for: (i) VA ECMO, (ii) VV ECMO, and (iii) mixed (both VV and VA) populations. Studies were categorized as (i) VA if > 80% of patients in both study arms were on VA ECMO, (ii) VV if < 20% were on VA ECMO, and (iii) mixed for all others. To investigate whether treatment effects differed according to ECMO type, random‐effects meta‐regression analyses were conducted with ECMO type (VV, VA, and mixed) included as a categorical moderator variable. Meta‐regression was performed on treatment‐specific pairwise meta‐analyses using log odds ratios as the dependent variable. Analyses were restricted to comparisons with at least two ECMO subgroups. Due to the small number of studies in several subgroups, meta‐regression results were interpreted as exploratory.

Where appropriate, *post hoc s*ensitivity analyses were performed. Specifically, the categorization of thromboembolic events into intracorporeal (patient‐related) and extracorporeal (device‐related) allowed for *post hoc* sensitivity analyses. Similarly, due to different mortality measures (e.g., ECMO mortality vs. one‐year mortality), we were able to conduct a *post hoc* sensitivity analysis for mortality. In addition, we performed a sensitivity analysis restricted to studies assessed as having a low risk of bias. Finally, given the known pharmacokinetic and pharmacodynamic differences between direct thrombin inhibitors—including differences in thrombin binding affinity, half‐life, and metabolic pathways—we conducted an additional *post hoc* sensitivity analysis in which the DTI group was separated into studies using bivalirudin, studies using argatroban, and studies reporting mixed or unspecified DTI use.

Data management and statistical analyses were conducted using Microsoft Excel (Microsoft, USA), R (R Core Team, Austria), and SPSS (IBM, USA). A two‐sided *p*‐value < 0.05 was considered statistically significant.

## 3 Results

### 3.1 Study Selection

**FIGURE 1:**
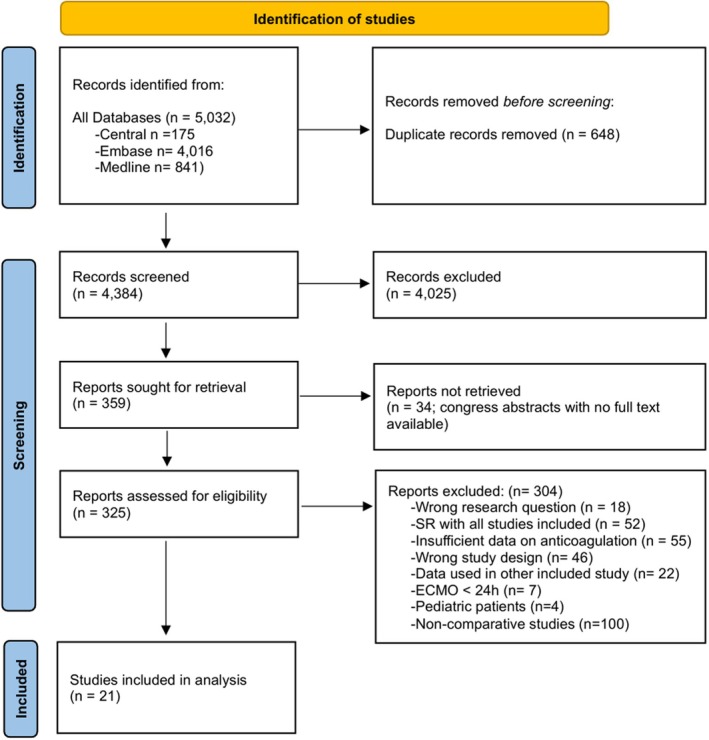
PRISMA flow chart. [Color figure can be viewed at wileyonlinelibrary.com]

The literature search included studies up to March 17, 2025, and identified 4 384 records after removal of duplicates. Of these, 21 studies met the eligibility criteria and were included in the analysis—16 from the initial search, and an additional 2 and 3 studies identified during updated screenings in October 2023 and March 2025, respectively. The study selection process is detailed in Figure 1. Studies excluded in full text are listed in Data S2.

### 3.2 Characterization of Included Studies

All 21 studies were observational in design and reported retrospectively collected data comparing two anticoagulation strategies as cohort studies, with UFH as the reference group in all cases. Collectively, the studies included 2 775 critically ill adult patients undergoing ECMO, with data spanning three continents: 11 studies from North America, 6 from Europe, and 4 from Asia. Among them, 1 579 patients received UFH, 799 received a DTI (508 Bivalirudin, 126 Argatroban, 165 unspecified DTI), 177 received LMWH, 136 received NM, and 84 received no anticoagulation.

Sample sizes ranged from 30 to 411 patients per study. Table 1 summarizes study characteristics, patient demographics, anticoagulation strategies, ECMO indications, and modalities. Notably, a substantial proportion of studies did not report key clinical variables, including age, sex, ECMO duration, or intensive care unit (ICU) length of stay.

**TABLE 1:** Study and patient characteristics.

Author (year)	Country	Intervention (*n*)	Coagulation monitoring	ECMO type	Percent VA	ECMO indication	Male sex	Age mean ± SD (years)
Berei [22]	USA	UFH: 28	aPTT 45–90 s/anti‐Xa 0.1–0.5 U/mL	VA: 26, VV: 2	92.9	Cardiogenic, septic, respiratory, or mixed shock	18 (64%)	55.9 (13.1)
2018		Bivalirudin: 44	aPTT 45–80 s	VA: 40, VV: 4	90		29 (66%)	55.2 (15.2)
Bermea [23]	USA	UFH: 25	aPTT 45–100 s	VV: 25	0	COVID ARDS	No information	No information
2021		Bivalirudin or Argatroban: 5	aPTT 45–100 s	VV: 5	0		No information	No information
Burša [24]	Czech Republic	UFH: 14	aPTT 45–60 s	VV: 14	0	COVID ARDS	6 (43%)	Med (Min; Max) 53 (31; 69)
2024		Argatroban: 49	aPTT 45–60 s	VV: 49	0		32 (65%)	Med (Min; Max) 48 (27; 65)
Cho [25]	USA	UFH: 24	aPTT 40–80 s	VA: 8, VV: 16	33.3	No information	15 (63%)	45 ± 16
2021		Argatroban: 11	aPTT 60–80 s	VA: 6, VV:5	54.5		7 (64%)	49 ± 20
Fisser [26]	Germany	UFH: 78	aPTT 45–55 s	VV: 78	0	ARDS	51 (65%)	Med (IQR) 56 (48;63)
2021		Argatroban: 39	aPTT 45–55 s	VV: 39	0		29 (74%)	Med (IQR) 55 (46; 61)
Gratz [10]	Austria	UFH: 22	aPTT 1.5 x baseline	VA: 7, VV: 15	31.8	Lung transplant patients	12 (55%)	41 ± 14
2019		Enoxaparin: 80	0.5 mg/kg bid, no monitoring	VA: 64, VV: 16	80		43 (54%)	40 ± 15
Han [15]	Korea	NM: 17	aPTT 60–80 s	VA: 14, VV: 3	82.4	AMI, cardiac arrest, cardiogenic shock, septic shock, ARDS	17 (100%)	49.2 ± 17.4 \
2011		UFH: 51	ACT 140–180 s	VA: 45, VV:6	88.2		32 (63%)	61.7 ± 15.1
Kartika [27]	USA	UFH: 61	anti‐Xa 0.2–0.5 U/mL	VV: 61	0	ARDS	42 (69%)	45 ± 14.2
2024		Bivalirudin: 34	aPTT 60–75 s	VV: 34	0		20 (59%)	44 ± 12.1
Kaseer [28]	USA	UFH: 33	aPTT 1.5‐2 x baseline	VA:22, VV:11	66.6	Cardiogenic shock, respiratory failure, heart and/or lung transplant	25 (76%)	Med (range) 53 (21–83)
2020		Bivalirudin: 19	aPTT 1.5‐2 x baseline	VA:6, VV: 13	31.6		12 (63%)	Med (range) 56 (18–71)
Lim [16]	Korea	UFH: 201	ACT 160–220 s aPTT 50–70 s	VA: 201	100	Post‐cardiotomy shock	128 (64%)	59.1 ± 14.4
2016		NM: 119	ACT 160–220 s/aPTT 50–70 s	VA: 119	100		74 (62%)	57.7 ± 16.7
Mayerhöfer [29]	Austria	UFH: 30	Anti‐Xa 0.2–0.3 U/mL	VV: 30	0	COVID ARDS	19 (63%)	Med (IQR) 57 (51–62)
2024		Argatroban: 27	Hemoclot 0.4–0.6 μg/mL	VV: 27	0		21 (78%)	Med (IQR) 54 (50–61)
Piwowarczyk [12]	Poland	Nadroparin: 35	5700 IU qd, no monitoring	VV: 35	0	Invasive aspergillosis, bacterial or viral pneumonia, ARDS associated with acute pancreatitis, ARDS associated with postoperative mediastinitis, lung injury, COPD	28 (80%)	Med (IQR) 50.3 [41–58.5]
2021		UFH: 32	aPTT 60–80 s/ACT 140–160 s	VV: 32	0		25 (78%)	Med (IQR) 46.8 [35–59]
Raman [30]	USA	UFH: 50	ACT 180–220 s	VA: 50	100	Respiratory indications, post‐cardiotomy, cardiorespiratory arrest, eCPR, bridge to cardiac intervention/surgery, cardiogenic shock, sepsis	31 (62%)	Mean (range) 54.8 (20–81)
2019		no AC: 52	no monitoring	VA: 52	100		34 (65%)	Mean (range) 53.6 (19–82)
Rivosecchi [31]	USA	UFH: 162	anti‐Xa 0.25–0.35 U/mL	VV: 162	0	Acute respiratory failure, pre−/post‐lung transplant, post‐thoracic surgery	95 (59%)	Med (IQR) 49 (36–61)
2021		Bivalirudin: 133	aPTT 61–75 s	VV: 133	0		81 (61%)	Med (IQR) 49 (36–61)
Saeed [32]	USA	UFH: 251	aPTT 50–70 s	VA: 14, VV:237	5.6	COVID ARDS	178 (71%)	Med (IQR) 48 (38–57)
2022		DTI: 160	aPTT 50–70 s	VA: 4, VV:156	2.5		116 (72%)	Med (IQR) 49 (37–57)
Seelhammer [33]	USA	UFH: 223	aPTT 60–90 s/ACT 180–220 s/K‐TEG 16‐29 min	VA: 184, VV: 39	83	Post‐cardiotomy, cardiac, respiratory, eCPR, post‐transplant	148 (66%)	No information
2021		Bivalirudin: 110	aPTT 60–90 s	VA: 93, VV:17	85		69 (63%)	No information
Sheridan [34]	USA	UFH: 50	anti‐Xa 0.25–0.7 U/mL	VA: 25, VV: 25	50	Cardiogenic Shock, respiratory failure, pulmonary embolism, failure to wean from CPB	32 (64%)	53 ± 14
2022		Bivalirudin: 100	aPTT 45–75 s	VA: 63, VV: 37	63		74 (74%)	54 ± 15
Song [35]	Korea	UFH: 99	aPTT 60–80 s	VA: 90, VV: 9	90.9	Ischemic cardiogenic shock, other	No information	No information
2023		No AC: 32	no monitoring	VA: 27, VV: 5	84.4		No information	No information
Tong [36]	China	UFH: 20	aPTT 1.5‐2 x baseline	VV: 20	0	Non‐COVID ARDS	11 (55%)	50.51 ± 15.52
2023		Bivalirudin: 14	aPTT 1.5‐2 x baseline	VV: 14	0		11 (79%)	52.82 ± 8.13
Uricchio [37]	USA	Bivalirudin: 54	aPTT 61–75 s	VA: 54	100	Refractory cardiogenic shock	39 (72%)	Med (IQR) 53 (42–62)
2022		UFH: 89	anti‐Xa0.25–0.35 U/mL	VA: 89	100		63 (71%)	Med (IQR) 53 (40–60)
Wiegele [11]	Austria	Enoxaparin: 62	Anti‐Xa 0.3–0.5 U/ml	VA: 1, VV: 61	1.6	COVID ARDS	48 (77%)	Med (IQR) 57 (53–62.8)
2022		UFH: 36	aPTT 50–60 s/anti‐Xa 0.2–0.3 U/mL	VA: 3, VV: 3	8.3		21 (58%)	Med (IQR) 57 (50.8–61)

Abbreviations: AC, Anticoagulation; ACT, activated coagulation time; AMI, acute myocardial infarction; anti‐Xa, anti‐factor Xa activity levels; aPTT, activated partial thromboplastin time; ARDS, acute respiratory distress syndrome; bid, bis in die; COPD, chronic obstructive pulmonary disease; COVID‐ 19, corona virus disease 2019; CPB, cardiopulmonary bypass; DTI, direct thrombin inhibitors; ECMO, extracorporeal membrane oxygenation; ECPR, extracorporeal cardiopulmonary resuscitation; IQR, inter‐quartile range; KTEG, kaolin thromboelastography; Max, maximum; Med, median; Min, minimum; NM, nafamostat mesylate; qd, quaque die; SD, standard deviation; UFH, unfractionated heparin; VA, venoarterial; VV, venovenous.

For the prespecified subgroup analysis, 7 studies were categorized in the VA ECMO subgroup, [15, 16, 22, 30, 33, 35, 37] 10 studies in the VV ECMO subgroup [11, 12, 23, 24, 26, 27, 29, 31, 32, 36] and 4 studies in the mixed population subgroup [10, 25, 28, 34].

**FIGURE 2:**
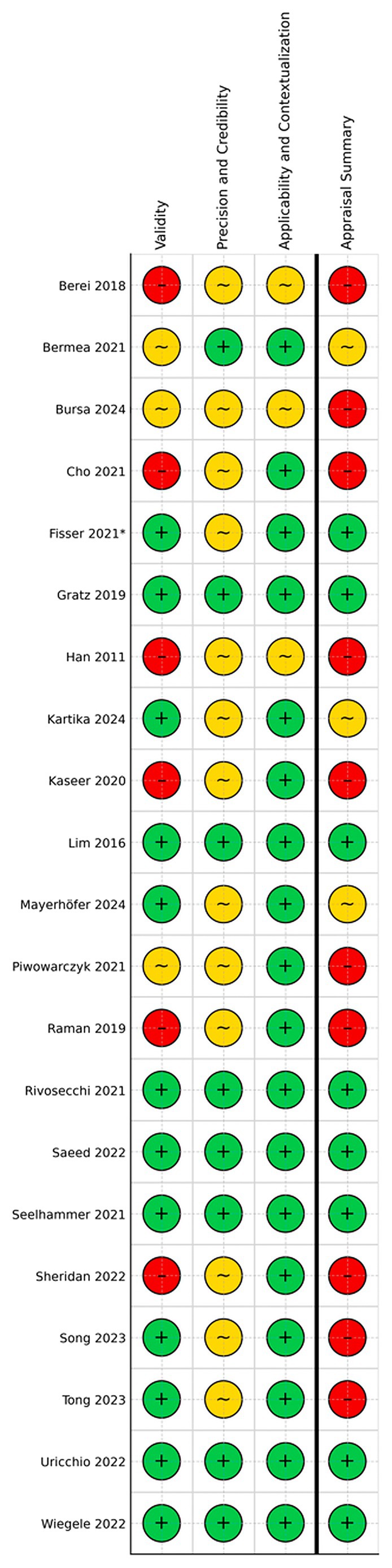
Risk of bias assessment. +, low risk of bias; ~, moderate risk of bias; −, high risk of bias. *Study assessed using the appropriate CASP tool for case–control studies. [Color figure can be viewed at wileyonlinelibrary.com]

Risk of bias was assessed using the CASP checklist, which evaluates studies using the following 3 categories: (i) validity, (ii) precision and credibility, and (iii) applicability and contextualization. Based on these 3 categories, studies were rated as having a high, moderate, or low risk of bias. Ten studies were rated as high risk of bias, three as moderate risk, and eight as low risk. The complete quality assessment is shown in Figure 2.

### 3.3 Primary Efficacy Outcome: Thromboembolism

Nineteen studies reported thromboembolic outcomes, enabling estimation of odds ratios (OR) comparing different anticoagulant strategies to UFH. Specifically, 12 studies compared DTI to UFH, [22, 24, 25, 26, 27, 28, 31, 32, 33, 34, 36, 37] 3 compared LMWH to UFH, [10, 11, 12] 2 compared NM to UFH, [15, 16] and 2 compared no anticoagulation to UFH [30, 35].

**FIGURE 3:**
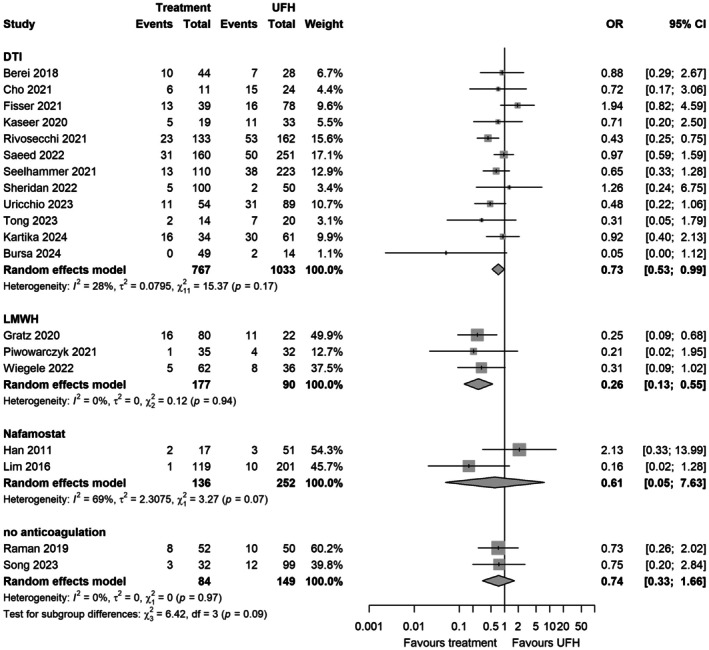
Forest plots for efficacy by intervention.

In the random‐effects meta‐analyses (Figure 3), both LMWH and DTI were associated with significantly lower odds of thromboembolic events compared to UFH (LMWH OR 0.26; 95% CI: 0.13–0.55; DTI OR 0.73; 95% CI: 0.53–0.99). NM and no anticoagulation showed no differences, and both demonstrated wide confidence intervals. Heterogeneity was low for no anticoagulation and LMWH (I^2^ = 0%), moderate for DTI (I^2^ = 28%), and substantial for NM (I^2^ = 64%).

In the prespecified subgroup analyses by ECMO modality (7 studies in the VA ECMO subgroup, [15, 16, 22, 30, 33, 35, 37] 8 studies in the VV ECMO subgroup, [11, 12, 24, 26, 27, 31, 32, 36] and 4 studies in the mixed population subgroup [10, 25, 28, 34]), the direction and magnitude of treatment effects were generally comparable to the overall estimates. In exploratory meta‐regression analysis, ECMO type was not significantly associated with the treatment effect for DTI (p for interaction = 0.239). For LMWH, meta‐regression suggested a statistically significant difference in treatment effect according to ECMO type (p for interaction = 0.019, Data S3).

### 3.4 Secondary Safety Outcomes: Bleeding and Mortality

Bleeding outcomes were reported in 19 studies [10, 11, 12, 15, 16, 23, 24, 25, 27, 28, 29, 31, 33, 34, 35, 36, 37] and mortality in 20 studies [10, 11, 12, 15, 16, 22, 24, 25, 26, 27, 28, 29, 30, 31, 32, 33, 34, 35, 36, 37].

#### 3.4.1 Bleeding

Twelve studies compared DTI to UFH, [22, 23, 24, 25, 26, 27, 28, 31, 32, 34, 36, 37] 3 compared LMWH to UFH, [10, 11, 12] 2 compared NM to UFH, [15, 16] and 2 compared no anticoagulation to UFH [30, 35].

**FIGURE 4:**
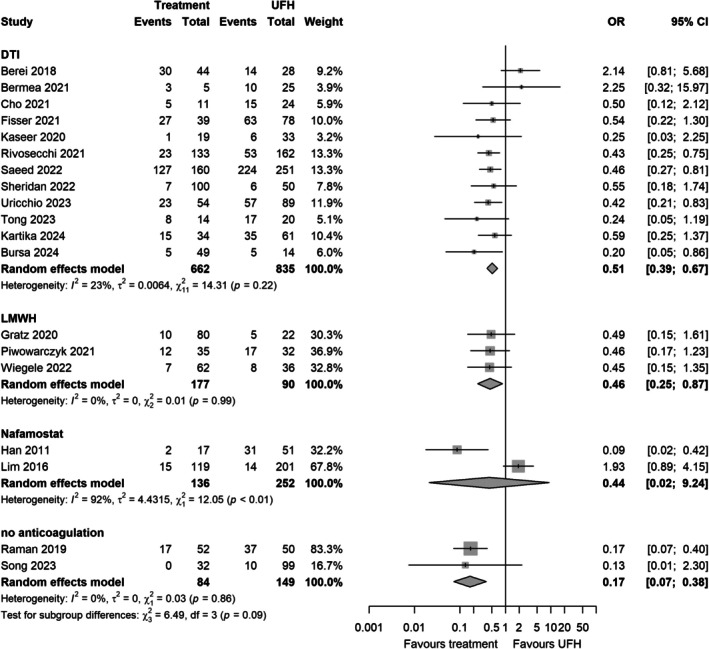
Forest plots for incidence of bleeding events by intervention.

In the random‐effects meta‐analyses, DTI, LMWH, and no anticoagulation were associated with reduced odds of bleeding, while NM did not display reduced odds for bleeding compared to UFH. Heterogeneity was low for no anticoagulation and LMWH (I^2^ = 0%), as well as for DTI (I^2^ = 43%), while being high for NM (I^2^ = 92%) (Figure 4).

In prespecified subgroup analyses (6 studies in the VA ECMO subgroup, [15, 16, 22, 30, 35, 37] 9 studies in the VV ECMO subgroup, [11, 12, 23, 24, 26, 27, 31, 32, 36] and 4 studies in the mixed population subgroup, [10, 25, 28, 34]), the direction and magnitude of treatment effects on bleeding events were generally consistent with the overall estimates, except for DTI in the VA ECMO subgroup. In exploratory meta‐regression analyses, ECMO type was significantly associated with treatment effect for DTI (*p* < 0.001), indicating substantial differences in effect estimates across VA, VV, and mixed ECMO populations. A statistically significant association was also observed for LMWH (*p* = 0.036, Data S4).

#### 3.4.2 Mortality

Mortality definitions varied across studies: Eleven studies reported hospital mortality as the longest reported mortality measure [22, 24, 25, 26, 27, 28, 29, 32, 33, 35, 36], 4 ICU mortality [10, 11, 12, 30], 2 ECMO mortality [34], 2 one‐year mortality [31, 37] and 1 study reported 60‐day mortality [15]. Thirteen studies compared DTI to UFH [22, 24, 25, 26, 27, 28, 29, 31, 32, 33, 34, 36, 37], 3 compared LMWH to UFH [10, 11, 12], 2 compared NM to UFH [15, 16], and 2 compared no anticoagulation to UFH [30, 35].

**FIGURE 5:**
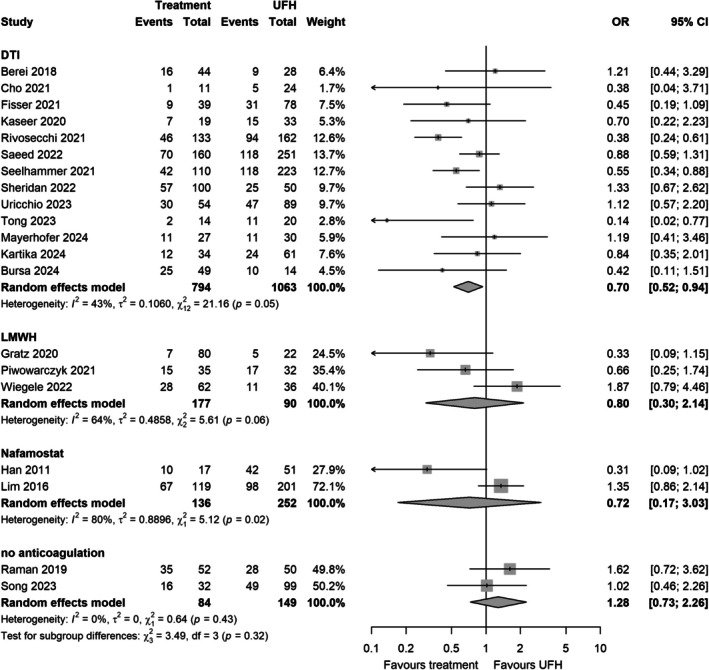
Forest Plots for longest reported mortality.

Figure 5 presents the odds ratios for the longest reported mortality measure. In these analyses, DTI was associated with reduced mortality (OR 0.70; 95% CI: 0.52–0.94) while other groups showed no significant difference compared to UFH. Heterogeneity was low for no anticoagulation (I^2^ = 0%), moderate for DTI (I^2^ = 43%), substantial for LMWH (I^2^ = 64%), and high for NM (I^2^ = 80%).

### 3.5 Sensitivity Analyses

To assess the robustness of efficacy outcomes, thromboembolic events were categorized as (i) intracorporeal events (e.g., pulmonary embolism, deep vein thrombosis, stroke, intracardiac thrombus), and (ii) extracorporeal events (e.g., oxygenator, pump, or cannula thrombosis). Fourteen studies reported on intracorporeal events, [10, 11, 15, 22, 25, 26, 27, 28, 30, 32, 33, 34, 35, 37] while 13 studies reported on extracorporeal events [10, 11, 22, 24, 25, 26, 27, 28, 30, 31, 34, 35, 37].

Sensitivity analysis showed consistent results for DTI (OR 0.83 vs. 0.81) and LMWH (OR 0.39 vs. 0.28), though confidence intervals widened for both when extracorporeal events were excluded. The odds estimate for no anticoagulation shifted notably (OR 0.99 vs. 0.77). Data for NM were insufficient for stratified analysis (Data S5).

Due to the considerable differences in ECMO mortality and further mortality measures, a separate sensitivity analysis included only the 10 studies reporting ECMO mortality [10, 11, 15, 16, 24, 25, 27, 30, 31, 37]. Findings remained relatively stable for all interventions with DTI, LMWH, and NM showing a reduction in mortality and no anticoagulation showing increased odds compared to UFH (Data S6).

To further assess the robustness of our findings, we performed a sensitivity analysis restricted to studies judged to be at low risk of bias. Seven such studies reported on bleeding [10, 11, 16, 26, 31, 32, 37], with eight reporting on thromboembolic events and mortality [33]. Concerning thromboembolic events, effect estimates for DTI and LMWH remained consistent with the primary analysis (DTI OR 0.73 vs. 0.74; LMWH OR 0.26 vs. 0.27), although only LMWH retained statistical significance. NM showed a further widening of the already non‐significant confidence interval. For mortality, the direction of effect for DTI and LMWH remained consistent with the main analysis; however, confidence intervals widened. The estimate for NM shifted from an OR of 0.72 to 1.35, again with broad and overlapping confidence intervals. Regarding bleeding events, the results for DTI, LMWH, and NM remained consistent with the primary analysis, showing a reduction for DTI, a trend toward reduction for LMWH, and little change for NM. No studies evaluating a no‐anticoagulation strategy were classified as low risk of bias and were therefore not included in this analysis (Data S7).

Furthermore, in a *post hoc* sensitivity analysis, the DTI group was separated into studies reporting on bivalirudin [22, 27, 28, 31, 33, 34, 36, 37], argatroban [24, 25, 26, 29], and mixed/unspecified DTI, [23, 32]. For bleeding, effect estimates were comparable to the main analysis (primary analysis OR 0.51; argatroban OR 0.43; bivalirudin OR 0.55; mixed DTI OR 0.76). Similarly, mortality results showed a consistent direction of effect (primary analysis OR 0.70; argatroban OR 0.59; bivalirudin OR 0.70; mixed DTI OR 0.88), but none reached statistical significance. For thromboembolic events (primary analysis OR 0.73), bivalirudin (OR 0.59) continued to demonstrate reduced odds, with non‐significant results for argatroban and mixed DTI (OR 0.97) (Data S8).

## 4 Discussion

This systematic review and meta‐analysis provide comprehensive evidence on the comparative efficacy and safety of five anticoagulation strategies in adult patients receiving ECMO support. We evaluated (i) UFH, (ii) DTI, (iii) LMWH, (iv) NM, and (v) no anticoagulation. Our findings suggest that the choice of anticoagulation strategy significantly influences thrombotic and bleeding outcomes, with important implications for clinical decision‐making.

A recent analysis of the ELSO registry, encompassing over 7 000 patients on VV ECMO, revealed that 40.2% experienced thromboembolic and/or bleeding events, both of which significantly contributed to morbidity and mortality [4]. These findings underscore the critical need to optimize anticoagulation strategies to balance thrombotic and hemorrhagic risks in ECMO patients. Yet, evidence to guide optimal anticoagulation strategies remains limited. Current guidelines recommend UFH as the first‐line agent, largely based on clinical experience and its historical use, rather than data from RCTs [5, 6]. Our review confirms that, to date, no head‐to‐head RCTs, as well as no prospective trials, exist that directly compare different anticoagulants in ECMO. This highlights the need to generate randomized controlled evidence, while in the meantime considering emerging evidence from observational studies, as done in our present meta‐analysis. Consistent with UFH's current role as the international standard, it was used as the comparator in all included studies.

DTIs, including argatroban and bivalirudin, are regularly used as alternatives to UFH, particularly in patients with confirmed or suspected HIT or other contraindications for UFH. Against this background, their use as alternative anticoagulants is also proposed by both ELSO and ISTH [5, 6]. Our meta‐analysis demonstrated a consistently favorable safety profile for DTIs, with reduced odds of bleeding and mortality, confirmed in sensitivity analyses, as well as a significant efficacy difference between DTI and UFH for thromboembolic events. In part, our findings are consistent with previous systematic reviews and meta‐analyses comparing the use of argatroban and bivalirudin with UFH in both adult and pediatric ECMO populations. For example, Li et al. [38], reported lower rates of major bleeding and in‐hospital mortality with bivalirudin compared to UFH, with no significant difference in clinical thrombotic events. Chen et al. [39], found reduced odds of thrombosis with bivalirudin, whereas argatroban did not confer the same benefit. Neither DTI was associated with a reduction in bleeding or mortality in that analysis. Notably, in contrast to our study, Chen et al. included pediatric populations and did not exclude patients who were switched between anticoagulants (e.g., after thromboembolic events due to HIT), which may partly account for differences in specific outcomes, despite a broadly similar conclusion: That DTIs may offer a valid alternative to UFH for anticoagulation in ECMO. Geli et al. [40], reported comparable rates of thromboembolic and bleeding events between argatroban and UFH, although their analysis did not include a formal meta‐analysis. Given their shared mechanism of action and the fact that two studies did not distinguish between bivalirudin and argatroban use, we chose to analyze DTIs as a unified group to better capture overall trends and facilitate meaningful comparisons in this clinically important area. However, owing to their different pharmacological properties, we performed post hoc sensitivity analyses for bivalirudin and argatroban separately, which largely confirmed the findings of our primary analysis, whereas argatroban alone showed no difference with regard to thromboembolism in comparison to UFH. A previous meta‐analysis that used DTI as an overarching category in comparison to UFH found a similar reduction in mortality and bleeding events [41]. Notably, a significant limitation of most observational studies comparing DTI and UFH is their use of a before‐and‐after design, whereby earlier cohorts received UFH and later cohorts were treated with DTI. This design introduces substantial bias, as ECMO centers typically gain clinical experience and increase volume over time, which has been consistently associated with improved patient outcomes, e.g., in an analysis of the ELSO registry [42], an evaluation of VA ECMO cases in Germany [43], and an overall analysis of ECMO centers across the US [44].

LMWH, which has largely replaced UFH in the general ICU population [45], represents a newer option in the ECMO setting. Our findings underscore its potential value, demonstrating significantly greater efficacy in reducing thromboembolism with significantly fewer bleeding complications compared to UFH. With more predictable pharmacokinetics and ease of administration in comparison to UFH, LMWH appears to be a promising, yet potentially underutilized, strategy in ECMO anticoagulation. However, this interpretation must be contextualized by the fact that only 177 of the 2 775 patients in our analysis received LMWH, and the three contributing studies had mixed risk‐of‐bias profiles. For example, in Piwowarczyk et al. [12], patients were treated with LMWH at the largest ECMO center, while data on UFH use originated from substantially smaller centers, introducing a relevant risk of bias. Furthermore, while sensitivity analyses separating intra‐ and extracorporeal thromboembolism confirmed the efficacy of LMWH, exclusion of intracorporeal events resulted in substantially wider confidence intervals. In conjunction with a lack of standardized outcomes of interest—e.g., definitions of circuit thrombosis or bleeding events—care should be taken in drawing firm conclusions from these data.

Although its ultra‐short half‐life makes it potentially advantageous in patients at high risk of bleeding, evidence for NM as an alternative to UFH remains limited. We found no significant differences in any outcomes, and the wide confidence intervals, unstable sensitivity analysis results, and substantial to high heterogeneity in all meta‐analyses suggest a high degree of uncertainty. Furthermore, to date, the use of NM during ECMO has only been described in Korean patients, which further limits the generalizability of these results. In line with our findings, the systematic review by Sanfilippo et al. [46], reports conflicting results for both thrombosis and bleeding with the use of NM during ECMO.

Interestingly, the strategy of no anticoagulation showed similar efficacy in preventing thromboembolic events to UFH, with the expected reduction in odds of bleeding and no difference in mortality. This suggests that withholding anticoagulation may be appropriate in select clinical scenarios—such as traumatic brain injury—though similarly to NM (136 patients), the small sample sizes (no anticoagulation: 84 patients) warrant caution in interpretation.

Although VA and VV ECMO patients typically differ in underlying pathologies and associated odds for both thrombosis and bleeding, subgroup analyses revealed that treatment effects were broadly similar across ECMO configurations and aligned with the overall estimates, except for DTI, where the incidence of bleeding events was higher in the VA subgroup. Although exploratory meta‐regression analyses suggested possible differences in treatment effects across VA, VV, and mixed ECMO subgroups, these findings should be interpreted cautiously due to the limited number of patients within each comparison.

In summary, our findings align with previous meta‐analyses highlighting potential advantages of DTIs over UFH, especially in reducing circuit and patient‐related thrombotic events without increasing bleeding risk. Of note, our study expands on this by incorporating LMWH as an additional viable comparator and providing evidence for the potential role of no anticoagulation in select clinical scenarios.

This study has several limitations: First, all included studies were retrospective observational cohorts, inherently subject to selection bias, confounding, and inconsistent outcome definitions. Although some studies attempted adjustment, the lack of RCTs limits the certainty of our conclusions, which is mirrored by our risk‐of‐bias assessment. Second, there was significant heterogeneity in ECMO modalities (VA vs. VV), patient populations (e.g., COVID‐19 patients vs. surgical patients), and anticoagulation protocols. This variability may limit generalizability to specific clinical contexts or institutional practices. Third, the reporting of key outcomes—such as thrombosis and bleeding severity—was inconsistent. Definitions of major bleeding and circuit thrombosis varied, and some studies lacked detailed information on anticoagulation monitoring and dose adjustments, complicating interpretation and preventing a more granular depiction of outcomes in this meta‐analysis, such as the severity of bleeding. Similarly, relevant confounding factors, such as ECMO flow rates or the concomitant use of antiplatelet agents, could not be accounted for in this analysis. Fourth, our analysis of LMWH, nafamostat, and no anticoagulation relied on a limited number of studies and patients, which constrains the strength of inferences regarding their comparative efficacy and safety. These limitations underscore the urgent need for well‐designed, prospective RCTs to define optimal anticoagulation strategies in ECMO. Such trials should use standardized outcome definitions and stratify by confounding factors, such as ECMO modality.

## 5 Conclusion

This systematic review and meta‐analysis challenge the default use of UFH in ECMO. DTIs and LMWH were consistently associated with reduced thromboembolic and bleeding events, with DTIs also showing a survival advantage, supporting their consideration as preferred alternatives in adult ECMO. Uncertainty remains primarily for less‐studied strategies and across ECMO subtypes, emphasizing the urgent need for targeted comparative trials with standardized outcome definitions and modality‐specific analyses to inform definitive practice change.

## Author Contributions

V.S., K.L., and J.G. were involved in data collection and contributed to the quality assessment of included studies. V.S. and H.H. conducted the statistical analysis. V.S. and J.G. designed the study and drafted the manuscript. All authors were (i) involved in the study design, (ii) critically revised the study protocol and manuscript for important intellectual content, and (iii) reviewed and approved the manuscript prior to its submission.

## Funding

The study is partly funded by the European Society of Intensive Care Medicine (ESICM) Clinical Research Award 2022 (JG).

## Consent

The authors have nothing to report.

## Conflicts of Interest

T.S. has received speaker fees from CSL Behring, Fresenius, Getinge, and Mitsubishi Tanabe as well as investigator‐initiated research grants from CSL Behring and Mitsubishi Tanabe. N.B. has received speaker fees from Mitsubishi Tanabe and investigator‐initiated research grants from CSL Behring and Mitsubishi Tanabe. J.G. has received honoraria, research funding, and travel reimbursement from Alexion, AstraZeneca, Boehringer Ingelheim, CSL Behring, Instrumentation Laboratory, Johnson & Johnson, Mitsubishi Tanabe Pharma, Octapharma, Portola, and Takeda. E.S. has received speaker fees from CSL Behring and AstraZeneca and financial support for the Podcast “ClotTalk” from Arjo Austria, Roche Diagnostics, CSL Behring, Biomedica, AstraZeneca, Novo Nordisk Pharma, Mitsubishi Tanabe Pharma, and AOP Orphan Pharmaceuticals. VS, K.L., H.H., and M.W. declare no competing interests.

## Supporting information


**Data S1:** Search strategy used for PubMed/MEDLINE, EMBASE, and CENTRAL.

**Data S2:** Excluded in the full text with a reason.

**Data S3:** Efficacy by intervention: Subgroup analysis.

**Data S4:** Incidence of bleeding events by intervention: Subgroup analysis.

**Data S5:** A Forest Plot for efficacy by intervention: Intracorporeal thrombosis.

**Data S6:** Forest‐plots‐ECMO‐Mortality‐by‐intervention.

**Data S7:** Forest plots for efficacy, mortality, and bleeding by intervention, low Risk of Bias.

**Data S8:** Forest plots for efficacy, mortality, and bleeding by intervention with split Direct Thrombin Inhibitor group.

## Data Availability

The data that support the findings of this study are available from the corresponding author upon reasonable request.
