# Supplementary material for: Anticoagulation Strategies in Adults Undergoing Extracorporeal Membrane Oxygenation: A Systematic Review and Meta‐Analysis
Source: Artif Organs. 2026 May 20;50(8):1103–17. doi: 10.1111/aor.70162 (PMC13452911; doi:10.1111/aor.70162)
Supplement: Supplementary file 1 [file AOR-50-1103-s002.pdf]

Additional File 2: Excluded in full text with reason

Author	Study	Reason for exclusion
**Abbasciano RG, et al[1]**	Blood Transfusion Threshold in Patients Receiving Extracorporeal Membrane Oxygenation Support for Cardiac and Respiratory Failure—A Systematic Review and Meta-Analysis	Insufficient data on anticoagulation
**Abruzzo A, et al[2]**	Venous thromboembolic events in the setting of extracorporeal membrane oxygenation support in adults: A systematic review	Insufficient data on anticoagulation
**Ahmad SB, et al[3]**	Extracorporeal membrane oxygenation after traumatic injury	Wrong study design
**Akhtar W, et al[4]**	SARS-CoV-2 and ECMO: early results and experience	Insufficient data on anticoagulation
**Al Abbasi B, et al[5]**	Veno-Arterial Extracorporeal Membrane Oxygenation Support (VA-ECMO) in acute coronary syndrome complicated by cardiogenic shock	Wrong study design
**Al-Jazairi A, et al[6]**	Performance Assessment of a Multifaceted Unfractionated Heparin Dosing Protocol in Adult Patients on Extracorporeal Membrane Oxygenator	Non-comparative study
**Alkazemi A, et al[7]**	Conventional versus restricted anti-Xa-guided heparin protocol in adult patients undergoing extracorporeal membrane oxygenation	Non-comparative study
**Amos T, et al[8]**	ECMO (extra corporeal membrane oxygenation) in major trauma: A 10 year single centre experience	Insufficient data on anticoagulation
**Anderson H, et al[9]**	Extracorporeal life support for adult cardiorespiratory failure	Wrong study design
**Ang AL, et al[10]**	Blood transfusion requirements and independent predictors of increased transfusion requirements among adult patients on extracorporeal membrane oxygenation - A single centre experience	Non-comparative study
**Arachchillage DJ, et al[11]**	Anticoagulation with argatroban in patients with acute antithrombin deficiency in severe COVID-19	Insufficient data on anticoagulation
**Arachchillage DJ, et al[12]**	Intracranial Hemorrhage and Early Mortality in Patients Receiving Extracorporeal Membrane Oxygenation for Severe Respiratory Failure	Non-comparative study
**Arlt M, et al[13]**	Extracorporeal membrane oxygenation in severe trauma patients with bleeding shock	Non-comparative study
**Arnouk S, et al[14]**	Evaluation of Anti-Xa and Activated Partial Thromboplastin Time Monitoring of Heparin in Adult Patients Receiving Extracorporeal Membrane Oxygenation Support	Non-comparative study
**Atallah S, et al[15]**	Evaluation of the activated clotting time and activated partial thromboplastin time for the monitoring of heparin in adult extracorporeal membrane oxygenation patients	Wrong research question

**Aubron C, et al[16]**	Predictive factors of bleeding events in adults undergoing extracorporeal membrane oxygenation	Non-comparative study
**Aubron C et al[17]**	Low-dose heparin versus full systemic anticoagulation in critically ill patients undergoing extracorporeal membrane oxygenation: the HELP-ECMO pilot randomised controlled study	Non-comparative study
**Autschbach T, et al[18]**	Outcomes of extracorporeal membrane oxygenation for acute respiratory distress syndrome in covid-19 patients: A propensity-matched analysis	Wrong study design
**Bakdach D, et al[19]**	Propofol Safety in Anticoagulated and Nonanticoagulated Patients During Extracorporeal Membrane Oxygenation	Insufficient data on anticoagulation
**Bastia L, et al[20]**	Management of venovenous extracorporeal membrane oxygenation with moderate-target anticoagulation: A retrospective study	Insufficient data on anticoagulation
**Bedeir K, et al[21]**	Extracorporeal life support in trauma: Worth the risks? A systematic review of published series	Insufficient data on anticoagulation
**Bemtgen X, et al[22]**	Thrombotic circuit complications during venovenous extracorporeal membrane oxygenation in COVID-19	Insufficient data on anticoagulation
**Biancari F, et al[23]**	Six-Month Survival After Extracorporeal Membrane Oxygenation for Severe COVID-19	Non-comparative study
**Bissell BD, et al[24]**	Evaluation of Bivalirudin as the Primary Anticoagulant in Patients Receiving Extracorporeal Membrane Oxygenation for SARS-CoV-2-Associated Acute Respiratory Failure	Non-comparative study
**Boddi M, et al[25]**	Cannula-related deep vein thrombosis during extracorporeal membrane oxygenation (ECMO) treatment do not affect mortality and length of stay in intensive care unit	Insufficient data on anticoagulation
**Brown MA, et al[26]**	A comparison of bivalirudin and heparin for patients on extracorporeal membrane oxygenation	Wrong study design
**Buchtele N, et al[27]**	Add-On Prostaglandin E1 in Venovenous Extracorporeal Membrane Oxygenation: A Randomized, Double-Blind, Placebo-controlled Pilot Trial	Non-comparative study
**Buscher H, et al[28]**	Blood and Anticoagulation Management in Extracorporeal Membrane Oxygenation for Surgical and Nonsurgical Patients: A Single-Center Retrospective Review	Non-comparative study
**Cahill CM, et al[29]**	Implementation of a Standardized Transfusion Protocol for Cardiac Patients Treated With Venoarterial Extracorporeal Membrane Oxygenation Is Associated With Decreased Blood Component Utilization and May Improve Clinical Outcome	Insufficient data on anticoagulation
**Carter KT, et al[30]**	Heparin-Sparing Anticoagulation Strategies Are Viable Options for Patients on Veno-Venous ECMO	Non-comparative study

**Cartwright B, et al**[31]	Hemostasis, coagulation and thrombin in venoarterial and venovenous extracorporeal membrane oxygenation: the HECTIC study	Non-comparative study
**Cercone JL, et al**[32]	Effect of Initial Anticoagulation Targets on Bleeding and Thrombotic Complications for Patients With Acute Respiratory Distress Syndrome Receiving Extracorporeal Membrane Oxygenation	Non-comparative study
**Chen T-H, et al**[33]	Early percutaneous heparin-free veno-venous extra corporeal life support (ECLS) is a safe and effective means of salvaging hypoxemic patients with complicated chest trauma	Wrong research question
**Chong WH, et al**[34]	Clinical Characteristics Between Survivors and Nonsurvivors of COVID-19 Patients Requiring Extracorporeal Membrane Oxygenation (ECMO) Support: A Systematic Review and Meta-Analysis	Wrong research question
**Chung YS, et al**[35]	Is Stopping Heparin Safe in Patients on Extracorporeal Membrane Oxygenation Treatment?	Insufficient data on anticoagulation
**Cianchi G, et al**[36]	Ventilatory and ECMO treatment of H1N1-induced severe respiratory failure: Results of an Italian referral ECMO center	Non-comparative study
**Ciolek AM, et al**[37]	Use of cangrelor during venoarterial extracorporeal membrane oxygenation following percutaneous coronary intervention	Wrong research question
**Colman E, et al**[38]	Evaluation of a heparin monitoring protocol for extracorporeal membrane oxygenation	Non-comparative study
**Cornell T, et al**[39]	A case series describing the use of argatroban in patients on extracorporeal circulation	Insufficient data on anticoagulation
**Corsi F, et al**[40]	Life-threatening massive pulmonary embolism rescued by venoarterial-extracorporeal membrane oxygenation	Non-comparative study
**Cousin N, et al**[41]	SARS-CoV-2 Versus Influenza-associated Acute Respiratory Distress Syndrome Requiring Veno-venous Extracorporeal Membrane Oxygenation Support	Non-comparative study
**Cunningham D, et al**[42]	Agreement between ACT and aPTT during extracorporeal membrane oxygenation shows intra- and inter-individual variation	Wrong research question
**Deatrick KB, et al**[43]	Pilot study evaluating a non-titrating, weight-based anticoagulation scheme for patients on veno-venous extracorporeal membrane oxygenation	Insufficient data on anticoagulation
**Delmas C, et al**[44]	Anticoagulation Monitoring Under ECMO Support: A Comparative Study Between the Activated Coagulation Time and the Anti-Xa Activity Assay	Wrong research question
**Descamps R, et al**[45]	Anti-Xa activity and hemorrhagic events under extracorporeal membrane oxygenation (ECMO): a multicenter cohort study	ECMO shorter than 24 hours
**Dognon N, et al**[46]	Extracorporeal membrane oxygenation for covid 2019-acute respiratory distress syndrome: Comparison between first and second waves (stage 2)	Non-comparative study

**Doyle AJ, et al**[47]	A Comparison of Thrombosis and Hemorrhage Rates in Patients With Severe Respiratory Failure Due to Coronavirus Disease 2019 and Influenza Requiring Extracorporeal Membrane Oxygenation	Wrong research question
**Durak K, et al**[48]	Extracorporeal membrane oxygenation in patients with COVID-19: 1-year experience	Non-comparative study
**Durak K, et al**[49]	Thromboembolic and Bleeding Events in COVID-19 Patients receiving Extracorporeal Membrane Oxygenation	Data used in other included study
**Esper SA, et al**[50]	Extracorporeal Membrane oxygenation support in acute coronary syndromes complicated by cardiogenic shock	Data used in other included study
**Feih JT, et al**[51]	Heparin Monitoring with an Anti-Xa Protocol Compared to Activated Clotting Time in Patients on Temporary Mechanical Circulatory Support	ECMO shorter than 24 hours
**Fina D, et al**[52]	Extracorporeal membrane oxygenation without therapeutic anticoagulation in adults: A systematic review of the current literature	SR with all studies included
**Fisser C, et al**[53]	[Argatroban in patients with venovenous extracorporeal membrane oxygenation]	Data used in other included study
**Fisser C, et al**[54]	Arterial and venous vascular complications in patients requiring peripheral venoarterial extracorporeal membrane oxygenation	Wrong research question
**Fisser C, et al**[55]	Incidence and risk factors of venous thrombosis after venovenous extracorporeal membrane oxygenation in patients with acute respiratory distress syndrome: A prospective observational study	Insufficient data on anticoagulation
**Fitousis K, et al**[56]	Evaluation of a pharmacy managed heparin protocol for extracorporeal membrane oxygenation patients	Non-comparative study
**Fletcher-Sandersjöö A, et al**[57]	Incidence, outcome, and predictors of intracranial hemorrhage in adult patients on extracorporeal membrane oxygenation: A systematic and narrative review	SR with all studies included
**Fletcher-Sandersjöö A, et al**[58]	Management of intracranial hemorrhage in adult patients on extracorporeal membrane oxygenation (ECMO): An observational cohort study	Non-comparative study
**Fong KM, et al**[59]	Bleeding, thrombosis and transfusion in patients on ECMO: A retrospective study in a tertiary center in Hong Kong	Non-comparative study
**Geli J, et al**[60]	Argatroban Anticoagulation for Adult Extracorporeal Membrane Oxygenation: A Systematic Review	SR with all studies included
**Giuliano K, et al**[61]	Extracorporeal Membrane Oxygenation Complications in Heparin- and Bivalirudin-Treated Patients	Non-comparative study
**Glick D, et al**[62]	Clinically suspected heparin-induced thrombocytopenia during extracorporeal membrane oxygenation	Non-comparative study

**Guliani S, et al**[63]	Venoarterial extracorporeal membrane oxygenation is an effective management strategy for massive pulmonary embolism patients	Non-comparative study
**Guo Z, et al**[64]	Anticoagulation Management in Severe Coronavirus Disease 2019 Patients on Extracorporeal Membrane Oxygenation	Non-comparative study
**Han W, et al**[65]	Single-center experience of extracorporeal membrane oxygenation mainly anticoagulated with nafamostat mesilate	Pediatric patients
**Han X, et al**[66]	Mechanical Circulatory Support in Cardiovascular Surgical Patients: Single Center Practice and Experience	Wrong study design
**Hanna DJ, et al**[67]	Evaluation of anticoagulation with bivalirudin for heparin-induced thrombocytopenia during extracorporeal membrane oxygenation	Insufficient data on anticoagulation
**Harahwa T, et al**[68]	The use of extracorporeal membrane oxygenation postcardiotomy—A systematic review	SR with all studies included
**Hartley EL, et al**[69]	Screening pulmonary angiogram and the effect on anticoagulation strategies in severe respiratory failure patients on venovenous extracorporeal membrane oxygenation	Wrong study design
**Hasegawa D, et al**[70]	Comparison of Bivalirudin Versus Heparin for Anticoagulation During Extracorporeal Membrane Oxygenation	SR with all studies included
**Hékimian G, et al**[71]	Extracorporeal Membrane Oxygenation Induces Early Alterations in Coagulation and Fibrinolysis Profiles in COVID-19 Patients with Acute Respiratory Distress Syndrome	Non-comparative study
**Hemmila MR, et al**[72]	Extracorporeal Life Support for Severe Acute Respiratory Distress Syndrome in Adults	Non-comparative study
**Hermann A, et al**[73]	ECMO without anticoagulation in patients with disease-related severe thrombocytopenia: Feasible but futile?	Insufficient data on anticoagulation
**Hohlfelder B, et al**[74]	Activated Clotting Times Demonstrate Weak Correlation With Heparin Dosing in Adult Extracorporeal Membrane Oxygenation	Insufficient data on anticoagulation
**Holzer F, et al**[75]	Predictors of bleeding in ECMO patients undergoing surgery	Insufficient data on anticoagulation
**Hong JI, et al**[76]	Satisfactory outcome with low activated clotting time in extracorporeal membrane oxygenation	Insufficient data on anticoagulation
**Hou D, et al**[77]	Neurologic Complications in Adult Post-cardiotomy Cardiogenic Shock Patients Receiving Venoarterial Extracorporeal Membrane Oxygenation: A Cohort Study	Non-comparative study
**Hou X, et al**[78]	Extracorporeal Membrane Oxygenation for Critically Ill Patients With 2009 Influenza A (H1N1)-Related Acute Respiratory Distress Syndrome: Preliminary Experience From a Single Center	Non-comparative study

**Hu S, et al**[79]	Limb Ischemia Complications of Veno-Arterial Extracorporeal Membrane Oxygenation	Non-comparative study
**Huang D, et al**[80]	Bivalirudin versus heparin anticoagulation in patients receiving extracorporeal membrane oxygenation	SR with all studies included
**Huang W, et al**[81]	Outcomes from the use of perioperative extracorporeal membrane oxygenation in patients undergoing thoracic surgery: An 8-year single-center experience	Non-comparative study
**Huprikar NA, et al**[82]	Salvage extracorporeal membrane oxygenation in induction-associated acute respiratory distress syndrome in acute leukemia patients: A case series	Wrong study design
**Ius F, et al**[83]	Veno-veno-arterial extracorporeal membrane oxygenation for respiratory failure with severe haemodynamic impairment: Technique and early outcomes	Insufficient data on anticoagulation
**Jacobs JP, et al**[84]	Multi-institutional Analysis of 200 COVID-19 Patients Treated With Extracorporeal Membrane Oxygenation: Outcomes and Trends	Insufficient data on anticoagulation
**Jang J, et al**[85]	Clinical Experiences of High-Risk Pulmonary Thromboembolism Receiving Extracorporeal Membrane Oxygenation in Single Institution	Non-comparative study
**Jin Y, et al**[86]	Ischemic stroke and intracranial hemorrhage in extracorporeal membrane oxygenation for COVID-19: A systematic review and meta-analysis	SR with all studies included
**Jiritano F, et al**[87]	Systematic review and meta-analysis of the clinical effectiveness of point-of-care testing for anticoagulation management during ECMO	SR with all studies included
**Jiritano F, et al**[88]	Platelets and extra-corporeal membrane oxygenation in adult patients: a systematic review and meta-analysis	SR with all studies included
**Kakar V, et al**[89]	Long runs and higher incidence of bleeding complications in covid-19 patients requiring venovenous extracorporeal membrane oxygenation: A case series from the united arab emirates	Non-comparative study
**Kalbhenn J, et al**[90]	Hypercoagulopathy, acquired coagulation disorders and anticoagulation before, during and after extracorporeal membrane oxygenation in COVID-19: a case series	Non-comparative study
**Kannapadi NV, et al**[91]	Neurological Complications in COVID-19 Patients With ECMO Support: A Systematic Review and Meta-Analysis	SR with all studies included
**Kannapadi NV, et al**[92]	Neurologic Injury in Patients With COVID-19 Who Receive VV-ECMO Therapy: A Cohort Study	Wrong study design
**Karim AS, et al**[93]	Pre-Intubation Veno-Venous Extracorporeal Membrane Oxygenation in Patients at Risk for Respiratory Decompensation	ECMO shorter than 24 hours
**Karampinis I, et al**[94]	Thoracic surgery in patients on veno-venous extracorporeal membrane oxygenation for COVID-19 associated acute respiratory distress syndrome	Non-comparative study
**Kim BK, et al**[95]	Satisfactory outcome with activated clotting time <210 seconds in extracorporeal cardiopulmonary resuscitation	Data used in other included study

**Kim YS, et al**[96]	Use of argatroban for extracorporeal life support in patients with nonheparin-induced thrombocytopenia: Analysis of 10 consecutive patients	Insufficient data on anticoagulation
**Klinzing S, et al**[97]	Neurologic Injury with Severe Adult Respiratory Distress Syndrome in Patients Undergoing Extracorporeal Membrane Oxygenation: A Single-Center Retrospective Analysis	Wrong research question
**Kohs TCL, et al**[98]	Severe thrombocytopenia in adults undergoing extracorporeal membrane oxygenation is predictive of thrombosis	Non-comparative study
**Kolla S, et al**[99]	Extracorporeal life support for cardiovascular support in adults	Data used in other included study
**Kolla S, et al**[100]	Extracorporeal life support for 100 adult patients with severe respiratory failure	Non-comparative study
**Kon ZN, et al**[101]	Extracorporeal Membrane Oxygenation Support in Severe COVID-19	Non-comparative study
**Kreyer S, et al**[102]	Hemorrhage under veno-venous extracorporeal membrane oxygenation in acute respiratory distress syndrome patients: A retrospective data analysis	Non-comparative study
**Krueger K, et al**[103]	Venovenous Extracorporeal Membrane Oxygenation With Prophylactic Subcutaneous Anticoagulation Only: An Observational Study in More Than 60 Patients	ECMO shorter than 24 hours
**Kucuk AO, et al**[104]	Extracorporeal membrane oxygenation experiences during COVID-19 pandemic, third wave with younger patients: A retrospective observational study	Non-comparative study
**Kunavarapu C, et al**[105]	Clinical outcomes of severe COVID-19 patients receiving early VV-ECMO and the impact of pre-ECMO ventilator use	Non-comparative study
**Kurihara C et al**[106]	Feasibility of Venovenous Extracorporeal Membrane Oxygenation Without Systemic Anticoagulation	Non-comparative study
**Kutleša M, et al**[107]	Venovenous extracorporeal membrane oxygenation for ARDS: outcome analysis of a Croatian referral center for respiratory ECMO	Non-comparative study
**La Via L, et al**[108]	Use of nafamostat mesilate for anticoagulation during extracorporeal membrane oxygenation: A systematic review	Wrong study design
**Lai C-H, et al**[109]	Percutaneous coronary intervention under extracorporeal membrane oxygenation support for high-risk acute myocardial infarction with cardiogenic shock	Insufficient data on anticoagulation
**Lai W, et al**[110]	Severe Patients With ARDS With COVID-19 Treated With Extracorporeal Membrane Oxygenation in China: A Retrospective Study	Non-comparative study
**Laimoud M, et al**[111]	Acute neurological complications in adult patients with cardiogenic shock on veno-arterial extracorporeal membrane oxygenation support	Data used in other included study

**Laimoud M, et al**[112]	Acute vascular complications of femoral veno-arterial ECMO: a single-centre retrospective study	Data used in other included study
**Laimoud M, et al**[113]	Hospital outcomes after emergent peripheral veno-arterial extracorporeal membrane oxygenation in adult patients presenting with cardiogenic shock	Non-comparative study
**Laine A, et al**[114]	Decreased maximum clot firmness in rotational thromboelastometry (ROTEM R) is associated with bleeding during extracorporeal mechanical circulatory support	Non-comparative study
**Lamarche Y, et al**[115]	Thromboembolic Events in Patients on Extracorporeal Membrane Oxygenation without Anticoagulation	Non-comparative study
**Lang CN, et al**[116]	Intracerebral Hemorrhage in COVID-9 Patients with Pulmonary Failure: A Propensity Score-Matched Registry Study	Insufficient data on anticoagulation
**Lankford AS, et al**[117]	Clinical Outcomes of Pregnant and Postpartum Extracorporeal Membrane Oxygenation Patients	Non-comparative study
**Li D-H, et al**[118]	Is bivalirudin an alternative anticoagulant for extracorporeal membrane oxygenation (ECMO) patients? A systematic review and meta-analysis	SR with all studies included
**Li M-J, et al**[119]	Bivalirudin versus heparin in adult and pediatric patients with extracorporeal membrane oxygenation therapy: A systematic review and meta-analysis	SR with all studies included
**Lin T-W, et al**[120]	Simultaneous Thrombolysis and Extracorporeal Membrane Oxygenation for Acute Massive Pulmonary Emboli	Non-comparative study
**Liu L, et al**[121]	Bivalirudin versus heparin in adult and pediatric patients with extracorporeal membrane oxygenation therapy: A systematic review and meta-analysis	SR with all studies included
**Liu Y, et al**[122]	A Comparison of Activated Partial Thromboplastin Time and Activated Coagulation Time for Anticoagulation Monitoring during Extracorporeal Membrane Oxygenation Therapy	Non-comparative study
**Liu Z, et al**[123]	Extracorporeal Membrane Oxygenation—First Strategy for Acute Life-Threatening Pulmonary Embolism	Non-comparative study
**Loforte A, et al**[124]	Extracorporeal Membrane Oxygenation Support in Refractory Cardiogenic Shock: Treatment Strategies and Analysis of Risk Factors	Insufficient data on anticoagulation
**López Sánchez M, et al**[125]	Mechanical complications of extracorporeal membrane oxygenation	Wrong study design
**Lubnow M, et al**[126]	Prevalence and outcomes of patients developing heparin-induced thrombocytopenia during extracorporeal membrane oxygenation	Non-comparative study
**Lv X, et al**[127]	Low vs standardized dose anticoagulation regimens for extracorporeal membrane oxygenation: A meta-analysis	SR with all studies included

**M'Pembele R, et al**[128]	Evaluation of clinical outcomes in patients treated with heparin or direct thrombin inhibitors during extracorporeal membrane oxygenation: a systematic review and meta-analysis	SR with all studies included
**Ma M, et al**[129]	The Efficacy and Safety of Bivalirudin Versus Heparin in the Anticoagulation Therapy of Extracorporeal Membrane Oxygenation: A Systematic Review and Meta-Analysis	SR with all studies included
**Macielak S, et al**[130]	Impact of anticoagulation strategy and agents on extracorporeal membrane oxygenation therapy	Insufficient data on anticoagulation
**Martucci G, et al**[131]	Anticoagulation and Transfusions Management in Veno-Venous Extracorporeal Membrane Oxygenation for Acute Respiratory Distress Syndrome: Assessment of Factors Associated With Transfusion Requirements and Mortality	Non-comparative study
**Martucci G, et al**[132]	The role of endoscopy as non-invasive procedure to manage gastrointestinal complications during extracorporeal membrane oxygenation	Data used in other included study
**Mazzeffi MA, et al**[133]	Bleeding, Thrombosis, and Transfusion With Two Heparin Anticoagulation Protocols in Venoarterial ECMO Patients	Non-comparative study
**Mazzeffi M, et al**[134]	Bleeding, Transfusion, and Mortality on Extracorporeal Life Support: ECLS Working Group on Thrombosis and Hemostasis	Data used in other included study
**McQuilten Z, et al**[135]	Low-dose heparin in critically Ill patients undergoing extracorporeal membrane oxygenation-the help-ECMO pilot randomised controlled trial	Data used in other included study
**Mégarbane B, et al**[136]	Coagulation status in poisonings requiring extracorporeal life support in relation to refractory cardiac failure or arrest: A prospective study	Wrong research question
**Melehy A, et al**[137]	Prosthetic valve thrombosis during extracorporeal life support for postcardiotomy shock	Wrong study design
**Menk M, et al**[138]	Efficacy and safety of argatroban in patients with acute respiratory distress syndrome (ARDS) and extracorporeal membrane oxygenation (ECMO)	Insufficient data on anticoagulation
**Mihu MR, et al**[139]	Veno-arteriovenous extracorporeal membrane oxygenation—A single center experience	Wrong study design
**Moussa MD, et al**[140]	Evaluation of anti-activated factor x activity and activated partial thromboplastin time relations and their association with bleeding and thrombosis during veno-arterial ecmo support: A retrospective study	Non-comparative study
**Murakami T, et al**[141]	Comparison of medical resource use and total admission cost in patients with acute myocardial infarction between on-hours visit versus off-hours visit	Non-comparative study
**Netley J, et al**[142]	Bivalirudin anticoagulation dosing protocol for extracorporeal membrane oxygenation: A retrospective review	Non-comparative study
**Ng GWY, et al**[143]	Three-year experience of using venovenous extracorporeal membrane oxygenation for patients with severe respiratory failure	Non-comparative study

**Northam KA, et al[144]**	Evaluation of a Multimodal Heparin Laboratory Monitoring Protocol in Adult Extracorporeal Membrane Oxygenation Patients	Data used in other included study
**Northam KA, et al[145]**	Major Bleeding and Thrombosis Events in COVID-19 Versus Influenza Patients Requiring Extracorporeal Membrane Oxygenation	Non-comparative study
**O'Malley TJ, et al[146]**	Outcomes of extracorporeal life support for the treatment of acute massive pulmonary embolism: A systematic review	SR with all studies included
**Ogami T, et al[147]**	A Standardized Approach Improves Outcomes of Extracorporeal Membrane Oxygenation for Postcardiotomy Shock	Insufficient data on anticoagulation
**Olson SR, et al[148]**	Thrombosis and Bleeding in Extracorporeal Membrane Oxygenation (ECMO) without Anticoagulation: A Systematic Review	SR with all studies included
**Oude Lansink-Hartgring A, et al[149]**	Hemorrhagic complications during extracorporeal membrane oxygenation – The role of anticoagulation and platelets	Non-comparative study
**Pabst D, et al[150]**	Heparin-induced thrombocytopenia in patients on extracorporeal membrane oxygenation and the role of a heparin-bonded circuit	Non-comparative study
**Panigada M, et al[151]**	Thromboelastography-based anticoagulation management during extracorporeal membrane oxygenation: a safety and feasibility pilot study	Non-comparative study
**Papadopoulos N, et al[152]**	Risk factors associated with adverse outcome following extracorporeal life support: Analysis from 360 consecutive patients	Non-comparative study
**Parker BM, et al[153]**	Single Center Experience With Veno-Venous Extracorporeal Membrane Oxygenation in Patients With Traumatic Brain Injury	Insufficient data on anticoagulation
**Pieri M, et al[154]**	Bivalirudin versus heparin as an anticoagulant during extracorporeal membrane oxygenation: A case-control study	Wrong research question
**Popugaev KA, et al[155]**	Clinical and pathophysiologic aspects of ECMO-associated hemorrhagic complications	Non-comparative study
**Pozzi M, et al[156]**	Veno-arterial extracorporeal membrane oxygenation for drug intoxications: A single center, 14-year experience	Non-comparative study
**Pranikoff T, et al[157]**	Efficacy of extracorporeal life support in the setting of adult cardiorespiratory failure	Non-comparative study
**Reddy HG, et al[158]**	Utilization of extracorporeal life support for diffuse alveolar damage and diffuse alveolar hemorrhage: A systematic review	SR with all studies included
**Renaudier M, et al[159]**	Acute mesenteric ischaemia in refractory shock on veno-arterial extracorporeal membrane oxygenation	Non-comparative study
**Rhoades R, et al[160]**	Coagulopathy monitoring and anticoagulation management in COVID-19 patients on ECMO: Advantages of a heparin anti-Xa-based titration strategy	Wrong study design
**Ried M, et al[161]**	Thoracic Bleeding Complications in Patients With Venovenous Extracorporeal Membrane Oxygenation	Data used in other included study

**Riera J, et al**[162]	Extracorporeal Membrane Oxygenation Retrieval in Coronavirus Disease 2019: A Case-Series of 19 Patients Supported at a High-Volume Extracorporeal Membrane Oxygenation Center	Non-comparative study
**Ripoll B, et al**[163]	Observational study of thrombosis and bleeding in COVID-19 VV ECMO patients	Wrong study design
**Roedl K, et al**[164]	Clinical characteristics, complications and outcomes of patients with severe acute respiratory distress syndrome related to covid-19 or influenza requiring extracorporeal membrane oxygenation—a retrospective cohort study	Non-comparative study
**Sadeghipour P, et al**[165]	Effect of Intermediate-Dose vs Standard-Dose Prophylactic Anticoagulation on Thrombotic Events, Extracorporeal Membrane Oxygenation Treatment, or Mortality among Patients with COVID-19 Admitted to the Intensive Care Unit: The INSPIRATION Randomized Clinical Trial	Wrong research question
**Sanfilippo F, et al**[166]	Bivalirudin for Alternative Anticoagulation in Extracorporeal Membrane Oxygenation: A Systematic Review	SR with all studies included
**Sanfilippo F, et al**[167]	Use of nafamostat mesilate for anticoagulation during extracorporeal membrane oxygenation: A systematic review	SR with all studies included
**Seeliger B, et al**[168]	Comparison of anticoagulation strategies for veno-venous ECMO support in acute respiratory failure: A retrospective comparative cohort study	Non-comparative study
**Seeliger B, et al**[169]	Intracranial Hemorrhages on Extracorporeal Membrane Oxygenation: Differences Between COVID-19 and Other Viral Acute Respiratory Distress Syndrome	Data used in other included study
**Shah A, et al**[170]	A Comparison of Anticoagulation Strategies in Veno-venous Extracorporeal Membrane Oxygenation	Insufficient data on anticoagulation
**Shih E, et al**[171]	Venovenous extracorporeal membrane oxygenation for patients with refractory coronavirus disease 2019 (COVID-19): Multicenter experience of referral hospitals in a large health care system	Non-comparative study
**Siatan JE, et al**[172]	Incidence of clinically significant bleeding and thromboembolism among patients with leptospirosis requiring extra corporeal membrane oxygenation: A descriptive study	Wrong study design
**Sklar MC, et al**[173]	Anticoagulation practices during venovenous extracorporeal membrane oxygenation for respiratory failure a systematic review	SR with all studies included
**Stammers AH, et al**[174]	Variation in outcomes with extracorporeal membrane oxygenation in the era of coronavirus: A multicenter cohort evaluation	Insufficient data on anticoagulation

**Staudacher DL, et al[175]**	Dual Antiplatelet Therapy (DAPT) versus no antiplatelet therapy and incidence of major bleeding in patients on venoarterial extracorporeal membrane oxygenation	Non-comparative study
**Stokes JW, et al[176]**	Bleeding, Thromboembolism, and Clinical Outcomes in Venovenous Extracorporeal Membrane Oxygenation	Wrong study design
**Stub D, et al[177]**	Refractory cardiac arrest treated with mechanical CPR, hypothermia, ECMO and early reperfusion (the CHEER trial)	Insufficient data on anticoagulation
**Sutter R, et al[178]**	Acute neurologic complications during extracorporeal membrane oxygenation: A systematic review	SR with all studies included
**Sy E, et al[179]**	Anticoagulation practices and the prevalence of major bleeding, thromboembolic events, and mortality in venoarterial extracorporeal membrane oxygenation: A systematic review and meta-analysis	SR with all studies included
**Trigonis R, et al[180]**	Efficacy of Bivalirudin for Therapeutic Anticoagulation in COVID-19 Patients Requiring ECMO Support	Non-comparative study
**Tsai M-T, et al[181]**	Bridge-to-recovery strategy using extracorporeal membrane oxygenation for critical pulmonary hypertension complicated with cardiogenic shock	Non-comparative study
**Umei N, et al[182]**	Early predictors of oxygenator exchange during veno-venous extracorporeal membrane oxygenation: A retrospective analysis	Non-comparative study
**Valente S, et al[183]**	Bleeding events in refractory cardiac arrest treated with extracorporeal membrane oxygenation - A single centre experience	Wrong study design
**Walker EA, et al[184]**	Bivalirudin dosing requirements in adult patients on extracorporeal life support with or without continuous renal replacement therapy	Wrong research question
**Wang C, et al[185]**	Extracorporeal membrane oxygenation in trauma patients: a systematic review	SR with all studies included
**Weatherill A, et al[186]**	Impact of Thrombosis and Bleeding in Patients with Severe COVID-19 versus Other Viral Pneumonias in the Context of Extracorporeal Membrane Oxygenation	Wrong study design
**Weber C, et al[187]**	Left ventricular thrombus formation in patients undergoing femoral veno-arterial extracorporeal membrane oxygenation	Non-comparative study
**Weingart C, et al[188]**	Comparison of Coagulation Parameters, Anticoagulation, and Need for Transfusion in Patients on Interventional Lung Assist or Veno-Venous Extracorporeal Membrane Oxygenation	Data used in other included study
**Weir-Mccall JR, et al[189]**	Thrombosis in Severe COVID-19 Requiring Extracorporeal Membrane Oxygenation: A Multicenter Study	Non-comparative study
**Wilcox C, et al[190]**	Acute Brain Injury in Postcardiotomy Shock Treated With Venoarterial Extracorporeal Membrane Oxygenation	Non-comparative study

**Willems A, et al**[191]	Anti-Xa versus time-guided anticoagulation strategies in extracorporeal membrane oxygenation: a systematic review and meta-analysis	SR with all studies included
**Willers A, et al**[192]	Extracorporeal Life Support in Hemorrhagic Conditions: A Systematic Review	SR with all studies included
**Wohlfarth P, et al**[193]	Characteristics and outcome of patients after allogeneic hematopoietic stem cell transplantation treated with extracorporeal membrane oxygenation for acute respiratory distress syndrome	Insufficient data on anticoagulation
**Wood KL, et al**[194]	Venoarterial-Extracorporeal Membrane Oxygenation Without Routine Systemic Anticoagulation Decreases Adverse Events	Non-comparative study
**Yajun Q, et al**[195]	Application of extracorporeal membrane oxygenation in critically ill pregnant women	Insufficient data on anticoagulation
**Yasuda N, et al**[196]	A new anticoagulation strategy using recombinant human thrombomodulin in patients on veno-venous extracorporeal membrane oxygenation: a retrospective study	Non-comparative study
**Zhao Y-C, et al**[197]	Heparin-free after 3000 IU heparin loaded in veno-venous ECMO supported acute respiratory failure patients with hemorrhage risk: a novel anti-coagulation strategy	Non-comparative study
**Abramov, et al**[198]	Extracorporeal Membrane Oxygenation Support in COVID-19 Patients: A Propensity Score Analysis	Insufficient data on anticoagulation
**Abruzzo, et al**[199]	Venous thromboembolic events in the setting of extracorporeal membrane oxygenation support in adults: A systematic review	SR with all studies included
**Akkanti, et al**[200]	Extracorporeal Membrane Oxygenation for COVID-19: Collaborative Experience from the Texas Medical Center in Houston with 2 Year Follow-Up	Insufficient data on anticoagulation
**Aldairi, et al**[201]	Efficacy of Prostacyclin Anticoagulation in Critically Ill Patients Requiring Extracorporeal Support: A Systematic Review and Meta-Analysis.	SR with all studies included
**Alves Cabrita, et al**[202]	Anticoagulation in ECMO patients: Management in a portuguese center	Wrong study design
**Arachchillage, et al**[203]	Outcomes in COVID-19 supported by ECMO in the first vs second wave - multicentre observational study	Wrong study design
**Araújo Silva, et al**[204]	Risk factors for major neurological events in patients under venovenous extracorporeal membrane oxygenation - single centre study	Wrong study design
**Austin, et al**[205]	Veno-venous extracorporeal membrane oxygenation should be considered early in patients with traumatic brain injuries and severe respiratory failure	Wrong study design
**Baldetti, et al**[206]	Anti-thrombotic Therapy With Cangrelor and Bivalirudin in Venoarterial Extracorporeal Membrane Oxygenation Patients Undergoing Percutaneous Coronary Intervention: A Single-Center Experience	Wrong study design

**Baumgartner, et al**[207]	Major Bleeding and Thromboembolic Events in Veno-Venous Extracorporeal Membrane Oxygenation-Patients with Isolated Respiratory Failure	Pediatric patients
**Bento Rodrigues, et al**[208]	Hemorrhagic and thrombotic complications under VV-ECMO in patients with SARS-CoV-2 infection: learnings from a single center	Wrong study design
**Bermea, et al**[209]	Increased Intracranial Hemorrhage Amid Elevated Inflammatory Markers in Those With COVID-19 Supported With Extracorporeal Membrane Oxygenation	Insufficient data on anticoagulation
**Bernal, et al**[210]	THROMBOTIC COMPLICATIONS OF EXTRA-CORPOREAL CARDIOPULMONARY RESUSCITATION FOR VT/VF OUT OF HOSPITAL CARDIAC ARREST AND ASSOCIATED MORTALITY	Wrong study design
**Brasil, et al**[211]	Veno-venous extracorporeal membrane oxygenation in patients with SARS-CoV-2 pneumonia in Brazil: a case series	Insufficient data on anticoagulation
**Buckley, et al**[212]	CORRELATION BETWEEN PTT AND THROMBOELASTOGRAPHY IN ADULT ICU PATIENTS REQUIRING BIVALIRUDIN FOR ECMO	Wrong study design
**Buckley, et al**[213]	Comparison of Fixed-Dose Inhaled Epoprostenol and Inhaled Nitric Oxide for Acute Respiratory Distress Syndrome in Critically Ill Adults	Wrong study design
**Chang, et al**[214]	The safety of discontinuing heparin in patients with venoarterial ECMO	Wrong study design
**Chang, et al**[215]	INCIDENCE AND RISK FACTORS ASSOCIATED WITH BLEEDING IN ADULTS UNDERGOING EXTRACORPOREAL CARDIOPULMONARY RESUSCITATION FOR VT/VF OUT OF HOSPITAL CARDIAC ARREST	Wrong study design
**Chen, et al**[216]	Anticoagulation strategies in patients with extracorporeal membrane oxygenation: A network meta-analysis and systematic review	SR with all studies included
**Cho, et al**[217]	Ischemic and Hemorrhagic Stroke Among Critically Ill Patients With Coronavirus Disease 2019: An International Multicenter Coronavirus Disease 2019 Critical Care Consortium Study	Insufficient data on anticoagulation
**Cohan, et al**[218]	Comparison of clinical outcomes with cangrelor plus aspirin versus oral dual antiplatelet therapy in patients supported with venoarterial extracorporeal membrane oxygenation	Wrong study design
**De Paulis, et al**[219]	Anticoagulation Management in High Bleeding-Risk ECMO in Adults	Wrong study design
**Diaz, et al**[220]	Anticoagulation strategies in COVID-19 infected patients receiving ECMO support	Wrong study design
**El Banayosy AM, et al**[221]	Extracorporeal life support in pregnant and postpartum women with COVID-19-related acute respiratory distress syndrome	Non-comparative study
**Fisser, et al**[222]	Arterial and venous vascular complications in patients requiring peripheral venoarterial extracorporeal membrane oxygenation	Data used in other included study
**Golicnik A, et al**[223]	Same but Different—ECMO in COVID-19 and ARDS of Other Etiologies. Comparison of Survival Outcomes and Management in Different ARDS Groups	Non-comparative study

**Gu, et al**[224]	Superiority of bivalirudin over heparin anticoagulation therapy for extracorporeal membrane oxygenation? Too early to draw conclusions.	SR with all studies included
**Hanna, et al**[225]	EVALUATION OF BIVALIRUDIN ANTICOAGULATION IN PATIENTS ON ECMO DUE TO COVID-19 RESPIRATORY FAILURE	Wrong study design
**Hasegawa, et al**[226]	Comparison of Bivalirudin Versus Heparin for Anticoagulation during Extracorporeal Membrane Oxygenation	SR with all studies included
**Hofmaenner, et al**[227]	Reduced anticoagulation strategy is associated with a lower incidence of intracerebral hemorrhage in COVID-19 patients on extracorporeal membrane oxygenation	Data used in other included study
**Honda, et al**[228]	Risk factors for severe lower extremity ischemia following venoarterial extracorporeal membrane oxygenation: An analysis using a nationwide inpatient database	Insufficient data on anticoagulation
**Huang, et al**[229]	Long-term outcomes of high-risk percutaneous coronary interventions under extracorporeal membrane oxygenation support: An observational study	Insufficient data on anticoagulation
**Iannattone, et al**[230]	Incidence of Venous Thromboembolism in Adults Receiving Extracorporeal Membrane Oxygenation: A Systematic Review	SR with all studies included
**Jacobs, et al**[231]	Variation in survival in patients with Coronavirus Disease 2019 supported with extracorporeal membrane oxygenation: A multi-institutional analysis of 590 consecutive patients with Coronavirus 2019 with extracorporeal membrane oxygenation at 59 hospitals within 21 states	Insufficient data on anticoagulation
**Jang, et al**[232]	Clinical experiences of high-risk pulmonary thromboembolism receiving extracorporeal membrane oxygenation in single institution	Insufficient data on anticoagulation
**Jin, et al**[233]	Thrombosis and bleeding in patients with COVID-19 requiring extracorporeal membrane oxygenation: a systematic review and meta-analysis	SR with all studies included
**Jones, et al**[234]	Thromboembolism in COVID-19 patients on ECMO	Wrong study design
**Kawaji, et al**[235]	Bivalirudin and Venoarterial Extracorporeal Membrane Oxygenation for Treatment of Massive Pulmonary Embolism From Heparin-Induced Thrombocytopenia With Thrombosis.	Wrong study design
**Kido, et al**[236]	Meta-Analysis Comparing Bivalirudin Versus. Unfractionated Heparin in Adult Patients With Extracorporeal Membrane Oxygenation	SR with all studies included
**Kim, et al**[237]	Outcomes in trauma patients undergoing veno-venous extracorporeal membrane oxygenation for acute respiratory distress syndrome	Insufficient data on anticoagulation
**Kutleša, et al**[238]	Extracorporeal membrane oxygenation in COVID-19: Results of the Croatian Extracorporeal Membrane Oxygenation Referral Center	Wrong research question
**Lankford, et al**[239]	Clinical Outcomes of Pregnant and Postpartum Extracorporeal Membrane Oxygenation Patients	Data used in other included study

**Lannon, et al[240]**	Intracranial hemorrhage in patients treated for SARS-CoV-2 with extracorporeal membrane oxygenation: A systematic review and meta-analysis	SR with all studies included
**Lejeune, et al[241]**	Longitudinal measurement of whole blood viscoelastic properties throughout the course of extracorporeal membrane oxygenation in COVID-19 patients with severe acute respiratory distress syndrome	Insufficient data on anticoagulation
**Li, et al[242]**	Is bivalirudin an alternative anticoagulant for extracorporeal membrane oxygenation (ECMO) patients? A systematic review and meta-analysis	SR with all studies included
**Lopez, et al[243]**	Evaluation of Bivalirudin During Adult Extracorporeal Membrane Oxygenation: A Retrospective Characterization of Dosing, Efficacy and Bleeding	Insufficient data on anticoagulation
**Lubnow, et al[244]**	Prevalence and outcomes of patients developing heparin-induced thrombocytopenia during extracorporeal membrane oxygenation	Data used in other included study
**Lüsebrink E, et al[245]**	Heparin-Induced Thrombocytopenia in Patients Undergoing Venoarterial Extracorporeal Membrane Oxygenation	Non-comparative study
**Ma, et al[246]**	The Efficacy and Safety of Bivalirudin Versus Heparin in the Anticoagulation Therapy of Extracorporeal Membrane Oxygenation: A Systematic Review and Meta-Analysis	SR with all studies included
**Marquardt, et al[247]**	BIVALIRUDIN VERSUS HEPARIN FOR ANTICOAGULATION IN EXTRACORPOREAL MEMBRANE OXYGENATION	Wrong study design
**Martucci, et al[248]**	Bleeding during venovenous extracorporeal membrane oxygenation. Preliminary data from the PROTECMO study	Wrong study design
**Mazzeffi, et al[249]**	Outcomes of heparinized adult veno-arterial extracorporeal membrane oxygenation patients managed with low and high activated partial thromboplastin time targets: A systematic review and meta-analysis	SR with all studies included
**Navaei, et al[250]**	Is it time to switch to bivalirudin for ECMO anticoagulation?	SR with all studies included
**Nepomuceno, et al[251]**	Anticoagulation strategies and outcomes during extracorporeal membrane oxygenation support: a single-centre pre-COVID-19 experience	Wrong study design
**Netley, et al[252]**	Bivalirudin anticoagulation dosing protocol for extracorporeal membrane oxygenation: A retrospective review	Data used in other included study
**Pang, et al[253]**	Effects and safety of extracorporeal membrane oxygenation in the treatment of patients with ST-segment elevation myocardial infarction and cardiogenic shock: A systematic review and meta-analysis	SR with all studies included
**Piwowarczyk P, et al[254]**	Challenges and pitfalls of extracorporeal membrane oxygenation in critically-ill pregnant and peripartum women with COVID-19: a retrospective case series	Non-comparative study
**Powell, et al[255]**	Early veno-venous extracorporeal membrane oxygenation is an effective strategy for traumatically injured patients presenting with refractory respiratory failure	Wrong research question

**Rajsic, et al**[256]	The Role of Excessive Anticoagulation and Missing Hyperinflammation in ECMO-Associated Bleeding	Wrong research question
**Rajsic, et al**[257]	Association of aPTT-Guided Anticoagulation Monitoring with Thromboembolic Events in Patients Receiving V-A ECMO Support: A Systematic Review and Meta-Analysis	SR with all studies included
**Rajsic, et al**[258]	Extracorporeal membrane oxygenation for cardiogenic shock: a meta-analysis of mortality and complications	SR with all studies included
**Rajsic, et al**[259]	aPTT-guided anticoagulation monitoring during ECMO support: A systematic review and meta-analysis	SR with all studies included
**Rajsic, et al**[260]	Thrombotic events develop in 1 out of 5 patients during ECMO support: An 11-year referral center experience	Wrong study design
**Renaudier, et al**[261]	Acute mesenteric ischaemia in refractory shock on veno-arterial extracorporeal membrane oxygenation	Data used in other included study
**Saeed, et al**[262]	Outcomes with Direct and Indirect Thrombin Inhibition during Extracorporeal Membrane Oxygenation for COVID-19	Data used in other included study
**Saeed, et al**[263]	Characteristics and outcomes of patients with COVID-19 supported by extracorporeal membrane oxygenation: A retrospective multicenter study	Insufficient data on anticoagulation
**Sanfilippo, et al**[264]	Use of nafamostat mesilate for anticoagulation during extracorporeal membrane oxygenation: A systematic review	SR with all studies included
**Sanfilippo, et al**[265]	More evidence available for the use of Bivalirudin in patients supported by extracorporeal membrane oxygenation	Wrong study design
**Schaefer, et al**[266]	Bleeding and thrombotic events in post-cardiotomy extracorporeal life support	ECMO shorter than 24 hours
**Shah A, et al**[267]	A Comparison of Anticoagulation Strategies in Veno-venous Extracorporeal Membrane Oxygenation	Non-comparative study
**Slampak-Cindric, et al**[268]	ANTICOAGULATION IN ADULT ECMO: PERCENTAGE OF TIME IN THERAPEUTIC RANGE WITH HEPARIN OR BIVALIRUDIN	Wrong study design
**Stammers, et al**[269]	Anticoagulant Use During Extracorporeal Membrane Oxygenation Using Heparin and Direct Thrombin Inhibitors in COVID-19 and ARDS Patients	Insufficient data on anticoagulation
**Stern J, et al**[270]	Upper gastrointestinal bleeding in adults treated with veno-arterial extracorporeal membrane oxygenation: a cohort study	Non-comparative study
**Sun, et al**[271]	Comparison of anticoagulation monitoring strategies for adults supported on extracorporeal membrane oxygenation: A systematic review	SR with all studies included
**Uricchio, et al**[272]	Bivalirudin Versus Unfractionated Heparin in Patients With Cardiogenic Shock Requiring Venoarterial Extracorporeal Membrane Oxygenation	Data used in other included study
**Vale, et al**[273]	Bivalirudin during extracorporeal support - a retrospective case series	Wrong study design

**Widmeier, et al[274]**	Extracorporeal membrane oxygenation during the first three waves of the coronavirus disease 2019 pandemic: A retrospective single-center registry study	Wrong research question
**Wieruszewski, et al[275]**	Heparin Versus Bivalirudin for Anticoagulation in Adult Extracorporeal Membrane Oxygenation: A Systematic Review and Meta-Analysis	SR with all studies included
**Willers, et al[276]**	HEROES V-A: HEmoRrhagic cOmplications in veno-arterial Extracorporeal life Support: Development and internal validation of a multivariable prediction model in adult patients	Insufficient data on anticoagulation
**Worku ET, et al[277]**	Haematological Trends and Transfusion during Adult Extracorporeal Membrane Oxygenation: A Single Centre Study	Non-comparative study
**Wu X et al[278]**	Risk factors for intracranial hemorrhage and mortality in adult patients with severe respiratory failure managed using veno-venous extracorporeal membrane oxygenation	Non-comparative study
**Yajun, et al[279]**	Application of extracorporeal membrane oxygenation in critically ill pregnant women	Pediatric patients
**Zhang J, et al[280]**	Veno-venous extracorporeal membrane oxygenation in coronavirus disease 2019: a case series	Non-comparative study
**Zhang, et al[281]**	A systematic review and meta-analysis of anticoagulation practices and mortality in extracorporeal cardiopulmonary resuscitation	Wrong study design
**Zhang, et al[282]**	Application of argatroban in patients receiving extracorporeal membrane oxygenation support: a case-control study	Pediatric patients
**Zhao, et al[283]**	Extracorporeal membrane oxygenation support for lung transplantation: Initial experience in a single center in China and a literature review	ECMO shorter than 24 hours
**Zichichi, et al[284]**	Systemic Versus Conservative Anticoagulation in Venovenous Extracorporeal Membrane Oxygenation	Wrong study design
**Basken et al[285]**	Predictors of Oxygenator Exchange in Patients Receiving Extracorporeal Membrane Oxygenation	Wrong research question
**Bikdeli et al[286]**	Intermediate-Dose versus Standard-Dose Prophylactic Anticoagulation in Patients with COVID-19 Admitted to the Intensive Care Unit: 90-Day Results from the INSPIRATION Randomized Trial	Non-comparative study
**Chan et al[287]**	Bloodless lung transplantation: Comparison between 2 central venoarterial extracorporeal membrane oxygenation anticoagulation strategies and their impact on lung transplant outcomes.	Non-comparative study
**Dorsey et al[288]**	Decreased bleeding and thrombotic complications on extracorporeal membrane oxygenation support following an updated anticoagulation protocol.	Insufficient data on anticoagulation
**Durila et al[289]**	Intravenous enoxaparin guided by anti-Xa in venovenous extracorporeal membrane oxygenation: A retrospective, single-center study	Non-comparative study

**Flinspach et al**[290]	Bleeding Complications in COVID-19 Critically Ill ARDS Patients Receiving VV-ECMO Therapy	Wrong study design
**Gutierrez et al**[291]	Outcomes Associated with the Use of Dual Antiplatelet Therapy in Patients Presenting with out of Hospital Cardiac Arrest Supported with Veno Arterial Extracorporeal Membrane Oxygenation	ECMO shorter than 24 hours
**Halawi et al**[292]	Use of bivalirudin after initial heparin management among adult patients on long-term venovenous extracorporeal support as a bridge to lung transplant: A case series.	Wrong study design
**Hickey et al**[293]	Systematic Review of Major Bleeding for patients with Covid-19 on ECMO	SR with all studies included
**Juthani et al**[294]	Outcomes of Adult Venovenous Extracorporeal Membrane Oxygenation Patients without Anticoagulation: A Retrospective Review at a Tertiary Level Referral Center	Non-comparative study
**Kasuya et al**[295]	Effects of shorter activated coagulation time on hemorrhage during venoarterial extracorporeal membrane oxygenation.	SR with all studies included
**Khoury et al**[296]	Low vs High aPTT-Guided Bivalirudin Anticoagulation in ECMO: Single- Center Retrospective Review of Outcomes	Non-comparative study
**Kuzmin et al**[297]	Anticoagulation management during veno-venous ECMO support because of ARDS: Single-center experience.	Insufficient data on anticoagulation
**Miller et al**[298]	Thromboembolic Complications in Continuous Versus Interrupted Anticoagulation During Venovenous Extracorporeal Membrane Oxygenation: A Multicenter Study.	Non-comparative study
**Naser et al**[299]	ECMO for polytrauma patients: a blessing in disguise or a trail of fiction	SR with all studies included
**Qi et al**[300]	The impact of early perioperative heparin-free anticoagulation for extracorporeal membrane oxygenation on bleeding and thrombotic events in lung transplantation: a retrospective cohort study.	Insufficient data on anticoagulation
**Sather et al**[301]	Thrombotic and Bleeding Complications in Continuous vs Intermittent Anticoagulation in Extracorporeal Membrane Oxygenation (ECMO): A Multi-Center Experience	Insufficient data on anticoagulation
**Schiavoni et al**[302]	Anticoagulation Management for Veno-Venous ECMO in COVID-19 Patients: Argatroban as Rescue Therapy in Heparin-Associated Thrombocytopenia.	Insufficient data on anticoagulation
**Vlok et al**[303]	Anticoagulation and associated complications in veno-arterial extracorporeal membrane oxygenation in adult patients: A systematic review and meta-analysis.	SR with all studies included

**Ward et al**[304]	Thrombotic and Hemorrhagic Outcomes in Venovenous Extracorporeal Membrane Oxygenation with or without Systemic Anticoagulation	SR with all studies included

1. Abbasciano RG, Yusuff H, Vlaar APJ, Lai F, Murphy GJ: Blood Transfusion Threshold in Patients Receiving Extracorporeal Membrane Oxygenation Support for Cardiac and Respiratory Failure—A Systematic Review and Meta-Analysis. Journal of Cardiothoracic and Vascular Anesthesia 2021; 35:1192–202
2. Abruzzo A, Gorantla V, Thomas SE: Venous thromboembolic events in the setting of extracorporeal membrane oxygenation support in adults: A systematic review. Thrombosis Research 2022; 212:58–71
3. Ahmad SB, Menaker J, Kufera J, O'Connor J, Scalea TM, Stein DM: Extracorporeal membrane oxygenation after traumatic injury. Journal of Trauma and Acute Care Surgery 2017; 82:587
4. Akhtar W, Olusanya O, Baladia MM, Young H, Shah S: SARS-CoV-2 and ECMO: early results and experience. Indian J Thorac Cardiovasc Surg 2021; 37:53–60
5. Al Abbasi B, Torres P, Grajeda E, et al.: Veno-Arterial Extracorporeal Membrane Oxygenation Support (VA-ECMO) in acute coronary syndrome complicated by cardiogenic shock. Clinical outcomes. Eur J Heart Fail 2021; 23:207
6. Al-Jazairi A, Raslan S, Al-mehizia R, et al.: Performance Assessment of a Multifaceted Unfractionated Heparin Dosing Protocol in Adult Patients on Extracorporeal Membrane Oxygenator. Ann Pharmacother 2021; 55:592–604
7. Alkazemi A, Eche IM, Adra M, et al.: Conventional versus restricted anti-Xa-guided heparin protocol in adult patients undergoing extracorporeal membrane oxygenation. Artificial Organs 2022; 46:128–37
8. Amos T, Bannon-Murphy H, Yeung M, et al.: ECMO (extra corporeal membrane oxygenation) in major trauma: A 10 year single centre experience. Injury 2021; 52:2515–21
9. Anderson H, Steimle C, Shapiro M, et al.: Extracorporeal life support for adult cardiorespiratory failure. Surgery 1993; 114:161–72; discussion 172-173
10. Ang AL, Teo D, Lim CH, Leou KK, Tien SL, Koh MBC: Blood transfusion requirements and independent predictors of increased transfusion requirements among adult patients on extracorporeal membrane oxygenation – a single centre experience. Vox Sanguinis 2009; 96:34–43
11. Arachchillage DJ, Remmington C, Rosenberg A, et al.: Anticoagulation with argatroban in patients with acute antithrombin deficiency in severe COVID-19. British Journal of Haematology 2020; 190:e286–8
12. Arachchillage D, Passariello M, Laffan M, et al.: Intracranial Hemorrhage and Early Mortality in Patients Receiving Extracorporeal Membrane Oxygenation for Severe Respiratory Failure. Semin Thromb Hemost 2018; 44:276–86
13. Arlt M, Philipp A, Voelkel S, et al.: Extracorporeal membrane oxygenation in severe trauma patients with bleeding shock. Resuscitation 2010; 81:804–9
14. Arnouk S, Altshuler D, Lewis TC, et al.: Evaluation of Anti-Xa and Activated Partial Thromboplastin Time Monitoring of Heparin in Adult Patients Receiving Extracorporeal Membrane Oxygenation Support. ASAIO Journal 2020; 66:300
15. Atallah S, Liebl M, Fitousis K, Bostan F, Masud F: Evaluation of the activated clotting time and activated partial thromboplastin time for the monitoring of heparin in adult extracorporeal membrane oxygenation patients. Perfusion 2014; 29:456–61
16. Aubron C, DePuydt J, Belon F, et al.: Predictive factors of bleeding events in adults undergoing extracorporeal membrane oxygenation. Ann Intensive Care 2016; 6

17. Aubron C, McQuilten Z, Bailey M, et al.: Low-Dose Versus Therapeutic Anticoagulation in Patients on Extracorporeal Membrane Oxygenation: A Pilot Randomized Trial. Critical Care Medicine 2019; 47:e563
18. Autschbach T, Hatam N, Durak K, et al.: Outcomes of extracorporeal membrane oxygenation for acute respiratory distress syndrome in covid-19 patients: A propensity-matched analysis. J Clin Med 2021; 10
19. Bakdach D, Akkari A, Gazwi K, et al.: Propofol Safety in Anticoagulated and Nonanticoagulated Patients During Extracorporeal Membrane Oxygenation. ASAIO J 2020:10.1097/MAT.0000000000001207 doi:10.1097/MAT.0000000000001207
20. Bastia L, Bombino M, Giani M, et al.: Management of venovenous extracorporeal membrane oxygenation with moderate-target anticoagulation: A retrospective study. Intensive Care Med Exp 2018; 6
21. Bedeir K, Seethala R, Kelly E: Extracorporeal life support in trauma: Worth the risks? A systematic review of published series. Journal of Trauma and Acute Care Surgery 2017; 82:400
22. Bemtgen X, Zotzmann V, Benk C, et al.: Thrombotic circuit complications during venovenous extracorporeal membrane oxygenation in COVID-19. J Thromb Thrombolysis 2021; 51:301–7
23. Biancari F, Mariscalco G, Dalén M, et al.: Six-Month Survival After Extracorporeal Membrane Oxygenation for Severe COVID-19. Journal of Cardiothoracic and Vascular Anesthesia 2021; 35:1999–2006
24. Bissell BD, Gabbard T, Sheridan EA, Baz MA, Davis GA, Ather A: Evaluation of Bivalirudin as the Primary Anticoagulant in Patients Receiving Extracorporeal Membrane Oxygenation for SARS-CoV-2–Associated Acute Respiratory Failure. Ann Pharmacother 2022; 56:387–92
25. Boddi M, Cianchi G, Cappellini E, et al.: Cannula-related deep vein thrombosis during extracorporeal membrane oxygenation (ECMO) treatment do not affect mortality and length of stay in intensive care unit. Crit Care 2017; 21
26. Brown MA, Najam F, Pocock ES, Munoz PF, Farrar KA, Yamane DP: A comparison of bivalirudin and heparin for patients on extracorporeal membrane oxygenation. Thromb Res 2020; 190:76–8
27. Buchtele N, Schörgenhofer C, Schwameis M, et al.: Add-On Prostaglandin E1 in Venovenous Extracorporeal Membrane Oxygenation: A Randomized, Double-Blind, Placebo-controlled Pilot Trial. Am J Respir Crit Care Med 2022; 206:170–7
28. Buscher H, Vukomanovic A, Benzimra M, Okada K, Nair P: Blood and Anticoagulation Management in Extracorporeal Membrane Oxygenation for Surgical and Nonsurgical Patients: A Single-Center Retrospective Review. Journal of Cardiothoracic and Vascular Anesthesia 2017; 31:869–75
29. Cahill CM, Blumberg N, Schmidt AE, et al.: Implementation of a Standardized Transfusion Protocol for Cardiac Patients Treated With Venoarterial Extracorporeal Membrane Oxygenation Is Associated With Decreased Blood Component Utilization and May Improve Clinical Outcome. Anesthesia & Analgesia 2018; 126:1262
30. Carter KT, Kutcher ME, Shake JG, et al.: Heparin-Sparing Anticoagulation Strategies Are Viable Options for Patients on Veno-Venous ECMO. Journal of Surgical Research 2019; 243:399–409
31. Cartwright B, Bruce HM, Kershaw G, et al.: Hemostasis, coagulation and thrombin in venoarterial and venovenous extracorporeal membrane oxygenation: the HECTIC study. Sci Rep 2021; 11:7975
32. Cercone JL, Kram SJ, Trammel MA, et al.: Effect of Initial Anticoagulation Targets on Bleeding and Thrombotic Complications for Patients With Acute Respiratory Distress Syndrome Receiving Extracorporeal Membrane Oxygenation. J Cardiothorac Vasc Anesth 2022; 36:3561–9

33. Chen T-H, James Y-MS, Shih JJ-M: Early percutaneous heparin-free veno-venous extra corporeal life support (ECLS) is a safe and effectivemeans of salvaging hypoxemic patients with complicated chest trauma. Acta Cardiol Sin 2016; 32:96–102
34. Chong WH, Saha BK, Medarov BI: Clinical Characteristics Between Survivors and Nonsurvivors of COVID-19 Patients Requiring Extracorporeal Membrane Oxygenation (ECMO) Support: A Systematic Review and Meta-Analysis. J Intensive Care Med 2022; 37:304–18
35. Chung YS, Cho DY, Sohn DS, et al.: Is Stopping Heparin Safe in Patients on Extracorporeal Membrane Oxygenation Treatment? ASAIO Journal 2017; 63:32
36. Cianchi G, Bonizzoli M, Pasquini A, et al.: Ventilatory and ECMO treatment of H1N1-induced severe respiratory failure: results of an Italian referral ECMO center. BMC Pulmonary Medicine 2011; 11:2
37. Ciolek AM, Ma K, Garan AR, Eisenberger AB, Jennings DL: Use of cangrelor during venoarterial extracorporeal membrane oxygenation following percutaneous coronary intervention. Artificial Organs 2020; 44:339–40
38. Colman E, Yin EB, Laine G, et al.: Evaluation of a heparin monitoring protocol for extracorporeal membrane oxygenation and review of the literature. Journal of Thoracic Disease 2019; 11
39. Cornell T, Wyrick P, Fleming G, et al.: A Case Series Describing the Use of Argatroban in Patients on Extracorporeal Circulation. ASAIO Journal 2007; 53:460
40. Corsi F, Lebreton G, Bréchot N, et al.: Life-threatening massive pulmonary embolism rescued by venoarterial-extracorporeal membrane oxygenation. Critical Care 2017; 21:76
41. Cousin N, Bourel C, Carpentier D, et al.: SARS-CoV-2 Versus Influenza-associated Acute Respiratory Distress Syndrome Requiring Veno-venous Extracorporeal Membrane Oxygenation Support. ASAIO Journal 2021; 67:125
42. Cunningham D, Besser MW, Giraud K, Gerrard C, Vuylsteke A: Agreement between ACT and aPTT during extracorporeal membrane oxygenation shows intra- and inter-individual variation. Perfusion 2016; 31:503–7
43. Deatrick KB, Galvagno SM, Mazzeffi MA, et al.: Pilot study evaluating a non-titrating, weight-based anticoagulation scheme for patients on veno-venous extracorporeal membrane oxygenation. Perfusion 2020; 35:13–8
44. Delmas C, Jacquemin A, Vardon-Bounes F, et al.: Anticoagulation Monitoring Under ECMO Support: A Comparative Study Between the Activated Coagulation Time and the Anti-Xa Activity Assay. J Intensive Care Med 2020; 35:679–86
45. Descamps R, Moussa MD, Besnier E, et al.: Anti-Xa activity and hemorrhagic events under extracorporeal membrane oxygenation (ECMO): a multicenter cohort study. Critical Care 2021; 25:127
46. Dognon N, Gaudet A, Parmentier-Decrucq E, et al.: Extracorporeal Membrane Oxygenation for COVID 2019-Acute Respiratory Distress Syndrome: Comparison between First and Second Waves (Stage 2). Journal of Clinical Medicine 2021; 10:4839
47. Doyle AJ, Hunt BJ, Sanderson B, et al.: A Comparison of Thrombosis and Hemorrhage Rates in Patients With Severe Respiratory Failure Due to Coronavirus Disease 2019 and Influenza Requiring Extracorporeal Membrane Oxygenation. Critical Care Medicine 2021; 49:e663
48. Durak K, Zayat R, Grottke O, et al.: Extracorporeal membrane oxygenation in patients with COVID-19: 1-year experience. Journal of Thoracic Disease 2021; 13
49. Durak K, Kersten A, Grottke O, et al.: Thromboembolic and Bleeding Events in COVID-19 Patients receiving Extracorporeal Membrane Oxygenation. Thorac Cardiovasc Surg 2021; 69:526–36

50. Esper SA, Bermudez C, Dueweke EJ, et al.: Extracorporeal membrane oxygenation support in acute coronary syndromes complicated by cardiogenic shock. Catheterization and Cardiovascular Interventions 2015; 86:S45–50
51. Feih JT, Wallskog KE, Rinka JRG, et al.: Heparin Monitoring with an Anti-Xa Protocol Compared to Activated Clotting Time in Patients on Temporary Mechanical Circulatory Support. Ann Pharmacother 2022; 56:513–23
52. Fina D, Matteucci M, Jiritano F, et al.: Extracorporeal membrane oxygenation without therapeutic anticoagulation in adults: A systematic review of the current literature. Int J Artif Organs 2020; 43:570–8
53. Fisser C: [Argatroban in patients with venovenous extracorporeal membrane oxygenation]. Med Klin Intensivmed Notfmed 2022; 117:473–5
54. Fisser C, Armbrüster C, Wiest C, et al.: Arterial and venous vascular complications in patients requiring peripheral venoarterial extracorporeal membrane oxygenation. Frontiers in Medicine 2022; 9
55. Fisser C, Reichenbächer C, Lubnow M, Philipp A, Lunz D, Müller T: Incidence and risk factors of venous thrombosis after venovenous extracorporeal membrane oxygenation in patients with acute respiratory distress syndrome: A prospective observational study. Eur J Heart Fail 2017; 19:28
56. Fitousis K, Klasek R, Mason PE, Masud F: Evaluation of a pharmacy managed heparin protocol for extracorporeal membrane oxygenation patients. Perfusion 2017; 32:238–44
57. Fletcher-Sandersjöö A, Thelin EP, Bartek J, et al.: Incidence, Outcome, and Predictors of Intracranial Hemorrhage in Adult Patients on Extracorporeal Membrane Oxygenation: A Systematic and Narrative Review. Frontiers in Neurology 2018; 9
58. Fletcher-Sandersjöö A, Thelin EP, Jr JB, Elmi-Terander A, Broman M, Bellander B-M: Management of intracranial hemorrhage in adult patients on extracorporeal membrane oxygenation (ECMO): An observational cohort study. PLOS ONE 2017; 12:e0190365
59. Fong KM, Au SY, Ng GWY, Leung AKH: Bleeding, thrombosis and transfusion in patients on ECMO: A retrospective study in a tertiary center in Hong Kong. Int J Artif Organs 2021; 44:420–5
60. Geli J, Capoccia M, Maybauer DM, Maybauer MO: Argatroban Anticoagulation for Adult Extracorporeal Membrane Oxygenation: A Systematic Review. J Intensive Care Med 2022; 37:459–71
61. Giuliano K, Bigelow BF, Etchill EW, et al.: Extracorporeal Membrane Oxygenation Complications in Heparin- and Bivalirudin-Treated Patients. Crit Care Explor 2021; 3:e0485
62. Glick D, Dzierba AL, Abrams D, et al.: Clinically suspected heparin-induced thrombocytopenia during extracorporeal membrane oxygenation. Journal of Critical Care 2015; 30:1190–4
63. Guliani S, Das Gupta J, Osofsky R, et al.: Venoarterial extracorporeal membrane oxygenation is an effective management strategy for massive pulmonary embolism patients. Journal of Vascular Surgery: Venous and Lymphatic Disorders 2021; 9:307–14
64. Guo Z, Sun L, Li B, et al.: Anticoagulation Management in Severe Coronavirus Disease 2019 Patients on Extracorporeal Membrane Oxygenation. Journal of Cardiothoracic and Vascular Anesthesia 2021; 35:389–97
65. Han W, Bok JS, Cho HJ, et al.: Single-center experience of extracorporeal membrane oxygenation mainly anticoagulated with nafamostat mesilate. Journal of Thoracic Disease 2019; 11
66. Han X, Yao Y: Mechanical Circulatory Support in Cardiovascular Surgical Patients: Single Center Practice and Experience. RCM 2022; 23:291

67. Hanna DJ, Torbic H, Militello M, Strnad K, Krishnan S, Hohlfelder B: Evaluation of anticoagulation with bivalirudin for heparin-induced thrombocytopenia during extracorporeal membrane oxygenation. Int J Artif Organs 2022; 45:688–94
68. Harahwa T, Chor CYT, Harky A: The use of extracorporeal membrane oxygenation postcardiotomy—A systematic review. Journal of Cardiac Surgery 2020; 35:1941–53
69. Hartley EL, Singh N, Barrett N, Wyncoll D, Retter A: Screening pulmonary angiogram and the effect on anticoagulation strategies in severe respiratory failure patients on venovenous extracorporeal membrane oxygenation. Journal of Thrombosis and Haemostasis 2020; 18:217–21
70. Hasegawa D, Sato R, Prasitlumkum N, et al.: Comparison of Bivalirudin Versus Heparin for Anticoagulation During Extracorporeal Membrane Oxygenation. ASAIO Journal 10.1097/MAT.0000000000001814 doi:10.1097/MAT.0000000000001814
71. Hékimian G, Masi P, Lejeune M, et al.: Extracorporeal Membrane Oxygenation Induces Early Alterations in Coagulation and Fibrinolysis Profiles in COVID-19 Patients with Acute Respiratory Distress Syndrome. Thromb Haemost 2021; 121:1031–42
72. Hemmila MR, Rowe SA, Boules TN, et al.: Extracorporeal Life Support for Severe Acute Respiratory Distress Syndrome in Adults. Ann Surg 2004; 240:595–607
73. Hermann A, Schellongowski P, Bojic A, Robak O, Buchtele N, Staudinger T: ECMO without anticoagulation in patients with disease-related severe thrombocytopenia: Feasible but futile? Artificial Organs 2019; 43:1077–84
74. Hohlfelder B, Kelly D, Hoang M, et al.: Activated Clotting Times Demonstrate Weak Correlation With Heparin Dosing in Adult Extracorporeal Membrane Oxygenation. American Journal of Therapeutics 2022; 29:e385
75. Holzer F, Gruber M, Philipp A, Lunz D, Graf B, Redel A: Predictors of bleeding in ECMOpatients undergoing surgery. Minerva Anestesiol 2020; 86:47–55
76. Hong JI, Hwang J, Shin HJ: Satisfactory outcome with low activated clotting time in extracorporeal membrane oxygenation. RCM 2021; 22:1589–94
77. Hou D, Wang H, Yang F, Hou X: Neurologic Complications in Adult Post-cardiotomy Cardiogenic Shock Patients Receiving Venoarterial Extracorporeal Membrane Oxygenation: A Cohort Study. Frontiers in Medicine 2021; 8
78. Hou X, Guo L, Zhan Q, et al.: Extracorporeal Membrane Oxygenation for Critically Ill Patients With 2009 Influenza A (H1N1)-Related Acute Respiratory Distress Syndrome: Preliminary Experience From a Single Center. Artificial Organs 2012; 36:780–6
79. Hu S, Lu A, Pan C, et al.: Limb Ischemia Complications of Veno-Arterial Extracorporeal Membrane Oxygenation. Frontiers in Medicine 2022; 9
80. Huang D, Guan Q, Qin J, Shan R, Wu J, Zhang C: Bivalirudin versus heparin anticoagulation in patients receiving extracorporeal membrane oxygenation. Perfusion 2022:02676591221105605 doi:10.1177/02676591221105605
81. Huang W, Ye H, Cheng Z, et al.: Outcomes from the Use of Perioperative Extracorporeal Membrane Oxygenation in Patients Undergoing Thoracic Surgery: An 8-Year Single-Center Experience. Med Sci Monit 2021; 27
82. Huprikar NA, Peterson MR, DellaVolpe JD, et al.: Salvage extracorporeal membrane oxygenation in induction-associated acute respiratory distress syndrome in acute leukemia patients: A case series. Int J Artif Organs 2019; 42:49–54
83. Ius F, Sommer W, Tudorache I, et al.: Veno-veno-arterial extracorporeal membrane oxygenation for respiratory failure with severe haemodynamic impairment: technique and early outcomes. Interactive CardioVascular and Thoracic Surgery 2015; 20:761–7
84. Jacobs JP, Stammers AH, St Louis JD, et al.: Multi-institutional Analysis of 200 COVID-19 Patients Treated With Extracorporeal Membrane Oxygenation: Outcomes and Trends. The Annals of Thoracic Surgery 2022; 113:1452–60

85. Jang J, Koo S-M, Kim K-U, et al.: Clinical Experiences of High-Risk Pulmonary Thromboembolism Receiving Extracorporeal Membrane Oxygenation in Single Institution. Tuberc Respir Dis 2022; 85:249–55
86. Jin Y, Zhang Y, Liu J: Ischemic stroke and intracranial hemorrhage in extracorporeal membrane oxygenation for COVID-19: A systematic review and meta-analysis. Perfusion 2022:02676591221130886 doi:10.1177/02676591221130886
87. Jiritano F, Fina D, Lorusso R, et al.: Systematic review and meta-analysis of the clinical effectiveness of point-of-care testing for anticoagulation management during ECMO. J Clin Anesth 2021; 73
88. Jiritano F, Serraino GF, Cate H ten, et al.: Platelets and extra-corporeal membrane oxygenation in adult patients: a systematic review and meta-analysis. Intensive Care Med 2020; 46:1154–69
89. Kakar V, North A, Bajwa G, Raposo N, Kumar GP: Long Runs and Higher Incidence of Bleeding Complications in COVID-19 Patients Requiring Venovenous Extracorporeal Membrane Oxygenation: A Case Series from the United Arab Emirates. Indian Journal of Critical Care Medicine 2021; 25:1452–8
90. Kalbhenn J, Glonnegger H, Wilke M, Bansbach J, Zieger B: Hypercoagulopathy, acquired coagulation disorders and anticoagulation before, during and after extracorporeal membrane oxygenation in COVID-19: a case series. Perfusion 2021; 36:592–602
91. Kannapadi NV, Jami M, Premraj L, et al.: Neurological Complications in COVID-19 Patients With ECMO Support: A Systematic Review and Meta-Analysis. Heart, Lung and Circulation 2022; 31:292–8
92. Kannapadi NV, Jami M, Premraj L, et al.: Neurologic Injury in Patients With COVID-19 Who Receive VV-ECMO Therapy: A Cohort Study. Journal of Cardiothoracic and Vascular Anesthesia 2021; 35:3456–61
93. Karim AS, Son AY, Suen R, et al.: Pre-Intubation Veno-Venous Extracorporeal Membrane Oxygenation in Patients at Risk for Respiratory Decompensation. J Extra Corpor Technol 2020; 52:52–7
94. Karampinis I, Al-Shammari A, Hartley P, et al.: Thoracic surgery in patients on veno-venous extracorporeal membrane oxygenation for COVID-19 associated acute respiratory distress syndrome. Perfusion 2022:02676591221090618 doi:10.1177/02676591221090618
95. Kim BK, Hong JI, Hwang J, Shin HJ: Satisfactory outcome with activated clotting time <160 seconds in extracorporeal cardiopulmonary resuscitation. Medicine 2022; 101:e30568
96. Kim YS, Lee H, Yang J-H, et al.: Use of argatroban for extracorporeal life support in patients with nonheparin-induced thrombocytopenia: Analysis of 10 consecutive patients. Medicine 2018; 97:e13235
97. Klinzing S, Wenger U, Stretti F, et al.: Neurologic Injury With Severe Adult Respiratory Distress Syndrome in Patients Undergoing Extracorporeal Membrane Oxygenation: A Single-Center Retrospective Analysis. Anesthesia & Analgesia 2017; 125:1544
98. Kohs TCL, Liu P, Raghunathan V, et al.: Severe thrombocytopenia in adults undergoing extracorporeal membrane oxygenation is predictive of thrombosis. Platelets 2022; 33:570–6
99. Kolla S, Lee WA, Hirschl RB, Bartlett RH: Extracorporeal Life Support for Cardiovascular Support in Adults. ASAIO Journal 1996; 42:M809
100. Kolla S, Awad SS, Rich PB, Schreiner RJ, Hirschl RB, Bartlett RH: Extracorporeal life support for 100 adult patients with severe respiratory failure. Ann Surg 1997; 226:544–66
101. Kon ZN, Smith DE, Chang SH, et al.: Extracorporeal Membrane Oxygenation Support in Severe COVID-19. The Annals of Thoracic Surgery 2021; 111:537–43

102. Kreyer S, Muders T, Theuerkauf N, et al.: Hemorrhage under veno-venous extracorporeal membrane oxygenation in acute respiratory distress syndrome patients: a retrospective data analysis. Journal of Thoracic Disease 2017; 9
103. Krueger K, Schmutz A, Zieger B, Kalbhenn J: Venovenous Extracorporeal Membrane Oxygenation With Prophylactic Subcutaneous Anticoagulation Only: An Observational Study in More Than 60 Patients. Artificial Organs 2017; 41:186–92
104. Kucuk AO, Küçük MP, Ayçiçek O, Altun G, Özdemir AC: Extracorporeal membrane oxygenation experiences during COVID-19 pandemic, third wave with younger patients: A retrospective observational study. Turkish Journal of Emergency Medicine 2022; 22:36
105. Kunavarapu C, Yeramaneni S, Melo J, et al.: Clinical outcomes of severe COVID-19 patients receiving early VV-ECMO and the impact of pre-ECMO ventilator use. Int J Artif Organs 2021; 44:861–7
106. Kurihara C, Walter JM, Karim A, et al.: Feasibility of Venovenous Extracorporeal Membrane Oxygenation Without Systemic Anticoagulation. The Annals of Thoracic Surgery 2020; 110:1209–15
107. Kutleša M, Novokmet A, Josipović Mraović R, Baršić B: Venovenous extracorporeal membrane oxygenation for ARDS: outcome analysis of a Croatian referral center for respiratory ECMO. Wien Klin Wochenschr 2017; 129:497–502
108. La Via L, Currò JM, Sanfilippo F, et al.: Use of nafamostat mesilate for anticoagulation during extracorporeal membrane oxygenation: A systematic review. Perfusion 2022; 37:34–5
109. Percutaneous coronary intervention under extracorporeal membrane oxygenation support for high-risk acute myocardial infarction with cardiogenic shock - Record details - Embase at <https://www-embase-com.ez.srv.meduniwien.ac.at/records?subaction=viewrecord&rid=2&page=1&id=L351427977>
110. Lai W, Li S, Du Z, et al.: Severe Patients With ARDS With COVID-19 Treated With Extracorporeal Membrane Oxygenation in China: A Retrospective Study. Frontiers in Medicine 2021; 8
111. Laimoud M, Ahmed W: Acute neurological complications in adult patients with cardiogenic shock on veno-arterial extracorporeal membrane oxygenation support. The Egyptian Heart Journal 2020; 72:26
112. Laimoud M, Saad E, Koussayer S: Acute vascular complications of femoral veno-arterial ECMO: a single-centre retrospective study. The Egyptian Heart Journal 2021; 73:15
113. Laimoud M, Alanazi M, Qureshi R: Hospital outcomes after emergent peripheral veno-arterial extracorporeal membrane oxygenation in adult patients presenting with cardiogenic shock 2021 doi:10.22514/sv.2021.118
114. Laine A, Niemi T, Suojaranta-Ylinen R, et al.: Decreased maximum clot firmness in rotational thromboelastometry (ROTEM®) is associated with bleeding during extracorporeal mechanical circulatory support. Perfusion 2016; 31:625–33
115. Lamarche Y, Chow B, Bédard A, et al.: Thromboembolic Events in Patients on Extracorporeal Membrane Oxygenation without Anticoagulation. Innovations (Phila) 2010; 5:424–9
116. Lang CN, Dettinger JS, Berchtold-Herz M, et al.: Intracerebral Hemorrhage in COVID-19 Patients with Pulmonary Failure: A Propensity Score-Matched Registry Study. Neurocrit Care 2021; 34:739–47
117. Lankford AS, Chow JH, Jackson AM, et al.: Clinical Outcomes of Pregnant and Postpartum Extracorporeal Membrane Oxygenation Patients. Anesthesia & Analgesia 2021; 132:777

118. Li D, Sun M, Zhang J, Zhang C, Deng L, Jiang H: Is bivalirudin an alternative anticoagulant for extracorporeal membrane oxygenation (ECMO) patients? A systematic review and meta-analysis. Thrombosis Research 2022; 210:53–62
119. Li M, Shi J, Zhang J: Bivalirudin vs. heparin in paediatric and adult patients on extracorporeal membrane oxygenation: A meta-analysis. British Journal of Clinical Pharmacology 2022; 88:2605–16
120. Lin T-W, Tsai M-T, Hu Y-N, et al.: Simultaneous Thrombolysis and Extracorporeal Membrane Oxygenation for Acute Massive Pulmonary Emboli. The Annals of Thoracic Surgery 2021; 111:923–9
121. Liu L, Liu F, Tan J, Zhao L: Bivalirudin versus heparin in adult and pediatric patients with extracorporeal membrane oxygenation therapy: A systematic review and meta-analysis. Pharmacological Research 2022; 177:106089
122. Liu Y, Yuan Z, Han X, Song K, Xing J: A Comparison of Activated Partial Thromboplastin Time and Activated Coagulation Time for Anticoagulation Monitoring during Extracorporeal Membrane Oxygenation Therapy. Hamostaseologie 2022 doi:10.1055/a-1796-8652
123. Liu Z, Chen J, Xu X, et al.: Extracorporeal Membrane Oxygenation—First Strategy for Acute Life-Threatening Pulmonary Embolism. Frontiers in Cardiovascular Medicine 2022; 9
124. Loforte A, Marinelli G, Musumeci F, et al.: Extracorporeal Membrane Oxygenation Support in Refractory Cardiogenic Shock: Treatment Strategies and Analysis of Risk Factors. Artificial Organs 2014; 38:E129–41
125. López Sánchez M, Rubio López MI, Rodríguez Borregán JC, et al.: Mechanical complications of extracorporeal membrane oxygenation. Intensive Care Med 2013; 39:S304
126. Lubnow M, Berger J, Schneckenpointner R, et al.: Prevalence and outcomes of patients developing heparin-induced thrombocytopenia during extracorporeal membrane oxygenation. PLOS ONE 2022; 17:e0272577
127. Lv X, Deng M, Wang L, Dong Y, Chen L, Dai X: Low vs standardized dose anticoagulation regimens for extracorporeal membrane oxygenation: A meta-analysis. PLOS ONE 2021; 16:e0249854
128. M'Pembele R, Roth S, Metzger A, et al.: Evaluation of clinical outcomes in patients treated with heparin or direct thrombin inhibitors during extracorporeal membrane oxygenation: a systematic review and meta-analysis. Thrombosis Journal 2022; 20:42
129. Ma M, Liang S, Zhu J, et al.: The Efficacy and Safety of Bivalirudin Versus Heparin in the Anticoagulation Therapy of Extracorporeal Membrane Oxygenation: A Systematic Review and Meta-Analysis. Front Pharmacol 2022; 13:771563
130. Macielak S, Burcham P, Whitson B, Abdel-Rasoul M, Rozycki A: Impact of anticoagulation strategy and agents on extracorporeal membrane oxygenation therapy. Perfusion 2019; 34:671–8
131. Martucci G, Panarello G, Occhipinti G, et al.: Anticoagulation and Transfusions Management in Veno-Venous Extracorporeal Membrane Oxygenation for Acute Respiratory Distress Syndrome: Assessment of Factors Associated With Transfusion Requirements and Mortality. J Intensive Care Med 2019; 34:630–9
132. Martucci G, Bianco C, Amata M, et al.: The role of endoscopy as non-invasive procedure to manage gastrointestinal complications during extracorporeal membrane oxygenation. Perfusion 2020; 35:164
133. Mazzeffi MA, Tanaka K, Roberts A, et al.: Bleeding, Thrombosis, and Transfusion With Two Heparin Anticoagulation Protocols in Venoarterial ECMO Patients. Journal of Cardiothoracic and Vascular Anesthesia 2019; 33:1216–20

134. Mazzeffi M, Greenwood J, Tanaka K, et al.: Bleeding, Transfusion, and Mortality on Extracorporeal Life Support: ECLS Working Group on Thrombosis and Hemostasis. The Annals of Thoracic Surgery 2016; 101:682–9
135. McQuilten Z, Aubron C, Bailey M, et al.: Low-Dose Heparin in Critically Ill Patients Undergoing Extracorporeal Membrane Oxygenation - the Help-ECMO Pilot Randomised Controlled Trial. Blood 2016; 128:3822
136. Mégarbane B, Mohebbi S, Deye N, Bellucci-Sessa S, Baud FJ: Coagulation status in poisonings requiring extracorporeal life support in relation to refractory cardiac failure or arrest: A prospective study. Intensive Care Med 2009; 35:S70
137. Melehy A, Sanchez J, Fried JA, Takeda K: Prosthetic valve thrombosis during extracorporeal life support for postcardiotomy shock. Interactive CardioVascular and Thoracic Surgery 2020; 31:573–5
138. Menk M, Briem P, Weiss B, et al.: Efficacy and safety of argatroban in patients with acute respiratory distress syndrome and extracorporeal lung support. Annals of Intensive Care 2017; 7:82
139. Mihu MR, Mageka D, Swant LV, et al.: Veno-arteriovenous extracorporeal membrane oxygenation—A single center experience. Artificial Organs 2021; 45:1554–61
140. Moussa MD, Soquet J, Lamer A, et al.: Evaluation of Anti-Activated Factor X Activity and Activated Partial Thromboplastin Time Relations and Their Association with Bleeding and Thrombosis during Veno-Arterial ECMO Support: A Retrospective Study. Journal of Clinical Medicine 2021; 10:2158
141. Murakami T, Sakakura K, Jinnouchi H, et al.: Comparison of medical resource use and total admission cost in patients with acute myocardial infarction between on-hours visit versus off-hours visit. Cardiovasc Intervention Ther 2022; 37:651–9
142. Netley J, Roy J, Greenlee J, Hart S, Todt M, Statz B: Bivalirudin Anticoagulation Dosing Protocol for Extracorporeal Membrane Oxygenation: A Retrospective Review. J Extra Corpor Technol 2018; 50:161–6
143. Ng GWY, Leung AKH, Sin KC, et al.: Three-year experience of using venovenous extracorporeal membrane oxygenation for patients with severe respiratory failure. Hong Kong Med J 2014; 20:407–12
144. Northam KA, Nguyen B, Chen S-L, Sredzienski E, Charles A: Evaluation of a Multimodal Heparin Laboratory Monitoring Protocol in Adult Extracorporeal Membrane Oxygenation Patients. Journal of Pharmacy Practice 2023; 36:79–86
145. Northam KA, Murray BP, Fischer WA, Charles AG: Major Bleeding and Thrombosis Events in COVID-19 Versus Influenza Patients Requiring Extracorporeal Membrane Oxygenation. ASAIO Journal 2022; 68:779
146. O'Malley TJ, Choi JH, Maynes EJ, et al.: Outcomes of extracorporeal life support for the treatment of acute massive pulmonary embolism: A systematic review. Resuscitation 2020; 146:132–7
147. Ogami T, Takayama H, Melehy A, et al.: A Standardized Approach Improves Outcomes of Extracorporeal Membrane Oxygenation for Postcardiotomy Shock. ASAIO Journal 2021; 67:1119
148. Olson SR, Murphree CR, Zonies D, et al.: Thrombosis and Bleeding in Extracorporeal Membrane Oxygenation (ECMO) Without Anticoagulation: A Systematic Review. ASAIO Journal 2021; 67:290
149. Oude Lansink-Hartgring A, Vries AJ de, Droogh JM, Bergh WM van den: Hemorrhagic complications during extracorporeal membrane oxygenation – The role of anticoagulation and platelets. Journal of Critical Care 2019; 54:239–43

150. Pabst D, Boone JB, Soleimani B, Brehm CE: Heparin-induced thrombocytopenia in patients on extracorporeal membrane oxygenation and the role of a heparin-bonded circuit. Perfusion 2019; 34:584–9
151. Panigada M, E. Iapichino G, Brioni M, et al.: Thromboelastography-based anticoagulation management during extracorporeal membrane oxygenation: a safety and feasibility pilot study. Annals of Intensive Care 2018; 8:7
152. Papadopoulos N, Marinos S, El-Sayed Ahmad A, et al.: Risk factors associated with adverse outcome following extracorporeal life support: analysis from 360 consecutive patients. Perfusion 2015; 30:284–90
153. Parker BM, Menaker J, Berry CD, et al.: Single Center Experience With Veno-Venous Extracorporeal Membrane Oxygenation in Patients With Traumatic Brain Injury. The American Surgeon 2021; 87:949–53
154. Pieri M, Agracheva N, Bonaveglio E, et al.: Bivalirudin Versus Heparin as an Anticoagulant During Extracorporeal Membrane Oxygenation: A Case-Control Study. Journal of Cardiothoracic and Vascular Anesthesia 2013; 27:30–4
155. Popugaev KA, Bakharev SA, Kiselev KV, et al.: Clinical and pathophysiologic aspects of ECMO-associated hemorrhagic complications. PLOS ONE 2020; 15:e0240117
156. Pozzi M, Buzzi R, Hayek A, et al.: Veno-arterial extracorporeal membrane oxygenation for drug intoxications: A single center, 14-year experience. Journal of Cardiac Surgery 2022; 37:1512–9
157. Pranikoff T, Hirschl RB, Steimle CN, Anderson HLI, Bartlett RH: Efficacy of Extracorporeal Life Support in the Setting of Adult Cardiorespiratory Failure. ASAIO Journal 1994; 40:M339
158. Reddy HG, Maynes EJ, Saxena A, et al.: Utilization of extracorporeal life support for diffuse alveolar damage and diffuse alveolar hemorrhage: A systematic review. Artificial Organs 2021; 45:559–68
159. Renaudier M, Roux Q de, Bougouin W, et al.: Acute mesenteric ischaemia in refractory shock on veno-arterial extracorporeal membrane oxygenation. European Heart Journal Acute Cardiovascular Care 2021; 10:62–70
160. Rhoades R, Leong R, Kopenitz J, et al.: Coagulopathy monitoring and anticoagulation management in COVID-19 patients on ECMO: Advantages of a heparin anti-Xa-based titration strategy. Thromb Res 2021; 203:1–4
161. Ried M, Sommerauer L, Lubnow M, et al.: Thoracic Bleeding Complications in Patients With Venovenous Extracorporeal Membrane Oxygenation. The Annals of Thoracic Surgery 2018; 106:1668–74
162. Riera J, Argudo E, Martínez-Martínez M, et al.: Extracorporeal Membrane Oxygenation Retrieval in Coronavirus Disease 2019: A Case-Series of 19 Patients Supported at a High-Volume Extracorporeal Membrane Oxygenation Center. Crit Care Explor 2020; 2:e0228
163. Ripoll B, Rubino A, Besser M, et al.: Observational study of thrombosis and bleeding in COVID-19 VV ECMO patients. Int J Artif Organs 2022; 45:239–42
164. Roedl K, Kahn A, Jarczak D, et al.: Clinical Characteristics, Complications and Outcomes of Patients with Severe Acute Respiratory Distress Syndrome Related to COVID-19 or Influenza Requiring Extracorporeal Membrane Oxygenation—A Retrospective Cohort Study. Journal of Clinical Medicine 2021; 10:5440
165. Sadeghipour P, Talasaz AH, Rashidi F, et al.: Effect of Intermediate-Dose vs Standard-Dose Prophylactic Anticoagulation on Thrombotic Events, Extracorporeal Membrane Oxygenation Treatment, or Mortality among Patients with COVID-19 Admitted to the Intensive Care Unit: The INSPIRATION Randomized Clinical Trial. JAMA 2021; 325:1620–30

166. Sanfilippo F, Asmussen S, Maybauer DM, et al.: Bivalirudin for Alternative Anticoagulation in Extracorporeal Membrane Oxygenation: A Systematic Review. J Intensive Care Med 2017; 32:312–9
167. Sanfilippo F, Currò JM, La Via L, et al.: Use of nafamostat mesilate for anticoagulation during extracorporeal membrane oxygenation: A systematic review. Artificial Organs 2022; 46:2371–81
168. Seeliger B, Döbler M, Friedrich R, et al.: Comparison of anticoagulation strategies for veno-venous ECMO support in acute respiratory failure. Critical Care 2021; 24:701
169. Seeliger B, Doebler M, Hofmaenner DA, et al.: Intracranial Hemorrhages on Extracorporeal Membrane Oxygenation: Differences Between COVID-19 and Other Viral Acute Respiratory Distress Syndrome. Critical Care Medicine 2022; 50:e526
170. Shah A, Pasrija C, Kronfli A, et al.: A Comparison of Anticoagulation Strategies in Veno-venous Extracorporeal Membrane Oxygenation. ASAIO Journal 2022; 68:738
171. Shih E, DiMaio JM, Squiers JJ, et al.: Venovenous extracorporeal membrane oxygenation for patients with refractory coronavirus disease 2019 (COVID-19): Multicenter experience of referral hospitals in a large health care system. The Journal of Thoracic and Cardiovascular Surgery 2022; 163:1071-1079.e3
172. Siatan JE, Burog AIL, Chavez J, Danguilan R, Catli-Burog CA: Incidence of clinically significant bleeding and thromboembolism among patients with leptospirosis requiring extra corporeal membrane oxygenation: A descriptive study. Perfusion 2022; 37:38–9
173. Sklar MC, Sy E, Lequier L, Fan E, Kanji HD: Anticoagulation Practices during Venovenous Extracorporeal Membrane Oxygenation for Respiratory Failure. A Systematic Review. Annals ATS 2016; 13:2242–50
174. Stammers AH, Tesdahl EA, Sestokas AK, et al.: Variation in outcomes with extracorporeal membrane oxygenation in the era of coronavirus: A multicenter cohort evaluation. Perfusion 2022:02676591221118321 doi:10.1177/02676591221118321
175. Staudacher DL, Biever PM, Benk C, Ahrens I, Bode C, Wengenmayer T: Dual Antiplatelet Therapy (DAPT) versus No Antiplatelet Therapy and Incidence of Major Bleeding in Patients on Venoarterial Extracorporeal Membrane Oxygenation. PLOS ONE 2016; 11:e0159973
176. Stokes JW, Gannon WD, Sherrill WH, et al.: Bleeding, Thromboembolism, and Clinical Outcomes in Venovenous Extracorporeal Membrane Oxygenation. Crit Care Explor 2020; 2:e0267
177. Stub D, Bernard S, Pellegrino V, et al.: Refractory cardiac arrest treated with mechanical CPR, hypothermia, ECMO and early reperfusion (the CHEER trial). Resuscitation 2015; 86:88–94
178. Sutter R, Tisljar K, Marsch S: Acute Neurologic Complications During Extracorporeal Membrane Oxygenation: A Systematic Review. Critical Care Medicine 2018; 46:1506
179. Sy E, Sklar MC, Lequier L, Fan E, Kanji HD: Anticoagulation practices and the prevalence of major bleeding, thromboembolic events, and mortality in venoarterial extracorporeal membrane oxygenation: A systematic review and meta-analysis. Journal of Critical Care 2017; 39:87–96
180. Trigonis R, Smith N, Porter S, et al.: Efficacy of Bivalirudin for Therapeutic Anticoagulation in COVID-19 Patients Requiring ECMO Support. Journal of Cardiothoracic and Vascular Anesthesia 2022; 36:414–8
181. Tsai M-T, Hsu C-H, Luo C-Y, Hu Y-N, Roan J-N: Bridge-to-recovery strategy using extracorporeal membrane oxygenation for critical pulmonary hypertension complicated with cardiogenic shock. Interactive CardioVascular and Thoracic Surgery 2015; 21:55–61

182. Umei N, Ichiba S, Genda Y, Mase H, Sakamoto A: Early predictors of oxygenator exchange during veno-venous extracorporeal membrane oxygenation: A retrospective analysis. Int J Artif Organs 2022; 45:927–35
183. Valente S, Lazzeri C, Bernardo P, et al.: Bleeding events in refractory cardiac arrest treated with extracorporeal membrane oxygenation - A single centre experience. Resuscitation 2013; 84:e119
184. Walker EA, Roberts AJ, Louie EL, Dager WE: Bivalirudin Dosing Requirements in Adult Patients on Extracorporeal Life Support With or Without Continuous Renal Replacement Therapy. ASAIO Journal 2019; 65:134
185. Wang C, Zhang L, Qin T, et al.: Extracorporeal membrane oxygenation in trauma patients: a systematic review. World Journal of Emergency Surgery 2020; 15:51
186. Weatherill A, Laffan M, Gasper M, et al.: Impact of Thrombosis and Bleeding in Patients with Severe COVID-19 versus Other Viral Pneumonias in the Context of Extracorporeal Membrane Oxygenation. Semin Thromb Hemost 2022; 48:118–23
187. Weber C, Deppe A-C, Sabashnikov A, et al.: Left ventricular thrombus formation in patients undergoing femoral veno-arterial extracorporeal membrane oxygenation. Perfusion 2018; 33:283–8
188. Weingart C, Lubnow M, Philipp A, Bein T, Camboni D, Müller T: Comparison of Coagulation Parameters, Anticoagulation, and Need for Transfusion in Patients on Interventional Lung Assist or Veno-Venous Extracorporeal Membrane Oxygenation. Artificial Organs 2015; 39:765–73
189. Weir-McCall JR, Galea G, Mun Mak S, et al.: Vascular Thrombosis in Severe COVID-19 Requiring Extracorporeal Membrane Oxygenation: A Multicenter Study. Critical Care Medicine 2022; 50:624
190. Wilcox C, Etchill E, Giuliano K, et al.: Acute Brain Injury in Postcardiotomy Shock Treated With Venoarterial Extracorporeal Membrane Oxygenation. Journal of Cardiothoracic and Vascular Anesthesia 2021; 35:1989–96
191. Willems A, Roeleveld PP, Labarinas S, et al.: Anti-Xa versus time-guided anticoagulation strategies in extracorporeal membrane oxygenation: a systematic review and meta-analysis. Perfusion 2021; 36:501–12
192. Willers A, Swol J, Kowalewski M, et al.: Extracorporeal Life Support in Hemorrhagic Conditions: A Systematic Review. ASAIO Journal 2021; 67:476
193. Wohlfarth P, Beutel G, Lebiedz P, et al.: Characteristics and Outcome of Patients After Allogeneic Hematopoietic Stem Cell Transplantation Treated With Extracorporeal Membrane Oxygenation for Acute Respiratory Distress Syndrome*. Critical Care Medicine 2017; 45:e500
194. Wood KL, Ayers B, Gosev I, et al.: Venoarterial-Extracorporeal Membrane Oxygenation Without Routine Systemic Anticoagulation Decreases Adverse Events. The Annals of Thoracic Surgery 2020; 109:1458–66
195. Yajun Q, Yingying H, Yang L, et al.: Application of extracorporeal membrane oxygenation in critically ill pregnant women. Zhonghua Wei Zhong Bing Ji Jiu Yi Xue 2021; 33:605–8
196. Yasuda N, Goto K, Mizoguchi T, Ohchi Y, Nureki S-I, Kitano T: A new anticoagulation strategy using recombinant human thrombomodulin in patients on veno-venous extracorporeal membrane oxygenation: a retrospective study. Annals of Palliative Medicine 2021; 10:1834841–1831841
197. Zhao Y-C, Zhao X, Fu G-W, et al.: Heparin-free after 3000 IU heparin loaded in veno-venous ECMO supported acute respiratory failure patients with hemorrhage risk: a novel anti-coagulation strategy. Thrombosis Journal 2022; 20:36
198. Abramov A, Su I, Agerstrand C, et al.: Extracorporeal Membrane Oxygenation Support in COVID-19 Patients: A Propensity Score Analysis. ASAIO J 2022; 68:E224–9

199. Abruzzo A, Gorantla V, Thomas SE: Venous thromboembolic events in the setting of extracorporeal membrane oxygenation support in adults: A systematic review. Thrombosis Research 2022; 212:58–71
200. Akkanti B, Suarez EE, O'Neil ER, et al.: Extracorporeal Membrane Oxygenation for COVID-19: Collaborative Experience from the Texas Medical Center in Houston with 2 Years Follow-Up. ASAIO J 2022; 68:1443–9
201. Aldairi N, Al Ali AS, Alabdulqader M, Al Jeraisy M, Cyrus J, Karam O: Efficacy of Prostacyclin Anticoagulation in Critically Ill Patients Requiring Extracorporeal Support: A Systematic Review and Meta-Analysis. Cureus 2023; 15:e39967
202. Alves Cabrita J, Gama M, Oliveira MJ, Fortuna P, Bento L: Anticoagulation in ECMO patients: Management in a portuguese center. Perfusion 2023; 38:178
203. Arachchillage DJ, Rajakaruna I, Laffan M, et al.: Outcomes in COVID-19 supported by ECMO in the first vs second wave -multicentre observational study. Br J Haematol 2023; 201:25
204. Araújo Silva R, Cunha V, Correia D, et al.: Risk factors for major neurological events in patients under venovenous extracorporeal membrane oxygenation - single centre study. Perfusion 2023; 38:180–1
205. Austin S, Powell E, Podell J, et al.: Veno-venous extracorporeal membrane oxygenation should be considered early in patients with traumatic brain injuries and severe respiratory failure. Perfusion 2023; 38:136
206. Baldetti L, Nardelli P, Ajello S, et al.: Anti-thrombotic Therapy With Cangrelor and Bivalirudin in Venoarterial Extracorporeal Membrane Oxygenation Patients Undergoing Percutaneous Coronary Intervention: A Single-Center Experience. ASAIO J 2023; 69:E346–50
207. Baumgartner S, Lubnow M, Malfertheiner MV, et al.: Major Bleeding and Thromboembolic Events in Veno-Venous Extracorporeal Membrane Oxygenation-Patients with Isolated Respiratory Failure. ASAIO J 2022; 68:1529–35
208. Bento Rodrigues AR, Gomes DC, Fernandes SM, Ribeiro JM: Hemorrhagic and thrombotic complications under VV-ECMO in patients with SARS-CoV-2 infection: learnings from a single center. Intensive Care Med Exp 2022; 10
209. Bermea RS, Raz Y, Sertic F, et al.: Increased Intracranial Hemorrhage Amid Elevated Inflammatory Markers in Those With COVID-19 Supported With Extracorporeal Membrane Oxygenation. Shock 2021; 56:206
210. Bernal AG, Kalra R, Chang K, et al.: THROMBOTIC COMPLICATIONS OF EXTRA-CORPOREAL CARDIOPULMONARY RESUSCITATION FOR VT/VF OUT OF HOSPITAL CARDIAC ARREST AND ASSOCIATED MORTALITY. J Am Coll Cardiol 2023; 81:680
211. Brasil LMCR, Arruda GN de, Diniz GBF, et al.: Veno-venous extracorporeal membrane oxygenation in patients with SARS-CoV-2 pneumonia in Brazil: a case series. J Bras Pneumol 2023; 49
212. Buckley MS, Benanti GE, Meckel J, et al.: Correlation between partial thromboplastin time and thromboelastography in adult critically ill patients requiring bivalirudin for extracorporeal membrane oxygenation. Pharmacotherapy 2023; 43:196–204
213. Buckley MS, Agarwal SK, Garcia-Orr R, Saggar R, MacLaren R: Comparison of Fixed-Dose Inhaled Epoprostenol and Inhaled Nitric Oxide for Acute Respiratory Distress Syndrome in Critically Ill Adults. J Intensive Care Med 2021; 36:466–76
214. Chang HW, Park YK, Kim KM, et al.: The safety of discontinuing heparin in patients with venoarterial ECMO. Perfusion 2023; 38:84
215. Chang K, Bernal AG, Kalra R, et al.: INCIDENCE AND RISK FACTORS ASSOCIATED WITH BLEEDING IN ADULTS UNDERGOING EXTRACORPOREAL CARDIOPULMONARY RESUSCITATION FOR VT/VF OUT OF HOSPITAL CARDIAC ARREST. J Am Coll Cardiol 2023; 81:799

216. Chen J, Chen G, Zhao W, Peng W: Anticoagulation strategies in patients with extracorporeal membrane oxygenation: A network meta-analysis and systematic review. Pharmacotherapy 2023 doi:10.1002/phar.2859
217. Cho S-M, Premraj L, Fanning J, et al.: Ischemic and Hemorrhagic Stroke Among Critically Ill Patients With Coronavirus Disease 2019: An International Multicenter Coronavirus Disease 2019 Critical Care Consortium Study. Crit Care Med 2021; 49:E1223–33
218. Cohan D, Uricchio MN, Konopka CI, Montepara CA, Verlinden NJ: Comparison of clinical outcomes with cangrelor plus aspirin versus oral dual antiplatelet therapy in patients supported with venoarterial extracorporeal membrane oxygenation. Artif Organs 2023 doi:10.1111/aor.14590
219. De Paulis S, Cavaliere F: Anticoagulation Management in High Bleeding-Risk ECMO in Adults. Front Cardiovasc Med 2022; 9
220. Diaz D, Martinez J, Bushman G, Wolowich WR: Anticoagulation strategies in COVID-19 infected patients receiving ECMO support. J Extra-Corpor Technol 2023; 55:121–9
221. El Banayosy AM, El Banayosy A, Smith JG, et al.: Extracorporeal life support in pregnant and postpartum women with COVID-19-related acute respiratory distress syndrome. Int J Artif Organs 2023; 46:289–94
222. Fisser C, Armbrüster C, Wiest C, et al.: Arterial and venous vascular complications in patients requiring peripheral venoarterial extracorporeal membrane oxygenation. Front Med 2022; 9
223. Golicnik A, Zivanovic I, Gorjup V, Berden J: Same but Different—ECMO in COVID-19 and ARDS of Other Etiologies. Comparison of Survival Outcomes and Management in Different ARDS Groups. J Intensive Care Med 2023; 38:635–42
224. Gu J, Yu H, Lin D: Superiority of bivalirudin over heparin anticoagulation therapy for extracorporeal membrane oxygenation? Too early to draw conclusions. Heliyon 2023; 9:e13530
225. Hanna DJ, Torbic H, Militello M, Strnad K, Krishnan S, Hohlfelder B: Evaluation of anticoagulation with bivalirudin for heparin-induced thrombocytopenia during extracorporeal membrane oxygenation. Int J Artif Organs 2022; 45:688–94
226. Hasegawa D, Sato R, Prasitlumkum N, et al.: Comparison of Bivalirudin Versus Heparin for Anticoagulation During Extracorporeal Membrane Oxygenation. ASAIO Journal 2023:10.1097/MAT.0000000000001814 doi:10.1097/MAT.0000000000001814
227. Hofmaenner DA, Furfaro D, Wild LC, et al.: Reduced anticoagulation strategy is associated with a lower incidence of intracerebral hemorrhage in COVID-19 patients on extracorporeal membrane oxygenation. Intensive Care Med Exp 2023; 11
228. Honda A, Michihata N, Iizuka Y, et al.: Risk factors for severe lower extremity ischemia following venoarterial extracorporeal membrane oxygenation: An analysis using a nationwide inpatient database. Trauma Surg Acute Care open 2022; 7
229. Huang Y-X, Xu Z-M, Zhao L, et al.: Long-term outcomes of high-risk percutaneous coronary interventions under extracorporeal membrane oxygenation support: An observational study. World J Clin Cases 2022; 10:5266–74
230. Iannattone PA, Yang SS, Koolian M, Wong EG, Lipes J: Incidence of Venous Thromboembolism in Adults Receiving Extracorporeal Membrane Oxygenation: A Systematic Review. ASAIO J 2022; 68:1523–8
231. Jacobs JP, Stammers AH, St. Louis JD, et al.: Variation in survival in patients with Coronavirus Disease 2019 supported with extracorporeal membrane oxygenation: A multi-institutional analysis of 594 consecutive patients with Coronavirus Disease 2019 supported with extracorporeal membrane oxygenation at 49 hospitals within 21 states. The Journal of Thoracic and Cardiovascular Surgery 2023; 165:1837–48

232. Jang J, Koo S-M, Kim K-U, et al.: Clinical Experiences of High-Risk Pulmonary Thromboembolism Receiving Extracorporeal Membrane Oxygenation in Single Institution. Tuberc Respir Dis 2022; 85:249–55
233. Jin Y, Zhang Y, Liu J, Zhou Z: Thrombosis and bleeding in patients with COVID-19 requiring extracorporeal membrane oxygenation: a systematic review and meta-analysis. Research and Practice in Thrombosis and Haemostasis 2023; 7:100103
234. Jones C, Chen K, Cheng G, Narendran V: Thromboembolism in COVID -19 patients on ECMO. Br J Haematol 2022; 197:152–3
235. Kawaji Q, Rostami S, Whitman GJR, Broderick SR, Aziz H, Cha SL: Bivalirudin and Venoarterial Extracorporeal Membrane Oxygenation for Treatment of Massive Pulmonary Embolism From Heparin-Induced Thrombocytopenia With Thrombosis. J Cardiothorac Vasc Anesth 2023; 37:777–81
236. Kido K, Kabulski GM, Szymanski TW, Shiga T, Shimizu M, Hashiguchi M: Meta-Analysis Comparing Bivalirudin Versus. Unfractionated Heparin in Adult Patients With Extracorporeal Membrane Oxygenation. Journal of Pharmacy Practice 2022:08971900221143406 doi:10.1177/08971900221143406
237. Kim SH, Huh U, Song S, Kim MS, Wang IJ, Tak YJ: Outcomes in trauma patients undergoing veno-venous extracorporeal membrane oxygenation for acute respiratory distress syndrome. Perfusion 2023; 38:1037–44
238. Kutleša M, Santini M, Krajinović V, et al.: Extracorporeal membrane oxygenation in COVID-19: Results of the Croatian Extracorporeal Membrane Oxygenation Referral Center. Int J Artif Organs 2023; 46:248–51
239. Lankford AS, Chow JH, Jackson AM, et al.: Clinical Outcomes of Pregnant and Postpartum Extracorporeal Membrane Oxygenation Patients. Anesthesia & Analgesia 2021; 132:777
240. Lannon M, Duda T, Greer A, et al.: Intracranial hemorrhage in patients treated for SARS-CoV-2 with extracorporeal membrane oxygenation: A systematic review and meta-analysis. J Crit Care 2023; 77
241. Lejeune M, Hekimian G, Masi P, et al.: Longitudinal measurement of whole blood viscoelastic properties throughout the course of extracorporeal membrane oxygenation in COVID-19 patients with severe acute respiratory distress syndrome. Res Pract Thromb Haemost 2021; 5
242. Li D, Sun M, Zhang J, Zhang C, Deng L, Jiang H: Is bivalirudin an alternative anticoagulant for extracorporeal membrane oxygenation (ECMO) patients? A systematic review and meta-analysis. Thrombosis Research 2022; 210:53–62
243. Lopez ND, Seto SL, Barra ME, et al.: Evaluation of Bivalirudin During Adult Extracorporeal Membrane Oxygenation: A Retrospective Characterization of Dosing, Efficacy and Bleeding. Hosp Pharm 2023:00185787231188924 doi:10.1177/00185787231188924
244. Lubnow M, Berger J, Schneckenpointner R, et al.: Prevalence and outcomes of patients developing heparin-induced thrombocytopenia during extracorporeal membrane oxygenation. PLOS ONE 2022; 17:e0272577
245. Lüsebrink E, Scherer C, Binzenhöfer L, et al.: Heparin-Induced Thrombocytopenia in Patients Undergoing Venoarterial Extracorporeal Membrane Oxygenation. J Clin Med 2023; 12
246. Ma M, Liang S, Zhu J, et al.: The Efficacy and Safety of Bivalirudin Versus Heparin in the Anticoagulation Therapy of Extracorporeal Membrane Oxygenation: A Systematic Review and Meta-Analysis. Front Pharmacol 2022; 13:771563
247. Marquardt S, Hirschy R, Buyle S: BIVALIRUDIN VERSUS HEPARIN FOR ANTICOAGULATION IN EXTRACORPOREAL MEMBRANE OXYGENATION. Crit Care Med 2023; 51:381
248. Martucci G, Giani M, Schmidt M, et al.: Bleeding during venovenous extracorporeal membrane oxygenation. Preliminary data from the PROTECMO study. Intensive Care Med Exp 2022; 10

249. Mazzeffi M, Lin D, Gonzalez-Almada A, et al.: Outcomes of heparinized adult veno-arterial extracorporeal membrane oxygenation patients managed with low and high activated partial thromboplastin time targets: A systematic review and meta-analysis. Perfusion 2023 doi:10.1177/02676591221150880
250. Navaei A, Kostousov V, Teruya J: Is it time to switch to bivalirudin for ECMO anticoagulation? Frontiers in Medicine 2023; 10
251. Nepomuceno R, Malik MI, Lazo-Langner A, Nagpal AD: Anticoagulation strategies and outcomes during extracorporeal membrane oxygenation support: a single-centre pre-COVID-19 experience. Can J Anesth 2023; 70:458–60
252. Netley J, Roy J, Greenlee J, Hart S, Todt M, Statz B: Bivalirudin Anticoagulation Dosing Protocol for Extracorporeal Membrane Oxygenation: A Retrospective Review. J Extra Corpor Technol 2018; 50:161–6
253. Pang S, Miao G, Zhao X: Effects and safety of extracorporeal membrane oxygenation in the treatment of patients with ST-segment elevation myocardial infarction and cardiogenic shock: A systematic review and meta-analysis. Front Cardiovasc Med 2022; 9
254. Piwowarczyk P, Porzak M, Szczukocka M, et al.: Challenges and pitfalls of extracorporeal membrane oxygenation in critically-ill pregnant and peripartum women with COVID-19: a retrospective case series. International Journal of Obstetric Anesthesia 2023; 53:103625
255. Powell EK, Reynolds TS, Webb JK, et al.: Early veno-venous extracorporeal membrane oxygenation is an effective strategy for traumatically injured patients presenting with refractory respiratory failure. J Trauma Acute Care Surg 2023; 95:S50–9
256. Rajsic S, Breitkopf R, Oezpeker UC, Bukumirić Z, Dobesberger M, Treml B: The Role of Excessive Anticoagulation and Missing Hyperinflammation in ECMO-Associated Bleeding. Journal of Clinical Medicine 2022; 11:2314
257. Rajsic S, Breitkopf R, Treml B, et al.: Association of aPTT-Guided Anticoagulation Monitoring with Thromboembolic Events in Patients Receiving V-A ECMO Support: A Systematic Review and Meta-Analysis. J Clin Med 2023; 12
258. Rajsic S, Treml B, Jadzic D, et al.: Extracorporeal membrane oxygenation for cardiogenic shock: a meta-analysis of mortality and complications. Ann Intensive Care 2022; 12
259. Rajsic S, Treml B, Jadzic D, et al.: aPTT-guided anticoagulation monitoring during ECMO support: A systematic review and meta-analysis. J Crit Care 2023; 77:154332
260. Rajsic S, Breitkopf R, Rugg C, Bukumiric Z, Reitbauer J, Treml B: Thrombotic events develop in 1 out of 5 patients during ECMO support: An 11-year referral center experience. Anasthesiol Intensivmed 2023; 64:S172–3
261. Renaudier M, Roux Q de, Bougouin W, et al.: Acute mesenteric ischaemia in refractory shock on veno-arterial extracorporeal membrane oxygenation. European Heart Journal Acute Cardiovascular Care 2021; 10:62–70
262. Saeed O, Farooq M, Kuntzman M, et al.: Outcomes With Direct and Indirect Thrombin Inhibition During Extracorporeal Membrane Oxygenation for COVID-19. ASAIO Journal 2022; 68:1428
263. Saeed O, Tatooles AJ, Farooq M, et al.: Characteristics and outcomes of patients with COVID-19 supported by extracorporeal membrane oxygenation: A retrospective multicenter study. J Thorac Cardiovasc Surg 2022; 163:2107-2116.e6
264. Sanfilippo F, Currò JM, La Via L, et al.: Use of nafamostat mesilate for anticoagulation during extracorporeal membrane oxygenation: A systematic review. Artificial Organs 2022; 46:2371–81
265. Sanfilippo F, La Via L, Murabito P, Pappalardo F, Astuto M: More evidence available for the use of Bivalirudin in patients supported by extracorporeal membrane oxygenation. Thromb Res 2022; 211:148–9

266. Schaefer A-K, Latus M, Riebandt J, et al.: Bleeding and thrombotic events in post-cardiotomy extracorporeal life support. Eur J Cardio-thorac Surg 2023; 63
267. Shah A, Pasrija C, Kronfli A, et al.: A comparison of anticoagulation strategies in venovenous extracorporeal membrane oxygenation. Crit Care Med 2016; 44:93
268. Slampak-Cindric A, Demerdash A, Stamm J, Mehta Y, Berger A, Gajkowski E: ANTICOAGULATION IN ADULT ECMO: PERCENTAGE OF TIME IN THERAPEUTIC RANGE WITH HEPARIN OR BIVALIRUDIN. Crit Care Med 2023; 51:396
269. Stammers AH, Tesdahl EA, Barletti S, et al.: Anticoagulant Use During Extracorporeal Membrane Oxygenation Using Heparin and Direct Thrombin Inhibitors in COVID-19 and ARDS Patients. J Extra Corpor Technol 2022; 54:223–34
270. Stern J, Dupuis C, Kpeglo H, et al.: Upper gastrointestinal bleeding in adults treated with veno-arterial extracorporeal membrane oxygenation: a cohort study. European Journal of Cardio-Thoracic Surgery 2023; 63:ezad083
271. Sun J, Ma Y, Su W, et al.: Comparison of anticoagulation monitoring strategies for adults supported on extracorporeal membrane oxygenation: A systematic review. Heart Lung 2023; 61:72–83
272. Uricchio MN, Ramanan R, Esper SA, et al.: Bivalirudin Versus Unfractionated Heparin in Patients With Cardiogenic Shock Requiring Venoarterial Extracorporeal Membrane Oxygenation. ASAIO Journal 2023; 69:107
273. Vale C, Worku E: Bivalirudin during extracorporeal support - a retrospective case series. Perfusion 2023; 38:166–7
274. Widmeier E, Wengenmayer T, Maier S, et al.: Extracorporeal membrane oxygenation during the first three waves of the coronavirus disease 2019 pandemic: A retrospective single-center registry study. Artif Organs 2022; 46:1876–85
275. Wieruszewski PM, Macielak SA, Nei SD, et al.: Heparin Versus Bivalirudin for Anticoagulation in Adult Extracorporeal Membrane Oxygenation: A Systematic Review and Meta-Analysis. ASAIO J 2023; 69:137–44
276. Willers A, Swol J, Kuijk SMJ van, et al.: HEROES V-V—HEmorRhagic cOmplications in Veno-Venous Extracorporeal life Support—Development and internal validation of multivariable prediction model in adult patients. Artificial Organs 2022; 46:932–52
277. Worku ET, Win AM, Parmar D, Anstey C, Shekar K: Haematological Trends and Transfusion during Adult Extracorporeal Membrane Oxygenation: A Single Centre Study. J Clin Med 2023; 12
278. Wu X, Li M, Cai Y, et al.: Risk factors for intracranial hemorrhage and mortality in adult patients with severe respiratory failure managed using veno-venous extracorporeal membrane oxygenation. Chin Med J 2022; 135:36–41
279. Yajun Q, Yingying H, Yang L, et al.: Application of extracorporeal membrane oxygenation in critically ill pregnant women. Chin Crit Care Med 2021; 33:605–8
280. Zhang J, Merrick B, Correa GL, et al.: Veno-venous extracorporeal membrane oxygenation in coronavirus disease 2019: a case series. ERJ Open Research 2020; 6
281. Zhang L, Lin C, Liu L, Wang X: A systematic review and meta-analysis of anticoagulation practices and mortality in extracorporeal cardiopulmonary resuscitation. Asian Journal of Surgery 2022; 45:3003–4
282. Zhang Z, Tang X, Wang R, et al.: Application of argatroban in patients receiving extracorporeal membrane oxygenation support: a case-control study. Chin Crit Care Med 2022; 34:1305–10

283. Zhao L, Wang C, Chen JY, et al.: [A single-center experience of venous thromboembolism after adult lung transplantation]. Chung Hua Chieh Ho Ho Hu Hsi Tsa Chih 2019; 42:694–9
284. Zichichi AC, Hohlfelder B, Torbic H, et al.: Systemic Versus Conservative Anticoagulation in Venovenous Extracorporeal Membrane Oxygenation. Am J Ther 2023; 30:E265–8
285. Basken R, Cosgrove R, Malo J, et al.: Predictors of Oxygenator Exchange in Patients Receiving Extracorporeal Membrane Oxygenation. J Extra Corpor Technol 2019; 51:61–6
286. Bikdeli B, Talasaz AH, Rashidi F, et al.: Intermediate-Dose versus Standard-Dose Prophylactic Anticoagulation in Patients with COVID-19 Admitted to the Intensive Care Unit: 90-Day Results from the INSPIRATION Randomized Trial. Thrombosis and haemostasis 2022; 122:131
287. Chan EG, Deitz RL, Ryan JP, et al.: Bloodless lung transplantation: Comparison between 2 central venoarterial extracorporeal membrane oxygenation anticoagulation strategies and their impact on lung transplant outcomes. J Thorac Cardiovasc Surg 2024 doi:10.1016/j.jtcvs.2024.09.055
288. Dorsey M, Phillips K, James L, et al.: Decreased bleeding and thrombotic complications on extracorporeal membrane oxygenation support following an updated anticoagulation protocol. JTCVS Open 2025; 23:199–209
289. Durila M, Vajter J, Garaj M, et al.: Intravenous enoxaparin guided by anti-Xa in venovenous extracorporeal membrane oxygenation: A retrospective, single-center study. Artif Organs 2025; 49:486–96
290. Flinspach AN, Bobyk D, Zacharowski K, Neef V, Raimann FJ: Bleeding Complications in COVID-19 Critically Ill ARDS Patients Receiving VV-ECMO Therapy. J Clin Med 2023; 12
291. Gutierrez A, Kalra R, Steiner M, Marquez A, Yannopoulos D, Bartos J: Outcomes Associated with the Use of Dual Antiplatelet Therapy in Patients Presenting with out of Hospital Cardiac Arrest Supported with Veno Arterial Extracorporeal Membrane Oxygenation. Res Pract Thromb Haemost 2023; 7
292. Halawi H, Harris JE, Goodarzi A, et al.: Use of bivalirudin after initial heparin management among adult patients on long-term venovenous extracorporeal support as a bridge to lung transplant: A case series. Pharmacotherapy 2024; 44:283–9
293. Hickey S, Lee A, Shaikh M-A, et al.: Systematic Review of Major Bleeding for patients with Covid-19 on ECMO. Anesth Analg 2022; 134:300–2
294. Juthani B, Hilaire CST, Auvil B, Mattiola R, Pechulis R, Wu JK: Outcomes of Adult Venovenous Extracorporeal Membrane Oxygenation Patients without Anticoagulation: A Retrospective Review at a Tertiary Level Referral Center. J Am Coll Surg 2016; 223:S24
295. Kasuya S, Aso S, Takahashi I: Effects of shorter activated coagulation time on hemorrhage during venoarterial extracorporeal membrane oxygenation. Ann clin epidemiol 2023; 5:74–9
296. Khoury F, Bartlett A, Li J, Chiang I-H: Low vs High aPTT-Guided Bivalirudin Anticoagulation in ECMO: Single- Center Retrospective Review of Outcomes. ASAIO J 2024; 70:67–8
297. Kuzmin B, Wacker M, Ponomarenko J, et al.: Anticoagulation management during veno-venous ECMO support because of ARDS: Single-center experience. Heliyon 2024; 10:e40417
298. Miller W, Braaten J, Rauzi A, et al.: Thromboembolic Complications in Continuous Versus Interrupted Anticoagulation During Venovenous Extracorporeal Membrane Oxygenation: A Multicenter Study. Crit care explor 2024; 6:e1155
299. Naser M, Alshammari F, Alqahtani M, et al.: ECMO for polytrauma patients: a blessing in disguise or a trail of fiction. ASAIO J 2024; 70:33

300. Qi Z, Gu S, Yu X, et al.: The impact of early perioperative heparin-free anticoagulation for extracorporeal membrane oxygenation on bleeding and thrombotic events in lung transplantation: a retrospective cohort study. Ther Adv Respir Dis 2024; 18
301. Sather K, Wothe J, Rauzi A, et al.: Thrombotic and Bleeding Complications in Continuous vs Intermittent Anticoagulation in Extracorporeal Membrane Oxygenation (ECMO): A Multi-Center Experience. J Am Coll Surg 2023; 237:S574
302. Schiavoni L, Mattei A, Cuccarelli M, et al.: Anticoagulation Management for Veno-Venous ECMO in COVID-19 Patients: Argatroban as Rescue Therapy in Heparin-Associated Thrombocytopenia. J Clin Med 2024; 13
303. Vlok R, Buscher H, Delaney A, et al.: Anticoagulation and associated complications in veno-arterial extracorporeal membrane oxygenation in adult patients: A systematic review and meta-analysis. Crit Care Resusc 2024; 26:332–63
304. Ward J, Ciapala S, Lukas J, et al.: Thrombotic and Hemorrhagic Outcomes in Venovenous Extracorporeal Membrane Oxygenation with or without Systemic Anticoagulation. ASAIO J 2024; 70:44
