# Supplementary material for: Anticoagulation Strategies in Adults Undergoing Extracorporeal Membrane Oxygenation: A Systematic Review and Meta‐Analysis
Source: Artif Organs. 2026 May 20;50(8):1103–17. doi: 10.1111/aor.70162 (PMC13452911; doi:10.1111/aor.70162)

Additional File 7: Forest plots for efficacy, mortality, and bleeding by intervention low Risk of Bias

A Forest Plots Efficacy low RoB

Study	Treatment Events	Treatment Total	UFH Events	UFH Total	Weight	OR	95% CI
**DTI**							
Fisser 2021	13	39	16	78	16.7%	1.94	[0.82; 4.59]
Rivosecchi 2021	23	133	53	162	22.2%	0.43	[0.25; 0.75]
Saeed 2022	31	160	50	251	23.2%	0.97	[0.59; 1.59]
Uricchio 2023	11	54	31	89	17.9%	0.48	[0.22; 1.06]
Seelhammer 2021	13	110	38	223	20.0%	0.65	[0.33; 1.28]
**Random effects model**		**496**		**803**	**100.0%**	**0.74**	**[0.45; 1.20]**
Heterogeneity: $I^2 = 63\%$, $\tau^2 = 0.1931$, $\chi^2_4 = 10.78$ ($p = 0.03$)							
**LMWH**							
Gratz 2020	16	80	11	22	55.1%	0.25	[0.09; 0.68]
Wiegele 2022	5	62	8	36	44.9%	0.31	[0.09; 1.02]
**Random effects model**		**142**		**58**	**100.0%**	**0.27**	**[0.13; 0.59]**
Heterogeneity: $I^2 = 0\%$, $\tau^2 = 0$, $\chi^2_1 = 0.07$ ($p = 0.80$)							
**Nafamostat**							
Lim 2016	1	119	10	201	100.0%	0.16	[0.02; 1.28]
Test for subgroup differences: $\chi^2_2 = 5.89$, df = 2 ($p = 0.05$)							

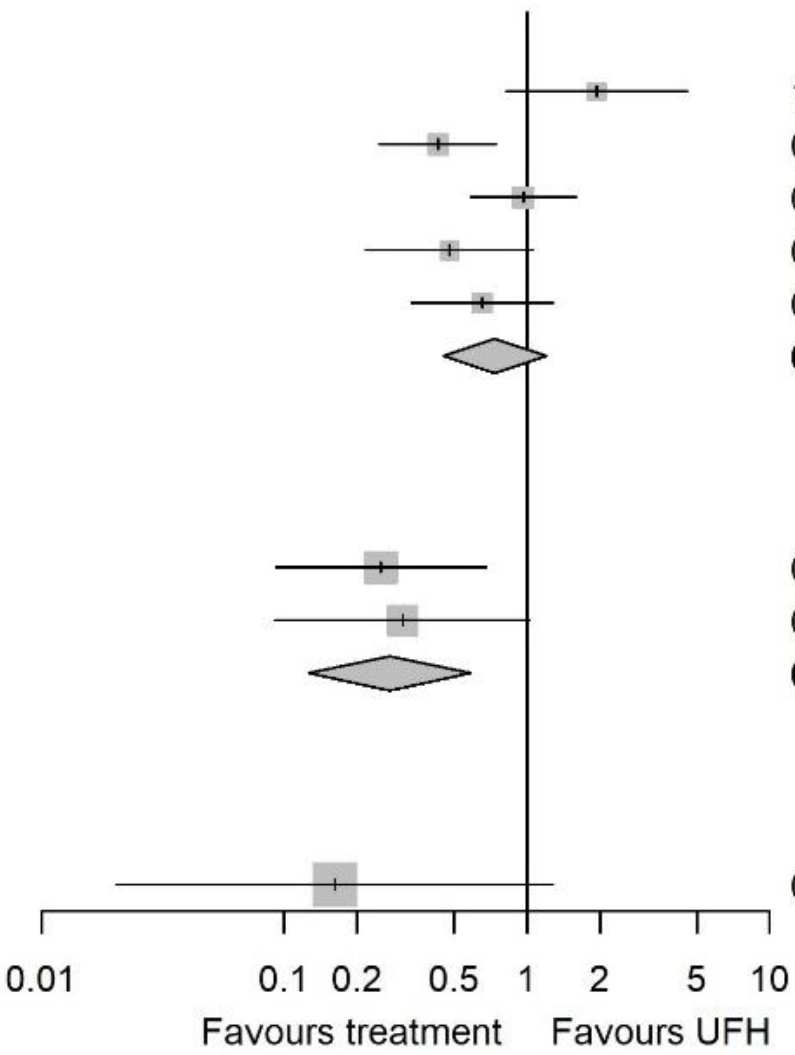

B Forest Plots Mortality low RoB

Study	Treatment Events	Treatment Total	UFH Events	UFH Total	Weight	OR	95% CI
**DTI**							
Fisser 2021	9	39	31	78	14.8%	0.45	[0.19; 1.09]
Rivosecchi 2021	46	133	94	162	21.9%	0.38	[0.24; 0.61]
Saeed 2022	70	160	118	251	23.3%	0.88	[0.59; 1.31]
Uricchio 2023	30	54	47	89	18.0%	1.12	[0.57; 2.20]
Seelhammer 2021	42	110	118	223	22.0%	0.55	[0.34; 0.88]
**Random effects model**		**496**		**803**	**100.0%**	**0.63**	**[0.42; 0.93]**
Heterogeneity: $I^2 = 62\%$, $\tau^2 = 0.1178$, $\chi^2_4 = 10.49$ ($p = 0.03$)							
**LMWH**							
Gratz 2020	7	80	5	22	39.8%	0.33	[0.09; 1.15]
Wiegele 2022	28	62	11	36	60.2%	1.87	[0.79; 4.46]
**Random effects model**		**142**		**58**	**100.0%**	**0.83**	**[0.15; 4.59]**
Heterogeneity: $I^2 = 80\%$, $\tau^2 = 1.2213$, $\chi^2_1 = 5$ ($p = 0.03$)							
**Nafamostat**							
Lim 2016	67	119	98	201	100.0%	1.35	[0.86; 2.14]
Test for subgroup differences: $\chi^2_2 = 6.34$, df = 2 ($p = 0.04$)							

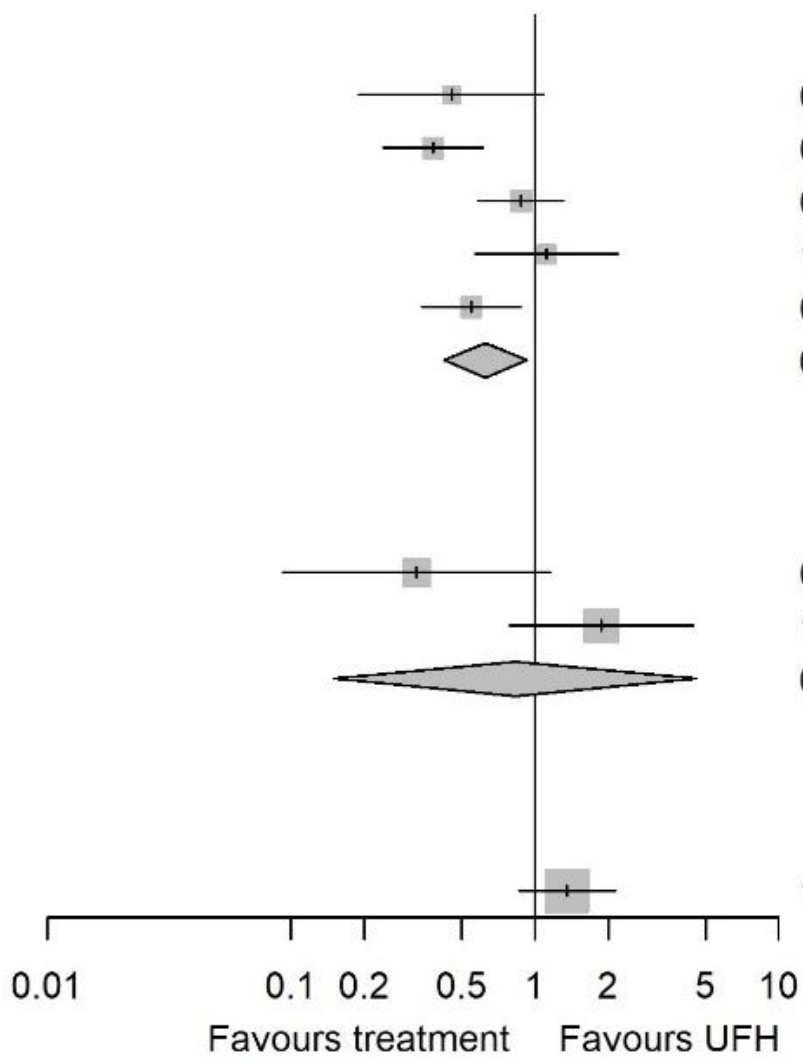

C Forest Plots Bleeding low RoB

Study	Treatment Events	Treatment Total	UFH Events	UFH Total	Weight	OR	95% CI
**DTI**							
Fisser 2021	27	39	63	78	19.0%	0.54	[0.22; 1.30]
Rivosecchi 2021	23	133	53	162	28.4%	0.43	[0.25; 0.75]
Saeed 2022	127	160	224	251	28.5%	0.46	[0.27; 0.81]
Uricchio 2023	23	54	57	89	24.1%	0.42	[0.21; 0.83]
**Random effects model**		**386**		**580**	**100.0%**	**0.45**	**[0.33; 0.62]**
Heterogeneity: $I^2 = 0\%$, $\tau^2 = 0$, $\chi^2_3 = 0.23$ ($p = 0.97$)							
**LMWH**							
Gratz 2020	10	80	5	22	47.5%	0.49	[0.15; 1.61]
Wiegele 2022	7	62	8	36	52.5%	0.45	[0.15; 1.35]
**Random effects model**		**142**		**58**	**100.0%**	**0.46**	**[0.21; 1.05]**
Heterogeneity: $I^2 = 0\%$, $\tau^2 = 0$, $\chi^2_1 = 0.01$ ($p = 0.92$)							
**Nafamostat**							
Lim 2016	15	119	14	201	100.0%	1.93	[0.89; 4.15]
Test for subgroup differences: $\chi^2_2 = 11.94$, df = 2 ($p < 0.01$)							

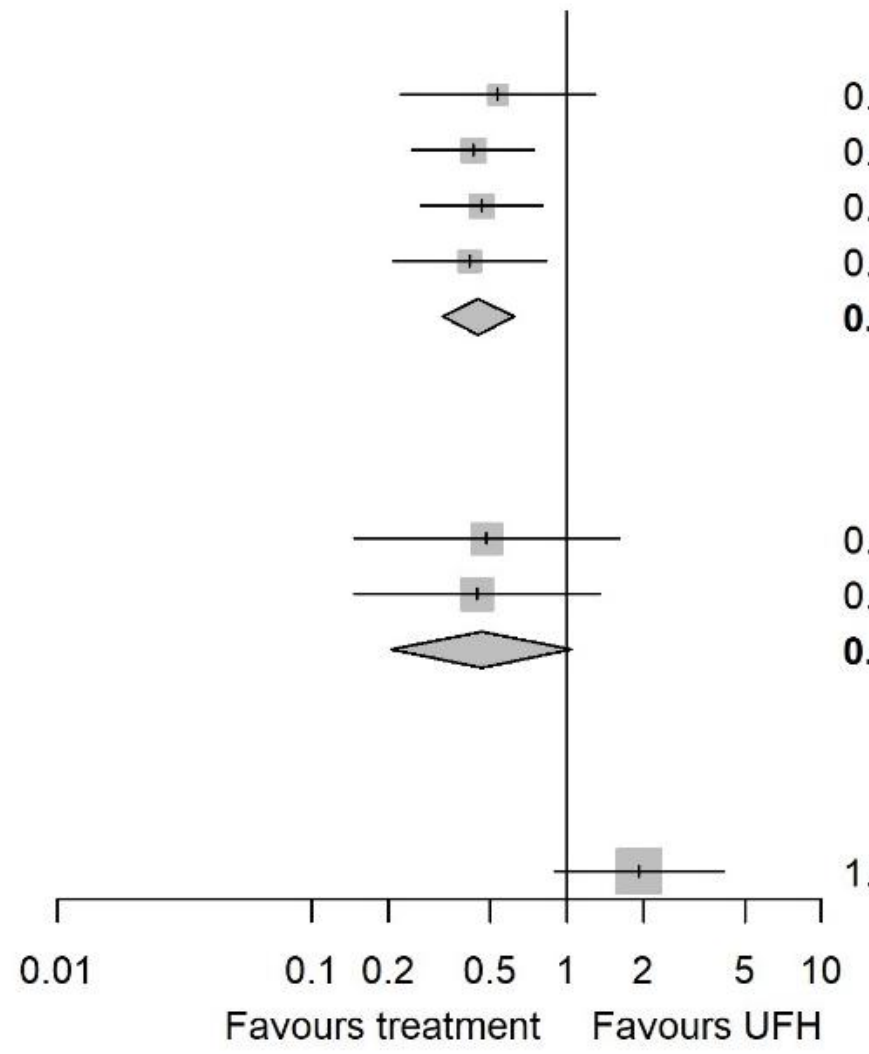

Supplement: Supplementary file 2 [file AOR-50-1103-s003.pdf]
