# Supplementary material for: Anticoagulation Strategies in Adults Undergoing Extracorporeal Membrane Oxygenation: A Systematic Review and Meta‐Analysis
Source: Artif Organs. 2026 May 20;50(8):1103–17. doi: 10.1111/aor.70162 (PMC13452911; doi:10.1111/aor.70162)

5 A Forest Plots for efficacy by intervention: Intracorporeal thrombosis

Study	Treatment Events	Treatment Total	UFH Events	UFH Total	Weight	OR	95% CI
**DTI**							
Berei 2018	8	44	6	28	8.0%	0.81	[0.25; 2.66]
Cho 2021	0	11	2	24	1.2%	0.39	[0.02; 8.85]
Fisser 2021	11	39	13	78	12.9%	1.96	[0.79; 4.91]
Kaseer 2020	1	19	3	33	2.1%	0.56	[0.05; 5.75]
Saeed 2022	31	160	50	251	37.3%	0.97	[0.59; 1.59]
Seelhammer 2021	13	110	38	223	22.5%	0.65	[0.33; 1.28]
Sheridan 2022	1	100	0	50	1.1%	1.52	[0.06; 38.05]
Uricchio 2023	2	54	15	89	5.0%	0.19	[0.04; 0.87]
Kartika 2024	6	34	15	61	9.9%	0.66	[0.23; 1.89]
**Random effects model**		**571**		**837**	**100.0%**	**0.83**	**[0.58; 1.18]**
Heterogeneity: $I^2 = 6\%$, $\tau^2 = 0.0279$, $\chi^2_8 = 8.52$ ($p = 0.38$)							
**LMWH**							
Gratz 2020	0	80	2	22	49.8%	0.05	[0.00; 1.10]
Wiegele 2022	2	62	0	36	50.2%	3.02	[0.14; 64.59]
**Random effects model**		**142**		**58**	**100.0%**	**0.39**	**[0.01; 21.44]**
Heterogeneity: $I^2 = 71\%$, $\tau^2 = 5.8763$, $\chi^2_1 = 3.4$ ($p = 0.07$)							
**Nafamostat**							
Han 2011	2	17	3	51	100.0%	2.13	[0.33; 13.99]
**no anticoagulation**							
Raman 2019	0	52	0	50	40.2%	0.96	[0.02; 49.41]
Song 2023	0	32	1	99	59.8%	1.01	[0.04; 25.41]
**Random effects model**		**84**		**149**	**100.0%**	**0.99**	**[0.08; 12.01]**
Heterogeneity: $I^2 = 0\%$, $\tau^2 = 0$, $\chi^2_1 = 0$ ($p = 0.98$)							
Test for subgroup differences: $\chi^2_3 = 1.09$, df = 3 ($p = 0.78$)							

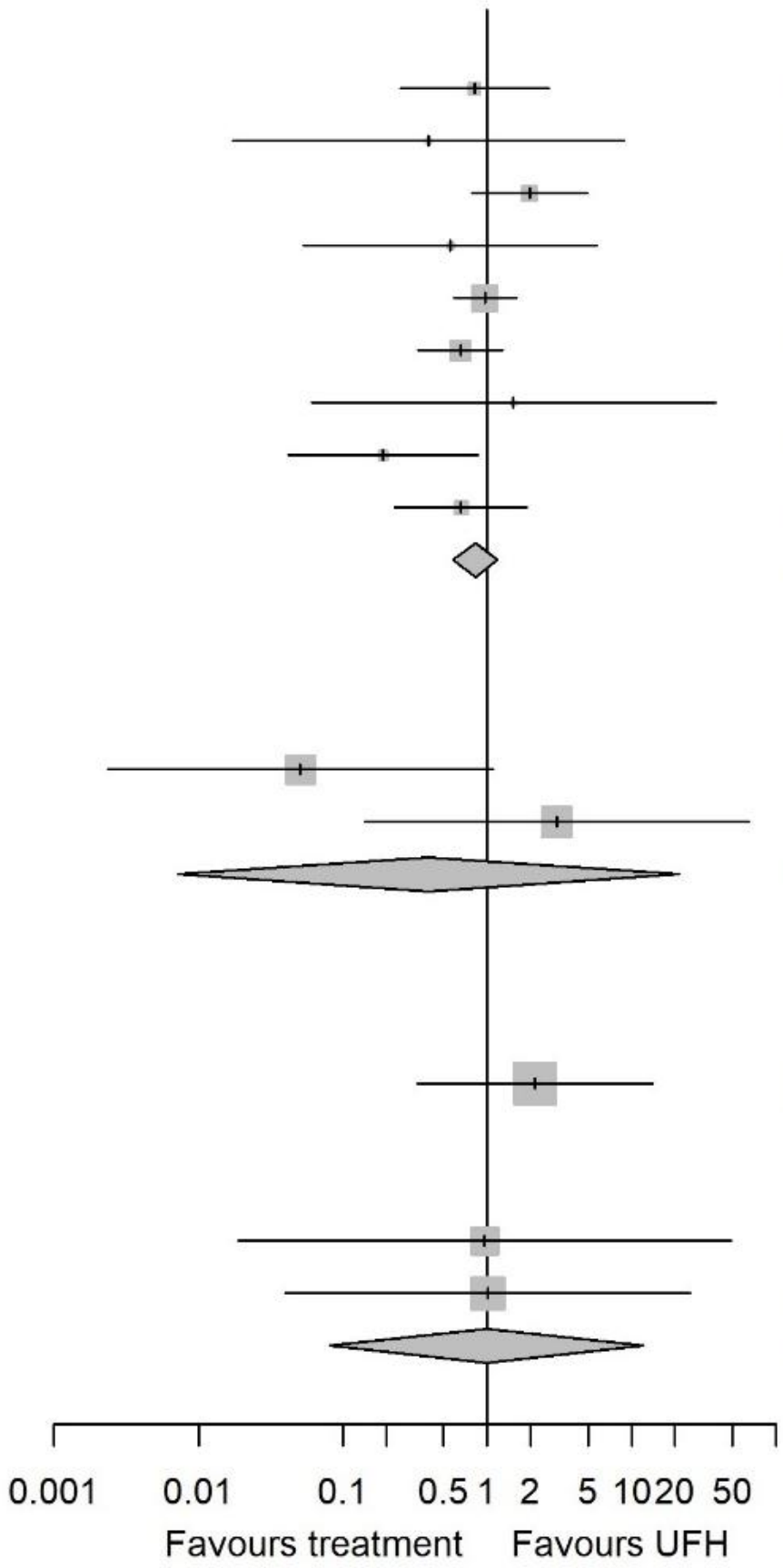

5 B Forest Plots for efficacy by intervention: Extracorporeal thrombosis

Study	Treatment Events	Treatment Total	UFH Events	UFH Total	Weight	OR	95% CI
**DTI**							
Berei 2018	1	44	1	28	2.6%	0.63	[0.04; 10.46]
Cho 2021	6	11	15	24	8.2%	0.72	[0.17; 3.06]
Fisser 2021	11	39	13	78	15.4%	1.96	[0.79; 4.91]
Kaseer 2020	5	19	9	33	9.9%	0.95	[0.27; 3.41]
Rivosecchi 2021	23	133	53	162	24.5%	0.43	[0.25; 0.75]
Sheridan 2022	4	100	2	50	6.1%	1.00	[0.18; 5.65]
Uricchio 2023	9	54	18	89	16.1%	0.79	[0.33; 1.91]
Kartika 2024	10	34	15	61	15.0%	1.28	[0.50; 3.27]
Bursa 2024	0	49	2	14	2.2%	0.05	[0.00; 1.12]
**Random effects model**		**483**		**539**	**100.0%**	**0.81**	**[0.50; 1.31]**
Heterogeneity: $I^2 = 36\%$, $\tau^2 = 0.1690$, $\chi^2_8 = 12.49$ ($p = 0.13$)							
**LMWH**							
Gratz 2020	16	80	9	22	61.3%	0.36	[0.13; 0.99]
Wiegele 2022	3	62	8	36	38.7%	0.18	[0.04; 0.72]
**Random effects model**		**142**		**58**	**100.0%**	**0.28**	**[0.12; 0.64]**
Heterogeneity: $I^2 = 0\%$, $\tau^2 = 0$, $\chi^2_1 = 0.64$ ($p = 0.42$)							
**no anticoagulation**							
Raman 2019	8	52	10	50	59.4%	0.73	[0.26; 2.02]
Song 2023	3	32	11	99	40.6%	0.83	[0.22; 3.17]
**Random effects model**		**84**		**149**	**100.0%**	**0.76**	**[0.34; 1.72]**
Heterogeneity: $I^2 = 0\%$, $\tau^2 = 0$, $\chi^2_1 = 0.02$ ($p = 0.88$)							
Test for subgroup differences: $\chi^2_2 = 4.89$, df = 2 ($p = 0.09$)							

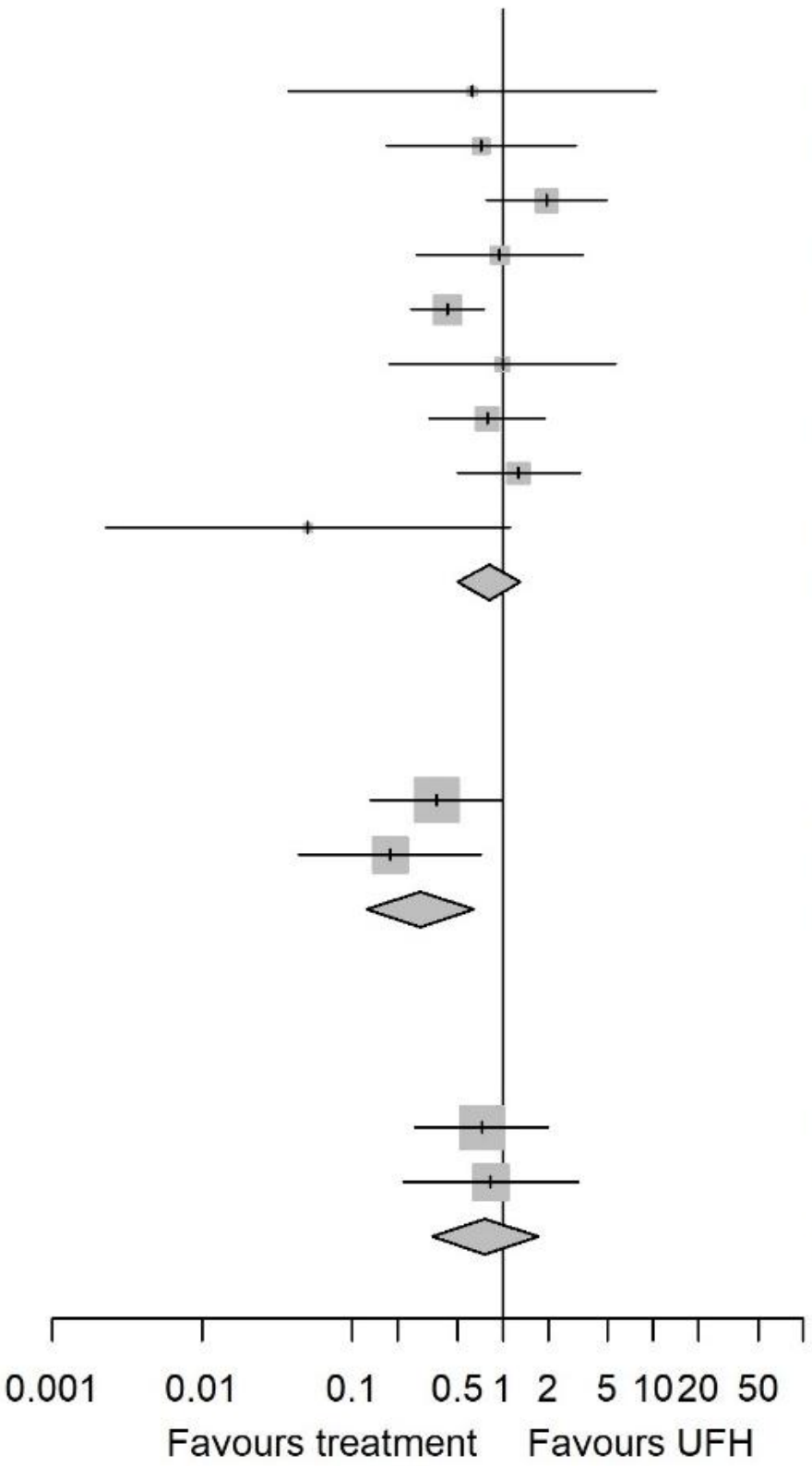

Supplement: Supplementary file 3 [file AOR-50-1103-s004.pdf]
