# Supplementary material for: Anticoagulation Strategies in Adults Undergoing Extracorporeal Membrane Oxygenation: A Systematic Review and Meta‐Analysis
Source: Artif Organs. 2026 May 20;50(8):1103–17. doi: 10.1111/aor.70162 (PMC13452911; doi:10.1111/aor.70162)
Supplement: Supplementary file 4 [file AOR-50-1103-s005.pdf]

Additional File 1: Search strategy used for PubMed/MEDLINE, EMBASE, and CENTRAL.

Ovid MEDLINE(R) ALL 1946 to March 17, 2025

	**Search processing 17-03-2025**	
1	exp Extracorporeal Membrane Oxygenation/	17177
2	(extracorpor* adj3 (oxygen* or membranoxygen*)).ti,ab,kf.	21194
3	('extra corpor*' adj3 oxygen*).ti,ab,kf.	702
4	ecmo*.ti,ab,kf.	15505
5	(extracorpor* adj3 'life support*').ti,ab,kf.	3533
6	('extra corpor*' adj3 'life support*').ti,ab,kf.	93
7	ecls*.ti,ab,kf.	2102
8	(extrapulmon* adj3 (oxygen* or membranoxygen*)).ti,ab,kf.	30
9	('extra pulmon*' adj3 (oxygen* or membranoxygen*)).ti,ab,kf.	1
10	(avecmo* or 'av ecmo*' or 'a v ecmo*').ti,ab,kf.	29
11	(avecls* or 'av ecls*' or 'a-v ecls*').ti,ab,kf.	4
12	(vaecmo* or 'va ecmo*' or 'v-a ecmo*').ti,ab,kf.	2621
13	(vaecls* or 'va ecls*' or 'v a ecls*').ti,ab,kf.	69
14	(vvecmo* or 'vv ecmo*' or 'v v ecmo*').ti,ab,kf.	1708
15	(vvecls* or 'vv ecls*' or 'v v ecls*').ti,ab,kf.	58
16	1 or 2 or 3 or 4 or 5 or 6 or 7 or 8 or 9 or 10 or 11 or 12 or 13 or 14 or 15	29065
17	exp Thrombosis/pc [Prevention & Control]	20918
18	exp Thromboembolism/pc [Prevention & Control]	17536
19	anticoagula*.ti,ab,kf,hw.	164003
20	'anti coagula*'.ti,ab,kf,hw.	3525
21	antithrombotic*.ti,ab,kf,hw.	23924
22	'anti thrombotic*'.ti,ab,kf,hw.	2681
23	exp Anticoagulants/	251166
24	17 or 18 or 19 or 20 or 21 or 22 or 23	336535
25	Heparin/	57958
26	exp Heparin, Low-Molecular-Weight/	14764
27	heparin*.ti,ab,kf,hw,nm.	115656
28	ufh*.ti,ab,kf,hw,nm.	2759
29	lmwh*.ti,ab,kf,hw,nm.	6812
30	exp Enoxaparin/	4093
31	(enoxaparin* or lovenox*).ti,ab,kf,hw,nm.	6932
32	exp Nadroparin/	520
33	(nadroparin* or fraxiparin*).ti,ab,kf,hw,nm.	922
34	exp Dalteparin/	946
35	(dalteparin* or fragmin*).ti,ab,kf,hw,nm.	1704
36	exp Antithrombins/	27517
37	((thrombin* adj3 inhibit*) or antithrombin* or 'anti thrombin*').ti,ab,kf,hw.	32176
38	argatroban*.ti,ab,kf,hw,nm.	1773
39	bivalirudin*.ti,ab,kf,hw,nm.	2085
40	exp Hirudins/	3756
41	hirudin*.ti,ab,kf,hw,nm.	5818
42	25 or 26 or 27 or 28 or 29 or 30 or 31 or 32 or 33 or 34 or 35 or 36 or 37 or 38 or 39 or 40 or 41	151769
43	24 or 42	380160
44	16 and 43	2249
45	limit 44 to "all child (0 to 18 years)"	545
46	limit 44 to "all adult (19 plus years)"	771
47	45 not 46	401
48	44 not 47	1848
49	randomized controlled trial.pt.	633452
50	controlled clinical trial.pt.	95672
51	randomized.ab.	683050
52	placebo.ab.	256690
53	drug therapy.fs.	2789772
54	randomly.ab.	455002
55	trial.ab.	742072
56	groups.ab.	2820510

57	49 or 50 or 51 or 52 or 53 or 54 or 55 or 56	6228539
58	exp animals/ not humans.sh.	5316086
59	57 not 58	5457836
60	48 and 59	340
61	(intervention* adj3 (study or studies or analysis*)).ti,ab,kf.	95686
62	exp Case-Control Studies/ or ('case control*' adj3 (study or studies or analysis*)).ti,ab,kf.	1633579
63	exp Observational Study/ or (observation* adj3 (study or studies or analysis*)).ti,ab,kf,hw.	351735
64	exp Cross-Sectional Studies/ or ('cross sectional*' adj3 (study or studies or analysis*)).ti,ab,kf.	665482
65	exp Cohort Studies/ or (cohort* adj3 (study or studies or analysis*)).ti,ab,kf.	2859813
66	exp Longitudinal Studies/ or (longitudinal* adj3 (study or studies or analysis*)).ti,ab,kf.	267444
67	exp Retrospective Studies/ or (retrospectiv* adj3 (study or studies or analysis*)).ti,ab,kf.	1456523
68	exp Comparative Study/ or (comparativ* adj3 (study or studies or analysis*)).ti,ab,kf.	2111945
69	exp Prospective Studies/ or (prospectiv* adj3 (study or studies or analysis*)).ti,ab,kf.	913132
70	exp Multicenter Study/ or (multicenter* adj3 (study or studies or analysis*)).ti,ab,kf,hw.	417391
71	(epidemiologic* adj3 (study or studies or analysis*)).ti,ab,kf.	123711
72	'follow up*'.ti,ab,kf,hw.	1729758
73	(versus or vs or compar*).ti.	1078171
74	"Meta-Analysis".pt. or exp "Meta-Analysis as Topic"/	240079
75	("meta analys*" or metanalys*).ti,ab,kf.	334959
76	"Systematic Review".pt. or exp "Systematic Reviews as Topic"/	299495
77	(systematic adj3 review*).ti,ab,kf.	389742
78	('major clinical*' adj3 (study or studies or trial* or analysis*)).ti,ab,kf.	1227
79	61 or 62 or 63 or 64 or 65 or 66 or 67 or 68 or 69 or 70 or 71 or 72 or 73 or 74 or 75 or 76 or 77 or 78	7296605
80	48 and 79	717
81	60 or 80	**841**
82	81 and (202310* or 202311* or 202312* or 2024* or 2025*).dt,ez,da.	**148**

Embase

	**Search processing 17-03-2025**	
#108	(#86 OR #106) AND [06-10-2023]/sd NOT [18-03-2025]/sd	**825**
#107	#86 OR #106	**4016**
#106	#50 AND #105	3810
#105	#87 OR #88 OR #89 OR #90 OR #91 OR #92 OR #93 OR #94 OR #95 OR #96 OR #97 OR #98 OR #99 OR #100 OR #101 OR #102 OR #103 OR #104	12135496
#104	'major clinical study'/exp OR (('major clinical*' NEAR/3 (study OR studies OR trial* OR analysis*)):ti,ab,kw)	5635403
#103	(systematic NEAR/3 review*):ti,ab,kw	465340
#102	'systematic review'/exp OR 'systematic review (topic)'/exp	544816
#101	'meta analys*':ti,ab,kw,de OR metanalys*:ti,ab,kw	524833
#100	'meta analysis'/exp OR 'meta analysis (topic)'/exp	401479
#99	versus:ti OR vs:ti OR compar*:ti	1437382
#98	'follow up*':ti,ab,kw,de	2884677
#97	(epidemiologic* NEAR/3 (study OR studies OR analysis*)):ti,ab,kw	162574
#96	'multicenter study'/exp OR ((multicenter* NEAR/3 (study OR studies OR analysis*)):ti,ab,kw,de)	520975
#95	'prospective study'/exp OR ((prospectiv* NEAR/3 (study OR studies OR analysis*)):ti,ab,kw)	1264740
#94	'comparative study'/exp OR ((comparativ* NEAR/3 (study OR studies OR analysis*)):ti,ab,kw)	2004887
#93	'retrospective study'/exp OR ((retrospectiv* NEAR/3 (study OR studies OR analysis*)):ti,ab,kw)	1977106
#92	'longitudinal study'/exp OR ((longitudinal* NEAR/3 (study OR studies OR analysis*)):ti,ab,kw)	328934
#91	'cohort analysis'/exp OR ((cohort* NEAR/3 (study OR studies OR analysis*)):ti,ab,kw)	1450021
#90	'cross-sectional study'/exp OR (('cross sectional*' NEAR/3 (study OR studies OR analysis*)):ti,ab,kw)	810022
#89	'observational study'/exp OR ((observation* NEAR/3 (study OR studies OR analysis*)):ti,ab,kw,de)	586049
#88	'case control study'/exp OR (('case control*' NEAR/3 (study OR studies OR analysis*)):ti,ab,kw)	307220
#87	'intervention study'/exp OR ((intervention* NEAR/3 (study OR studies OR analysis*)):ti,ab,kw)	186992
#86	#50 AND #85	1090
#85	#70 NOT #84	6111331
#84	#71 OR #72 OR #73 OR #74 OR #75 OR #76 OR #77 OR #78 OR #79 OR #80 OR #81 OR #82 OR #83	4757389
#83	'animal experiment'/de NOT ('human experiment'/de OR 'human'/de)	2721807
#82	(rat:ti OR rats:ti OR mouse:ti OR mice:ti OR swine:ti OR porcine:ti OR murine:ti OR sheep:ti OR lambs:ti OR pigs:ti OR piglets:ti OR rabbit:ti OR rabbits:ti OR cat:ti OR cats:ti OR dog:ti OR dogs:ti OR cattle:ti OR bovine:ti OR monkey:ti OR monkeys:ti OR trout:ti OR marmoset*:ti) AND 'animal experiment'/de	1291893

#81	(databases NEAR/4 searched):ab	74591
#80	'update review':ab	153
#79	'we searched':ab AND (review:ti OR review:it)	57013
#78	review:ab AND review:it NOT trial:ti	1266883
#77	('random cluster' NEAR/3 sampl*):ti,ab	1728
#76	'random field*':ti,ab	3128
#75	nonrandom*:ti,ab NOT random*:ti,ab	20087
#74	'systematic review':ti NOT (trial:ti OR study:ti)	316528
#73	'case control*':ti,ab AND random*:ti,ab NOT ('randomised controlled':ti,ab OR 'randomized controlled':ti,ab)	23313
#72	'cross-sectional study'/de NOT ('randomized controlled trial'/de OR 'controlled clinical study'/de OR 'controlled study'/de OR 'randomised controlled':ti,ab OR 'randomized controlled':ti,ab OR 'control group':ti,ab OR 'control groups':ti,ab)	431883
#71	((random* NEXT/1 sampl* NEAR/7 ('cross section*' OR questionnaire* OR survey OR surveys OR database OR databases)):ti,ab) NOT ('comparative study'/de OR 'controlled study'/de OR 'randomised controlled':ti,ab OR 'randomized controlled':ti,ab OR 'randomly assigned':ti,ab)	3037
#70	#51 OR #52 OR #53 OR #54 OR #55 OR #56 OR #57 OR #58 OR #59 OR #60 OR #61 OR #62 OR #63 OR #64 OR #65 OR #66 OR #67 OR #68 OR #69	6944368
#69	trial:ti	457837
#68	'human experiment'/de	685922
#67	volunteer:ti,ab OR volunteers:ti,ab	298096
#66	(controlled NEAR/7 (study OR design OR trial)):ti,ab	499801
#65	assigned:ti,ab OR allocated:ti,ab	537358
#64	((assign* OR match OR matched OR allocation) NEAR/5 (alternate OR group OR groups OR intervention OR interventions OR patient OR patients OR subject OR subjects OR participant OR participants)):ti,ab	453271
#63	crossover:ti,ab OR 'cross over':ti,ab	133332
#62	(parallel NEXT/1 group*):ti,ab	35197
#61	'double blind procedure'/de	229932
#60	((double OR single OR doubly OR singly) NEXT/1 (blind OR blinded OR blindly)):ti,ab	294304
#59	(open NEXT/1 label):ti,ab	122274
#58	(evaluated:ab OR evaluate:ab OR evaluating:ab OR assessed:ab OR assess:ab) AND (compare:ab OR compared:ab OR comparing:ab OR comparison:ab)	3094737
#57	compare:ti OR compared:ti OR comparison:ti	669453
#56	placebo:ti,ab	391054
#55	'intermethod comparison'/de	313058
#54	'randomization'/de	100417
#53	random*:ti,ab	2181104
#52	'controlled clinical study'/de	445176
#51	'randomized controlled trial'/de	872128
#50	#46 NOT #49	8488
#49	#47 NOT #48	1767
#48	#46 AND ([adult]/lim OR [aged]/lim OR [middle aged]/lim OR [very elderly]/lim OR [young adult]/lim)	5620
#47	#46 AND ([adolescent]/lim OR [child]/lim OR [fetus]/lim OR [infant]/lim OR [newborn]/lim OR [preschool]/lim OR [school]/lim)	2291
#46	#16 AND #45	10255
#45	#24 OR #44	999335
#44	#25 OR #26 OR #27 OR #28 OR #29 OR #30 OR #31 OR #32 OR #33 OR #34 OR #35 OR #36 OR #37 OR #38 OR #39 OR #40 OR #41 OR #42 OR #43	340393
#43	hirudin*:ti,ab,kw,de,tn	7581
#42	'hirudin'/exp	4793
#41	bivalirudin*:ti,ab,kw,de,tn	7993
#40	'bivalirudin'/exp	7806
#39	argatroban*:ti,ab,kw,de,tn	6033
#38	'argatroban'/exp	5856
#37	((thrombin* NEAR/3 inhibit*):ti,ab,kw,de) OR antithrombin*:ti,ab,kw,de OR 'anti thrombin*':ti,ab,kw,de	52419
#36	'thrombin inhibitor'/exp	69099
#35	dalteparin*:ti,ab,kw,de,tn OR fragmin*:ti,ab,kw,de,tn	9282
#34	'dalteparin'/exp	9027
#33	nadroparin*:ti,ab,kw,de,tn OR fraxiparin*:ti,ab,kw,de,tn	5543
#32	'nadroparin'/exp	5394

#31	enoxaparin*:ti,ab,kw,de,tn OR lovenox*:ti,ab,kw,de,tn	37247
#30	'enoxaparin'/exp	36505
#29	lmwh*:ti,ab,kw,de,tn	13605
#28	ufh*:ti,ab,kw,de,tn	5121
#27	heparin*:ti,ab,kw,de,tn	262535
#26	'low molecular weight heparin'/exp	88927
#25	'heparin'/exp	183904
#24	#17 OR #18 OR #19 OR #20 OR #21 OR #22 OR #23	959762
#23	'anticoagulant agent'/exp	880089
#22	'anti thrombotic*':ti,ab,kw,de	4716
#21	antithrombotic*:ti,ab,kw,de	40814
#20	'anti coagula*':ti,ab,kw,de	7709
#19	anticoagula*:ti,ab,kw,de	333425
#18	'anticoagulant therapy'/exp	79657
#17	'anticoagulation'/exp	82893
#16	#1 OR #2 OR #3 OR #4 OR #5 OR #6 OR #7 OR #8 OR #9 OR #10 OR #11 OR #12 OR #13 OR #14 OR #15	63636
#15	vvecls*:ti,ab,kw OR 'vv ecls*':ti,ab,kw OR 'v v ecls*':ti,ab,kw	117
#14	vvecmo*:ti,ab,kw OR 'vv ecmo*':ti,ab,kw OR 'v v ecmo*':ti,ab,kw	3947
#13	vaecls*:ti,ab,kw OR 'va ecls*':ti,ab,kw OR 'v a ecls*':ti,ab,kw	175
#12	vaecmo*:ti,ab,kw OR 'va ecmo*':ti,ab,kw OR 'v-a ecmo*':ti,ab,kw	6322
#11	avecls*:ti,ab,kw OR 'av ecls*':ti,ab,kw OR 'a-v ecls*':ti,ab,kw	4
#10	avecmo*:ti,ab,kw OR 'av ecmo*':ti,ab,kw OR 'a v ecmo*':ti,ab,kw	88
#9	('extra pulmon*' NEAR/3 (oxygen* OR membranoxygen*)):ti,ab,kw	1
#8	(extrapulmon* NEAR/3 (oxygen* OR membranoxygen*)):ti,ab,kw	31
#7	ecls*:ti,ab,kw	3609
#6	('extra corpor*' NEAR/3 'life support*'):ti,ab,kw	270
#5	(extracorpor* NEAR/3 'life support*'):ti,ab,kw	5598
#4	ecmo*:ti,ab,kw	32020
#3	('extra corpor*' NEAR/3 oxygen*):ti,ab,kw	1710
#2	(extracorpor* NEAR/3 (oxygen* OR membranoxygen*)):ti,ab,kw	32342
#1	'extracorporeal oxygenation'/exp	52214

EBM Reviews - Cochrane Central Register of Controlled Trials February 2025

	**Search processing 17-03-2025**	
1	exp Extracorporeal Membrane Oxygenation/	366
2	(extracorpor* adj3 (oxygen* or membranoxygen*)).ti,ab,kw.	1090
3	('extra corpor*' adj3 oxygen*).ti,ab,kw.	70
4	ecmo*.ti,ab,kw.	1065
5	(extracorpor* adj3 'life support*').ti,ab,kw.	89
6	('extra corpor*' adj3 'life support*').ti,ab,kw.	4
7	ecls*.ti,ab,kw.	76
8	(extrapulmon* adj3 (oxygen* or membranoxygen*)).ti,ab,kw.	0
9	('extra pulmon*' adj3 (oxygen* or membranoxygen*)).ti,ab,kw.	0
10	(avecmo* or 'av ecmo*' or 'a v ecmo*').ti,ab,kw.	3
11	(avecls* or 'av ecls*' or 'a-v ecls*').ti,ab,kw.	0
12	(vaecmo* or 'va ecmo*' or 'v-a ecmo*').ti,ab,kw.	138
13	(vaecls* or 'va ecls*' or 'v a ecls*').ti,ab,kw.	0
14	(vvecmo* or 'vv ecmo*' or 'v v ecmo*').ti,ab,kw.	58
15	(vvecls* or 'vv ecls*' or 'v v ecls*').ti,ab,kw.	0
16	1 or 2 or 3 or 4 or 5 or 6 or 7 or 8 or 9 or 10 or 11 or 12 or 13 or 14 or 15	1690
17	exp Thrombosis/pc [Prevention & Control]	36
18	exp Thromboembolism/pc [Prevention & Control]	17
19	anticoagula*.ti,ab,kw,hw.	17516
20	'anti coagula*'.ti,ab,kw,hw.	563
21	antithrombotic*.ti,ab,kw,hw.	3085
22	'anti thrombotic*'.ti,ab,kw,hw.	328
23	exp Anticoagulants/	15390
24	17 or 18 or 19 or 20 or 21 or 22 or 23	27033
25	Heparin/	4232

26	exp Heparin, Low-Molecular-Weight/	2648
27	heparin*.ti,ab,kw,hw.	13537
28	ufh*.ti,ab,kw,hw.	815
29	lmwh*.ti,ab,kw,hw.	1733
30	exp Enoxaparin/	1019
31	(enoxaparin* or lovenox*).ti,ab,kw,hw.	2669
32	exp Nadroparin/	158
33	(nadroparin* or fraxiparin*).ti,ab,kw,hw.	420
34	exp Dalteparin/	480
35	(dalteparin* or fragmin*).ti,ab,kw,hw.	966
36	exp Antithrombins/	3159
37	((thrombin* adj3 inhibit*) or antithrombin* or 'anti thrombin*').ti,ab,kw,hw.	2977
38	argatroban*.ti,ab,kw,hw.	198
39	bivalirudin*.ti,ab,kw,hw.	581
40	exp Hirudins/	435
41	hirudin*.ti,ab,kw,hw.	548
42	25 or 26 or 27 or 28 or 29 or 30 or 31 or 32 or 33 or 34 or 35 or 36 or 37 or 38 or 39 or 40 or 41	18754
43	24 or 42	34576
44	16 and 43	191
45	(child* or infan* or newborn* or neonat* or neo-nat* or preschool* or adolescen* or p?ediat* or juvenile or youth*).ti.	169038
46	(adult* or elderly or middle aged or old* aged).ti,ot,kw,hw. or exp adult/	968321
47	45 not 46	146480
48	44 not 47	**175**
49	48 and ("2023*" or "2024*" or "2025*").il.	**28**
