# Supplementary material for: Anticoagulation Strategies in Adults Undergoing Extracorporeal Membrane Oxygenation: A Systematic Review and Meta‐Analysis
Source: Artif Organs. 2026 May 20;50(8):1103–17. doi: 10.1111/aor.70162 (PMC13452911; doi:10.1111/aor.70162)
Supplement: Supplementary file 5 [file AOR-50-1103-s006.pdf]

Additional File 4: Incidence of bleeding events by intervention: Subgroup analysis

Figure 1: Forest Plots for incidence of bleeding events by intervention VA subgroup

Study	Treatment Events	Treatment Total	UFH Events	UFH Total	Weight	OR	95% CI
**DTI**							
Berei 2018	30	44	14	28	48.2%	2.14	[0.81; 5.68]
Uricchio 2023	23	54	57	89	51.8%	0.42	[0.21; 0.83]
**Random effects model**		**98**		**117**	**100.0%**	**0.91**	**[0.18; 4.52]**
Heterogeneity: $I^2 = 86\%$, $\tau^2 = 1.1554$, $\chi^2_1 = 7.21$ ($p < 0.01$)							
**Nafamostat**							
Han 2011	2	17	31	51	43.5%	0.09	[0.02; 0.42]
Lim 2016	15	119	14	201	56.5%	1.93	[0.89; 4.15]
**Random effects model**		**136**		**252**	**100.0%**	**0.44**	**[0.02; 9.24]**
Heterogeneity: $I^2 = 92\%$, $\tau^2 = 4.4315$, $\chi^2_1 = 12.05$ ($p < 0.01$)							
**no anticoagulation**							
Raman 2019	17	52	37	50	68.1%	0.17	[0.07; 0.40]
Song 2023	0	32	10	99	31.9%	0.13	[0.01; 2.30]
**Random effects model**		**84**		**149**	**100.0%**	**0.17**	**[0.07; 0.38]**
Heterogeneity: $I^2 = 0\%$, $\tau^2 = 0$, $\chi^2_1 = 0.03$ ($p = 0.86$)							
Test for subgroup differences: $\chi^2_2 = 3.55$, df = 2 ($p = 0.17$)							

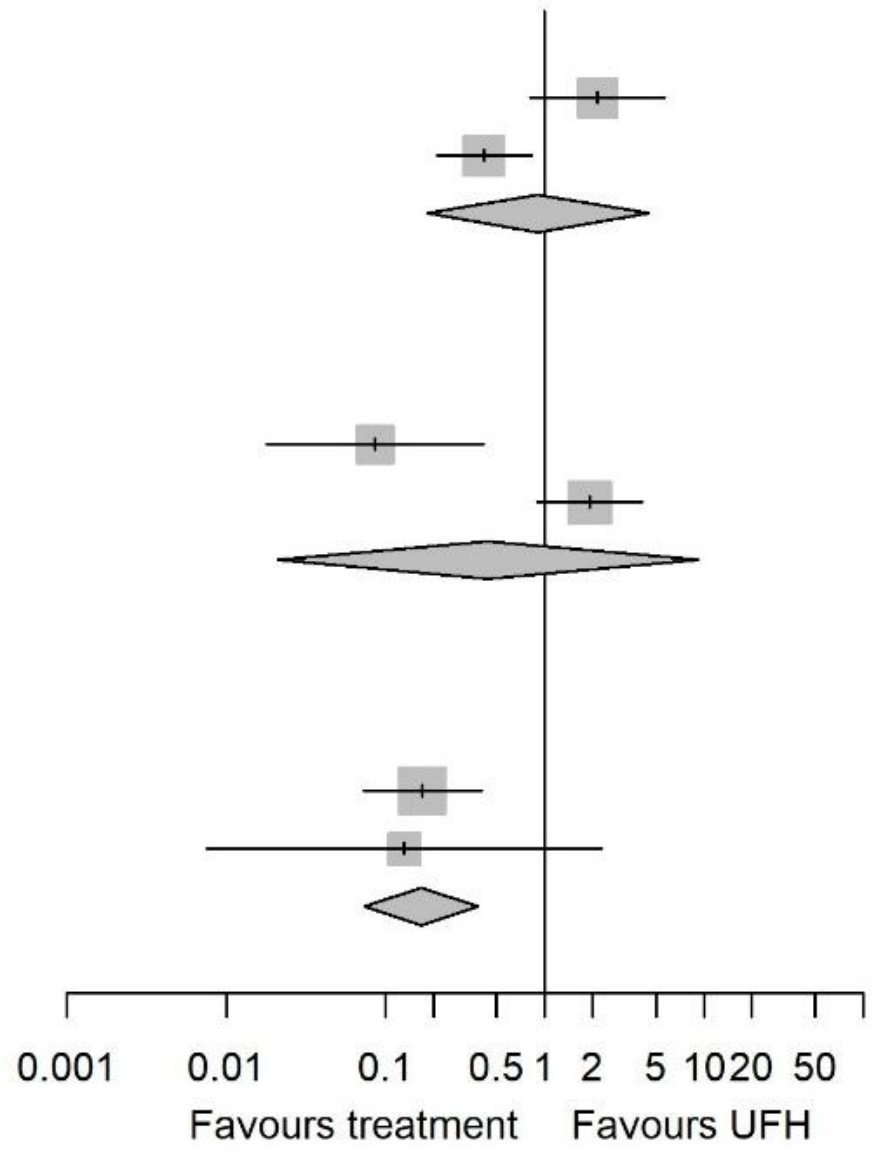

Figure 2: Forest Plots for incidence of bleeding events by intervention VV subgroup

Study	Treatment Events	Treatment Total	UFH Events	UFH Total	Weight	OR	95% CI
**DTI**							
Bermea 2021	3	5	10	25	2.5%	2.25	[0.32; 15.97]
Fisser 2021	27	39	63	78	12.4%	0.54	[0.22; 1.30]
Rivosecchi 2021	23	133	53	162	31.3%	0.43	[0.25; 0.75]
Saeed 2022	127	160	224	251	31.7%	0.46	[0.27; 0.81]
Tong 2023	8	14	17	20	3.7%	0.24	[0.05; 1.19]
Kartika 2024	15	34	35	61	13.6%	0.59	[0.25; 1.37]
Bursa 2024	5	49	5	14	4.7%	0.20	[0.05; 0.86]
**Random effects model**		**434**		**611**	**100.0%**	**0.46**	**[0.34; 0.63]**
Heterogeneity: $I^2 = 0\%$, $\tau^2 = < 0.0001$, $\chi^2_6 = 4.89$ ($p = 0.56$)							
**LMWH**							
Piwowarczyk 2021	12	35	17	32	56.0%	0.46	[0.17; 1.23]
Wiegele 2022	7	62	8	36	44.0%	0.45	[0.15; 1.35]
**Random effects model**		**97**		**68**	**100.0%**	**0.45**	**[0.22; 0.95]**
Heterogeneity: $I^2 = 0\%$, $\tau^2 = 0$, $\chi^2_1 = 0$ ($p = 0.97$)							
Test for subgroup differences: $\chi^2_1 = 0.00$, df = 1 ($p = 0.95$)							

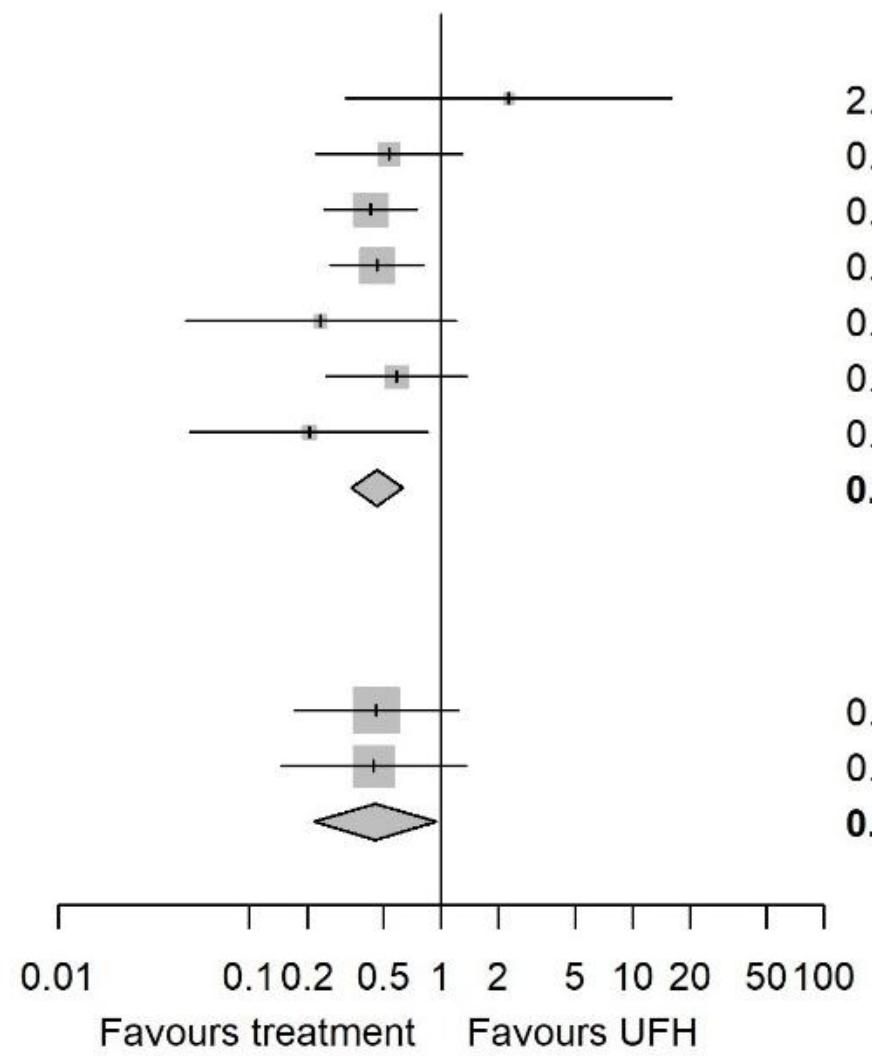

Figure 3: Forest Plots for incidence of bleeding events by intervention mixed subgroup

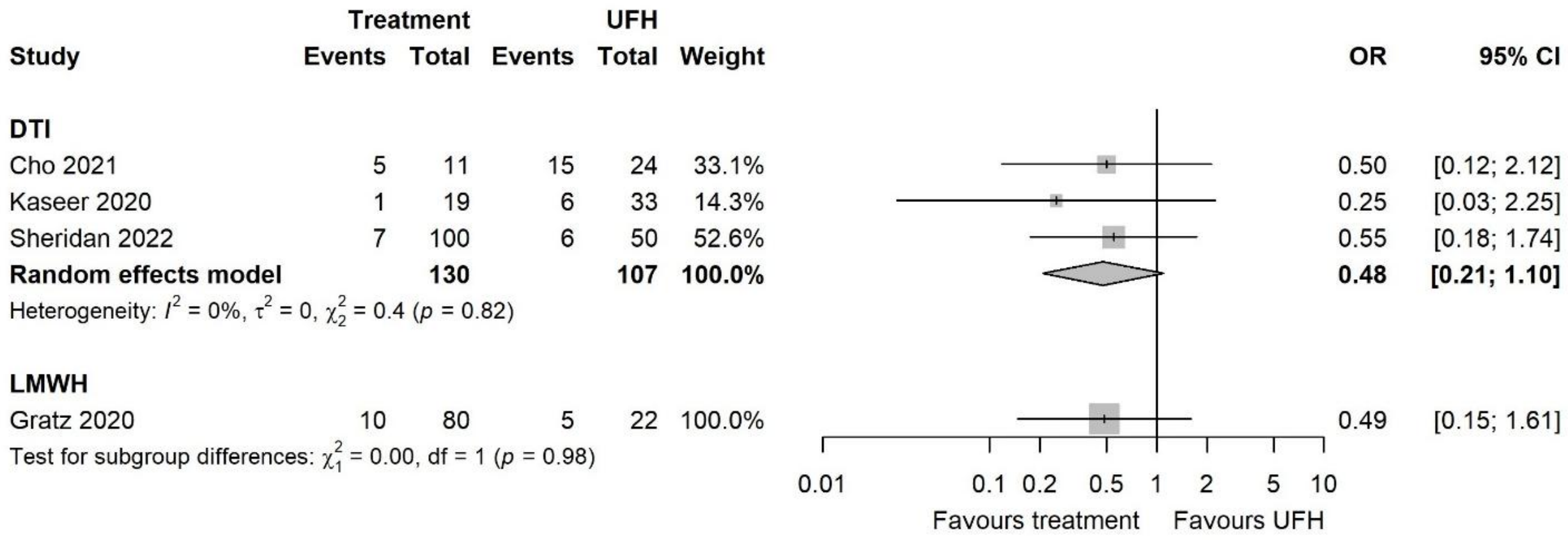

Table 1: Incidence of bleeding events by intervention: Subgroup analysis

Treatment	OR	95% CI LOWER BOUND	95% CI UPPER BOUND	Metaregression p-value
DTI OVERALL	0.51	0.39	0.67	<0.001
DTI VA	0.91	0.18	4.52	
DTI VV	0.46	0.34	0.63	
DTI MIXED	0.48	0.21	1.10	
LMWH OVERALL	0.46	0.25	0.87	0.04
LMWH VV	0.45	0.22	0.95	
LMWH MIXED	0.49	0.15	1.61	
