# Supplementary material for: Anticoagulation Strategies in Adults Undergoing Extracorporeal Membrane Oxygenation: A Systematic Review and Meta‐Analysis
Source: Artif Organs. 2026 May 20;50(8):1103–17. doi: 10.1111/aor.70162 (PMC13452911; doi:10.1111/aor.70162)
Supplement: Supplementary file 6 [file AOR-50-1103-s007.pdf]

Additional File 3: Efficacy by intervention: Subgroup analysis

Figure 1: Forest Plots for efficacy by intervention VA subgroup

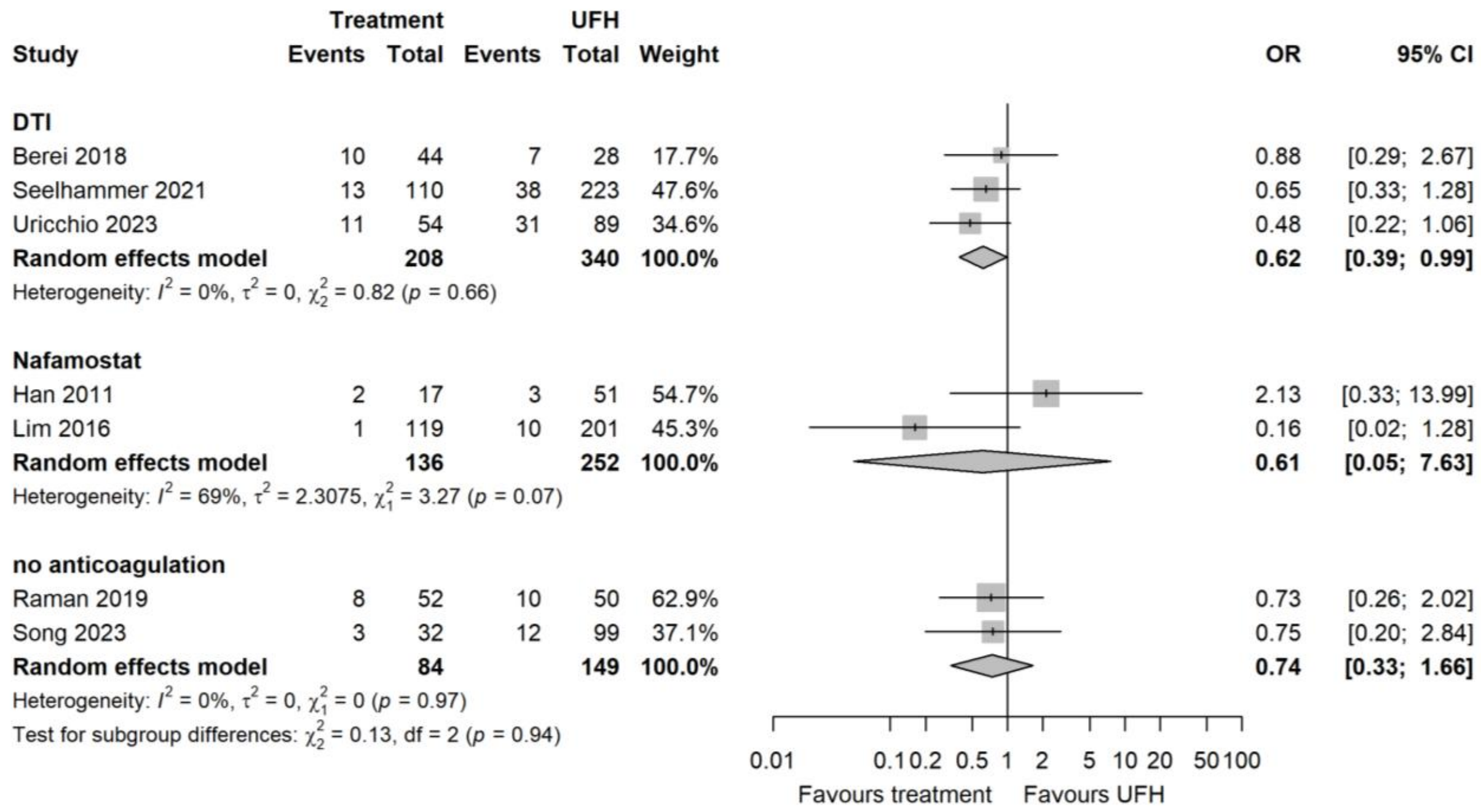

Figure 2: Forest Plots for efficacy by intervention VV subgroup

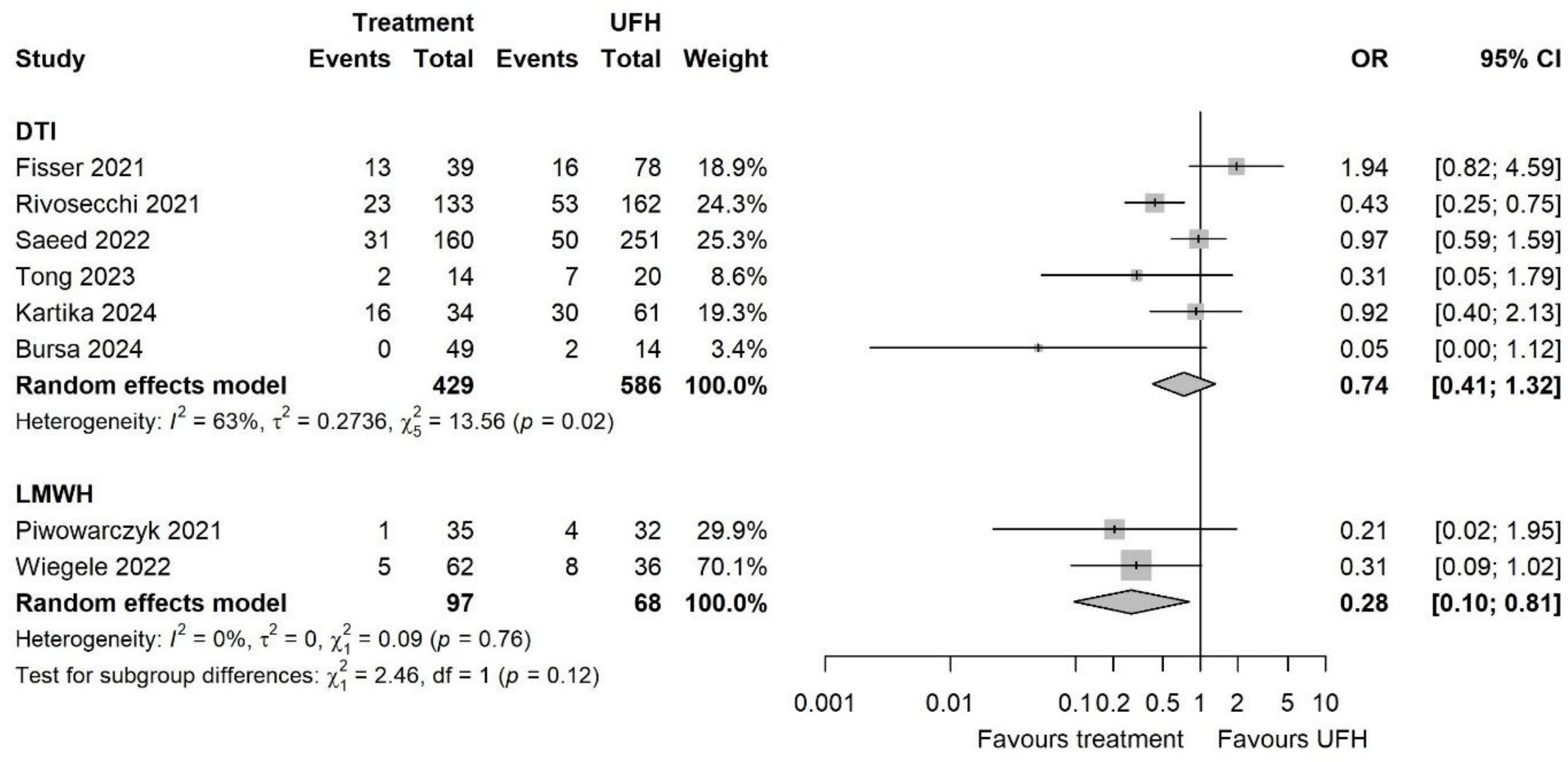

Figure 3: Forest Plots for efficacy by intervention mixed subgroup

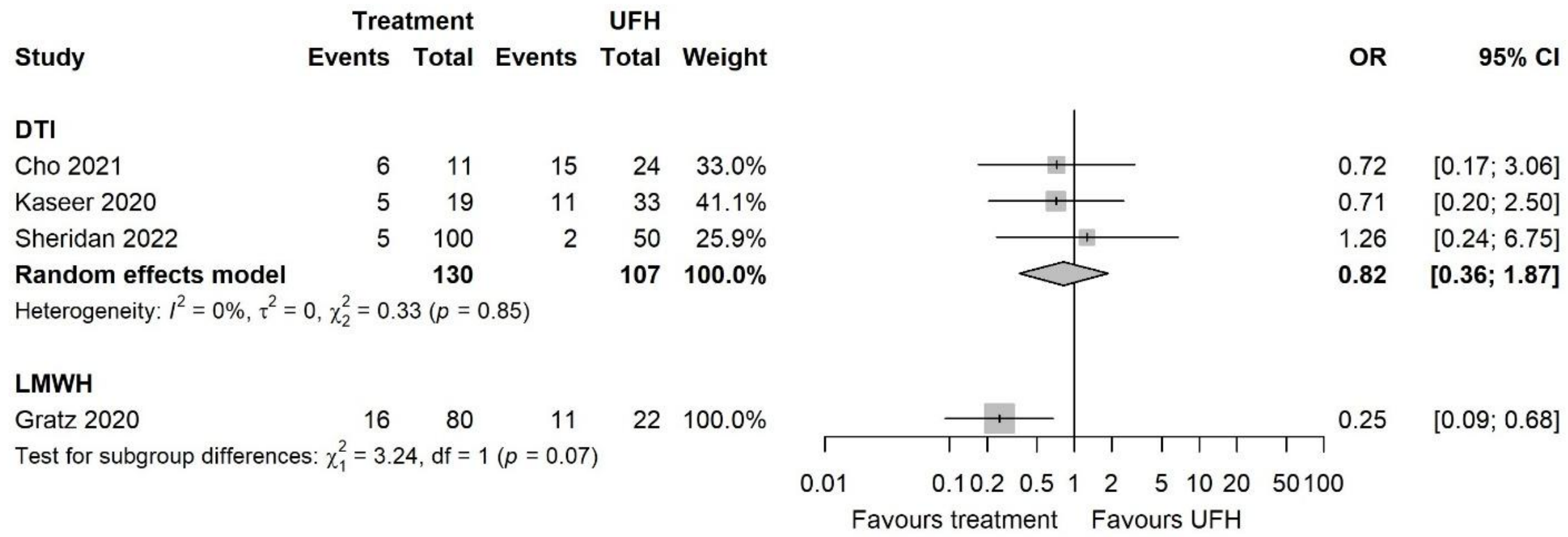

Table 1: Efficacy by intervention: Subgroup analysis

Treatment	OR	95% CI LOWER BOUND	95% CI UPPER BOUND	Metaregression p-value
DTI OVERALL	0.73	0.53	1.00	0.24
DTI VA	0.62	0.39	0.99	
DTI VV	0.74	0.41	1.32	
DTI MIXED	0.82	0.36	1.87	
LMWH OVERALL	0.26	0.13	0.55	0.02
LMWH VV	0.28	0.10	0.81	
LMWH MIXED	0.25	0.09	0.68	
