# Supplementary material for: Anticoagulation Strategies in Adults Undergoing Extracorporeal Membrane Oxygenation: A Systematic Review and Meta‐Analysis
Source: Artif Organs. 2026 May 20;50(8):1103–17. doi: 10.1111/aor.70162 (PMC13452911; doi:10.1111/aor.70162)

Additional File 8: Forest plots for efficacy, mortality, and bleeding by intervention with split Direct Thrombin Inhibitor group

A Forest Plots Efficacy with split DTI

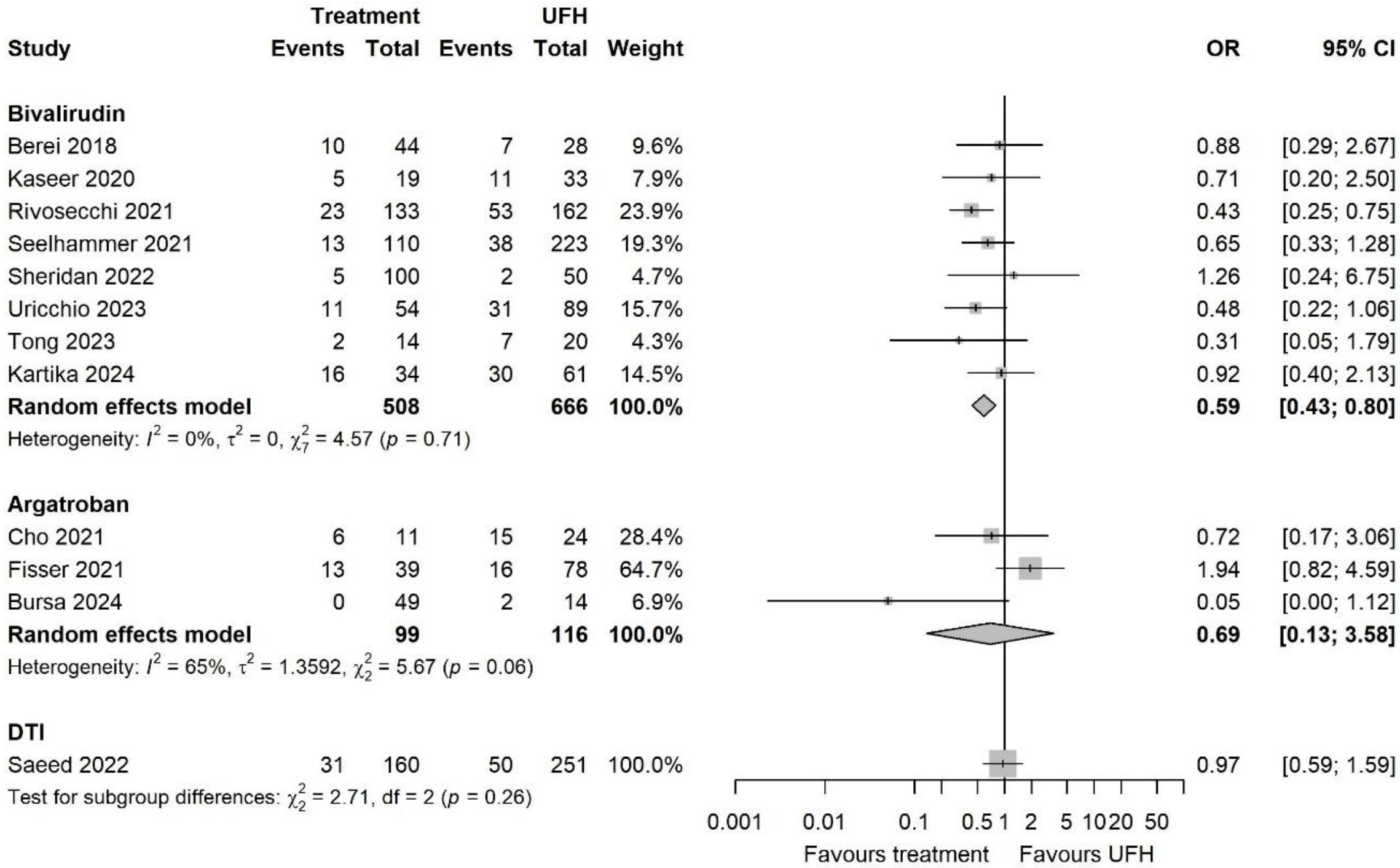

B Forest Plots Mortality with split DTI

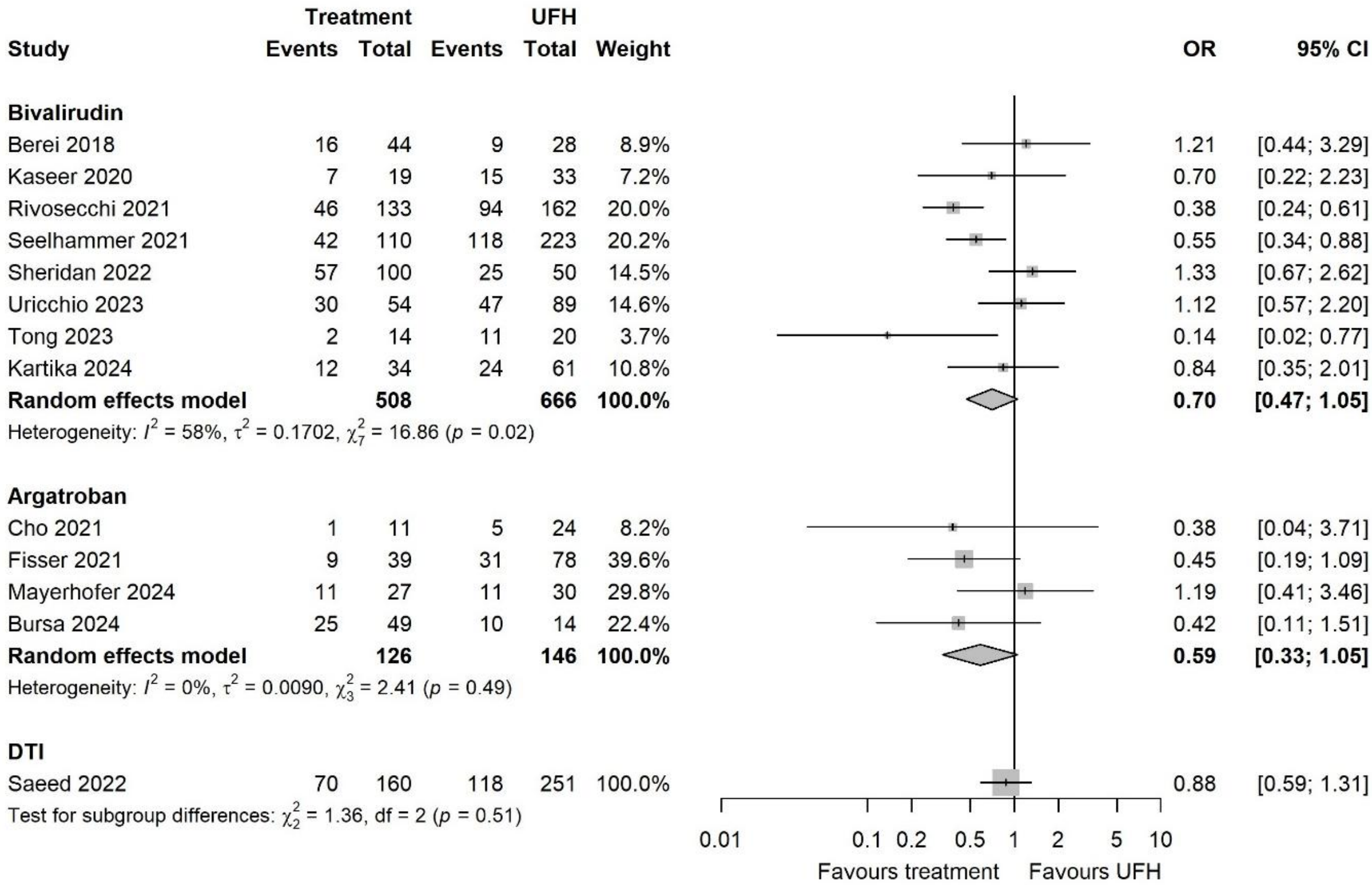

C Forest Plots Bleeding with split DTI

Study	Treatment Events	Treatment Total	UFH Events	UFH Total	Weight	OR	95% CI
**Bivalirudin**							
Berei 2018	30	44	14	28	11.8%	2.14	[0.81; 5.68]
Kaseer 2020	1	19	6	33	2.4%	0.25	[0.03; 2.25]
Rivosecchi 2021	23	133	53	162	34.5%	0.43	[0.25; 0.75]
Sheridan 2022	7	100	6	50	8.6%	0.55	[0.18; 1.74]
Uricchio 2023	23	54	57	89	22.9%	0.42	[0.21; 0.83]
Tong 2023	8	14	17	20	4.3%	0.24	[0.05; 1.19]
Kartika 2024	15	34	35	61	15.6%	0.59	[0.25; 1.37]
**Random effects model**		**398**		**443**	**100.0%**	**0.55**	**[0.34; 0.90]**
Heterogeneity: $I^2 = 42\%$, $\tau^2 = 0.1777$, $\chi^2_6 = 10.36$ ($p = 0.11$)							
**DTI**							
Bermea 2021	3	5	10	25	7.9%	2.25	[0.32; 15.97]
Saeed 2022	127	160	224	251	92.1%	0.46	[0.27; 0.81]
**Random effects model**		**165**		**276**	**100.0%**	**0.76**	**[0.18; 3.22]**
Heterogeneity: $I^2 = 57\%$, $\tau^2 = 0.7069$, $\chi^2_1 = 2.31$ ($p = 0.13$)							
**Argatroban**							
Cho 2021	5	11	15	24	21.5%	0.50	[0.12; 2.12]
Fisser 2021	27	39	63	78	56.6%	0.54	[0.22; 1.30]
Bursa 2024	5	49	5	14	21.9%	0.20	[0.05; 0.86]
**Random effects model**		**99**		**116**	**100.0%**	**0.43**	**[0.22; 0.83]**
Heterogeneity: $I^2 = 0\%$, $\tau^2 = 0$, $\chi^2_2 = 1.31$ ($p = 0.52$)							
Test for subgroup differences: $\chi^2_2 = 0.65$, df = 2 ($p = 0.72$)							

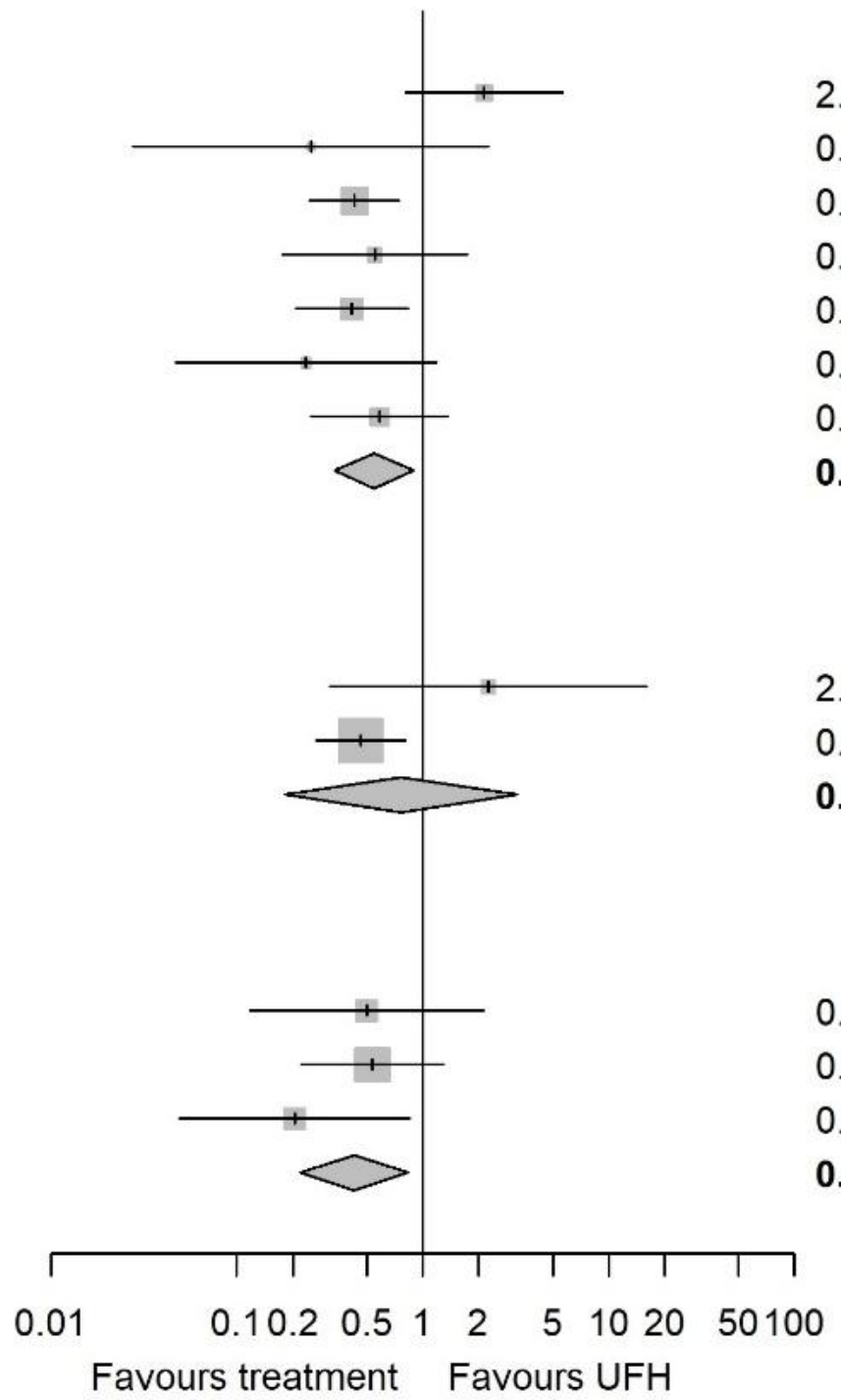

Supplement: Supplementary file 7 [file AOR-50-1103-s008.pdf]
